# A genome-wide screen for variants influencing certolizumab pegol response in a moderate to severe rheumatoid arthritis population

**DOI:** 10.1371/journal.pone.0261165

**Published:** 2022-04-12

**Authors:** Ian R. White, Sarah E. Kleinstein, Christophe Praet, Chris Chamberlain, Duncan McHale, Jessica M. Maia, Pingxing Xie, David B. Goldstein, Thomas J. Urban, Patrick R. Shea

**Affiliations:** 1 Experimental Medicine and Diagnostics, UCB Celltech, Slough, United Kingdom; 2 Institute for Genomic Medicine, Columbia University, New York, New York, United States of America; 3 Department of Molecular Genetics and Microbiology, Duke University Medical Center, Durham, North Carolina, United States of America; 4 UCB Pharma, Braine L’Alleud, Belgium; 5 Faculty of Medicine, McGill University, Montréal, Québec, Canada; 6 Eshelman School of Pharmacy, University of North Carolina, Chapel Hill, North Carolina, United States of America; University of California San Francisco, UNITED STATES

## Abstract

Certolizumab pegol (CZP) is a PEGylated Fc-free tumor necrosis factor (TNF) inhibitor antibody approved for use in the treatment of rheumatoid arthritis (RA), Crohn’s disease, psoriatic arthritis, axial spondyloarthritis and psoriasis. In a clinical trial of patients with severe RA, CZP improved disease symptoms in approximately half of patients. However, variability in CZP efficacy remains a problem for clinicians, thus, the aim of this study was to identify genetic variants predictive of CZP response. We performed a genome-wide association study (GWAS) of 302 RA patients treated with CZP in the REALISTIC trial to identify common single nucleotide polymorphisms (SNPs) associated with treatment response. Whole-exome sequencing was also performed for 74 CZP extreme responders and non-responders within the same population, as well as 1546 population controls. No common SNPs or rare functional variants were significantly associated with CZP response, though a non-significant enrichment in the RA-implicated *KCNK5* gene was observed. Two SNPs near spondin-1 and semaphorin-4G approached genome-wide significance. The results of the current study did not provide an unambiguous predictor of CZP response.

## Introduction

Rheumatoid arthritis is a chronic, systemic autoimmune disease of unknown etiology, affecting between 0.3 and 1% of the global population. It affects the joints, connective tissues, muscle, tendons, and fibrous tissue leading to reduced quality of life, disability, and early mortality for sufferers [1].

Established treatment approaches, focused on sequential monotherapies and step-up combination therapies, have underserved many patients. Recently, it has become clear that addressing the underlying inflammatory processes early and prompt disease treatment with biological disease-modifying anti-rheumatic drugs (bDMARDs) such as TNF inhibitors (TNFi), is more successful in terms of limiting radiological progression and minimising loss of mobility and function [2]. However, whilst targeting TNF in RA patients represents a significant advance in treatment options, approximately 30–40% of treatment-naïve patients in clinical trials do not respond adequately to current drug treatment [3–5]. Although these reagents are highly beneficial in the majority of patients, their relatively high cost and potential safety liabilities are problematic in prescribing for broad patient populations.

Several factors have been identified in susceptibility to RA, including 2 to 3-fold increased risk in women and 1.3 to 2.4-fold higher risk among smokers. An increase in risk is also seen in individuals positive for anti-citrullinated peptide antibodies [6]. Despite extensive efforts, there are currently no accepted molecular or genetic biomarkers qualified for use in RA diagnosis and therapy. Therefore, current treatment paradigms operate without guarantee of patient benefit.

Until recently, the search for genetic markers associated with RA and anti-TNF response has focused on genes involved in RA susceptibility and disease pathways [7, 8] and genes involved in TNF production and signalling [9]. Over the last decade, a pool of DNA variants proposed as predictors of response to anti-TNFs has been uncovered following pharmacogenetic analyses of RA cohorts [10]. For example, the common *TNF* single nucleotide polymorphism (SNP) -308G>A (rs1800629), proposed to be associated with RA treatment outcome [11], has been rigorously investigated with often conflicting results [12–14].

More recently, genome-wide array and sequencing approaches have afforded researchers increased scope to search in an unbiased manner for clinically relevant biomarkers associated with RA and response to TNF blockers. However, the first round of these analyses, focused on European or Caucasian patients undergoing therapy with etanercept, infliximab and adalimumab, have generated further ambiguous and conflicting findings [10, 15–18]. The largest genome-wide association study to date, consisting of 2706 patients from 13 European cohorts, including those which had previously indicated SNP associations with RA, showed no association in a meta-analysis [18], possibly due to data or population heterogeneity.

However, eight candidate loci did show replication in Dutch patients from the DREAM cohort (n = 882), with consistent suggestive associations in two further cohorts in a subsequent meta-analysis [10]. Although none of these markers reached genome-wide significance, the directionality of association for three SNPs (rs1568885, rs1813443 and rs4411591) was consistent in all four analyses and may merit further study [10].

Subsequent genome-wide studies and meta-analyses have had limited success at replicating most candidate SNPs [19–21], however associations with *GFRA1*, *MED15*, *PTPRC*, and the *PDE3A*-*SLCO1C1* region have been successfully replicated in at least one independent cohort [22–25]. Recent evidence suggests that some these SNP associations may be drug-specific [24], which could explain the inability of some studies to replicate them. Integrated analysis of genomic and transcriptomic datasets has confirmed the therapy-specific nature of TNFi response [26]. In addition, substantial phenotypic heterogeneity between RA cohorts may make replication of TNFi SNP associations more difficult. Therefore, machine learning approaches capable of abstracting clinical and genetic predictors have been developed in an effort to classify patients according to their likelihood of responding to treatments [27, 28]. Despite the relatively high heritability of TNFi response [29], inclusion of SNPs previously associated with TNFi response provides limited improvement in the accuracy of machine learning models [27].

To date, no GWAS has been reported for certolizumab pegol. We report the first genome-wide study of a North American cohort of RA patients with moderate to severe RA, with the objective of discovering DNA variants associated with CZP response.

## Materials and methods

### Patients

The study protocol was approved by the Institutional Review Boards at Duke and Columbia University and written informed consent for participation was obtained at study entry for all subjects. North American patients (≥18 years) were selected from the REALISTIC phase IIIb study (NCT00717236). All participants were diagnosed with adult onset RA as defined by 1987 American College of Rheumatology (ACR) criteria for at least 3 months. In addition, they had either not tolerated or responded unsatisfactorily to at least one DMARD prior to study entry [30]. Patients were assessed using a 28 joint count and were defined as having active disease if they had at least five tender and at least four swollen joints, C-reactive protein (CRP) ≥10 mg/l and/or ≥ 28mm/hour erythrocyte sedimentation rate (ESR) and had moderate to severe RA at study entry [30] (Table 1).

**Table 1:** Characteristics of study sample for GWAS.

**Patient demographics**	
Self-reported gender, % female	79.5
Age, mean (S.D) years	56+/-12
Duration, years	
Mean (S.D.)	9.1+/-9.0
Median (interquartile range)	6.0 (2.3–13.6)
Disease duration <2 years, *n* (%)	66 (21.8)
Tender joint count, mean (S.D.)	15.4+/-6.8
DAS 28 (ESR), mean (S.D.)	6.4+/-1.0
ACR20 at week 6, *n* (%)	138 (45.7)
ACR20 at week 12, *n* (%)	146 (48.3)
ACR70 at week 6, *n* (%)	17 (5.6)
ACR70 at week 12, *n* (%)	34 (11.3)
Self-reported ethnicity, *n (%)*	
Caucasian	293 (97.0)
CRP, mg/l; Median (interquartile range)	9.0 (5.0–18.0)
ESR, mm/h: Median (interquartile range)	36.0 (25.0–51.0)
Anti-CCP positive at baseline, *n* (%)	180 (66.4)
RF positive at baseline, *n* (%)	208 (74.8)
**Treatment history**	
Previous TNF inhibitor use, *n* (%)	142 (47.0)
Other Medication at baseline, *n* (%)	
Methotrexate	216 (71.5)
Steroids	186 (61.6)
Statins	68 (22.5)
Lefluonamide	21 (7.0)
Azathioprine	1 (0.03)

ACR20, 20% improvement in ACR score; ACR70, 70% improvement in ACR score; DAS, Disease Activity Score; CCP, cyclic citrullinated peptide; RF, rheumatoid factor.

### GWAS of common SNPs

#### Genotyping

A total of 2,372,361 SNPs in 413 patients were genotyped using the Illumina Omni2.5M array platform. SNP genotypes were called using the Illumina GenomeStudio Software package Version 2011.1 with genotyping module version 1.9.4 according to the manufacturer’s instructions.

*Quality control (QC)*. A series of QC checks were carried out to ensure sample integrity. Biological sex was assessed for concordance between the genetically-inferred and self-reported gender. Duplicate samples and cryptic relatedness were identified based on genetic data using PLINK [31]. Samples found to be related at the level of first cousins or closer (pairwise estimate of identity-by-descent exceeding 0.125) were considered excessively related and one sample from each pair was removed at random. Quantitative estimates of ancestry (principal components analysis (PCA) implemented in EIGENSTRAT software [32]) were performed using LD pruned SNPs to estimate genomic ancestry and compared with self-reported ethnicity.

Data quality was also evaluated at the marker level to remove low quality genotypes. Two subjects missing >10% of genotype calls and one subject with an anomalously low inbreeding coefficient (F statistic outside of 6 standard deviations from the mean) were excluded. A total of 8629 poorly performing markers missing >10% of genotype calls and 3144 heterozygous haploid genotypes were removed. In addition, rare variants with a minor allele frequency (MAF) of <1% were excluded. No SNPs were found to deviate from Hardy-Weinberg equilibrium when a Bonferonni-adjusted threshold of 2.91x10^-8^ was applied.

Following QC, data were available for 1,685,541 SNPs in 360 individuals of primarily European ancestry. Of these, 302 individuals treated with CZP were included in the statistical analysis. Disease activity Score (DAS28 ESR) data were not available for all individuals (Table 2) and where data was missing for individual outcome measures, those patients were excluded from subsequent analysis. Only a minor difference in ACR20 response (45.7% vs 48.3%, Table 1) was observed between weeks 6 and 12, therefore only week 6 (ACR20_W6) was used for the analysis.

**Table 2:** Summary of data available for individual clinical outcome measures^a^.

	ACR20_W6	RD28_W6	ΔDAS28_W6	ΔDAS28_W12
**Total**	362	347	344	339
**CZP**	302	287	287	284
**Placebo**	60	60	57	55

ACR20_W6, 20% reduction in ACR at week 6; RD28_W6, reduction in DAS28 at week 6 (dichotomous phenotype); ΔDAS28_W6, change in DAS28 at week 6; ΔDAS28_W12, change in DAS28 at week 12.

^a^Incomplete outcome data available for DAS28.

#### Imputation

After QC, non-ambiguous SNPs, including rare variants with MAF<1%, were used as input for imputation using haplotypes from the 1000 Genomes phase1 integrated reference panel. Prephasing of haplotypes was performed using SHAPEIT software [33]. Imputation of missing and un-genotyped SNPs was then performed on 5Mb segments from each autosome and chromosome X using IMPUTE2 [34] according to recommended best practices. High confidence SNPs with greater than 90% imputation confidence were then merged using GTOOL and converted back to PLINK format for association analysis.

#### Statistical analysis

Four primary outcomes were tested for association within the study: ACR20 and decrease in DAS28 ESR >1.2 at week 6 (both as dichotomous variables), and change in DAS28 ESR at week 6 and 12 (continuous variables). Testing for association between individual outcome measures and each SNP were carried out using logistic or linear regression, after correcting for gender and genetic ancestry using the top 3 principal component axes with significant Tracy-Widom statistics, explaining 2.78% of the observed variance.

#### Analysis of candidate SNPs

Using a targeted approach harnessing known GWAS hits in autoimmune disease and genes involved in the TNF pathway, SNPs with higher prior probability of association with CZP response were tested for enrichment of association beyond that expected under the null hypothesis. Of 1618 SNPs previously reported to be associated with auto-immune diseases (SNPs with p< 5x10^-8^ obtained by searching GWAS Central for the MeSH term “autoimmune disease”), 72 were specifically associated with RA. These SNPs have a higher probability of association with the CZP outcomes than SNPs selected randomly. A total of 1403 out of 1618 SNPs were present on the Omni2.5M chip or were available in the imputed dataset. Of the 72 RA-associated SNPs, 64 were available for testing. To examine the role of genetic variation in genes involved in TNF signaling, a further hypothesis driven approach was carried out using 13303 common SNPs located within 112 genes that were annotated as participating in TNF signaling (hsa04668) according to KEGG pathway ontologies [35]. Significance scores for the subset of 13303 TNF-signaling SNPs were compared against a uniform distribution of p-values that would be expected under the null hypothesis using quantile-quantile plots. A review of the literature was used to identify 94 SNPs previously reported to be associated with TNF inhibitor response. Of these, 86 SNPs were present in the Omni2.5M array or imputed dataset.

### Whole-exome sequencing

#### Patient selection

Extreme non-responders (hereafter referred to as “non-responders”) were defined as ACR20 failures at week 6 and 12, with maximum reduction in DAS 28 ESR at week 12 compared with baseline (BL) of 0.33 and maximum reduction in DAS 28 ESR at week 6 compared with BL of 0.6, to eliminate secondary loss of response. Extreme responders (hereafter referred to as “responders”) were defined as individuals with a positive ACR70 score at 12 weeks. Using these criteria, a total of 81 non-responders and 40 responders were identified (summary shown in Table 3).

**Table 3:** Characteristics of study sample for whole exome sequencing.

Patient demographics	Responders	Non-responders
Self-reported gender, % female	77.5	80.2
Age, mean (S.D) years	54.5+/-15	54.7+/-12
Duration, years		
Mean (S.D.)	8.9+/-10.4	7.5+/-7.8
Median (interquartile range)	4.7 (1.5–13.3)	4.0 (1.7–11.0)
Disease duration <2 years, *n* (%)	13 (32.5)	21 (25.9)
Tender joint count, mean (S.D.)	14.4+/-7.0	15.7+/-7.0
DAS 28 (ESR), mean (S.D.)	6.3+/-1.0	6.3+/-1.1
ACR20 at week 6, *n* (%)	34 (85.0)	0 (0.0)
ACR20 at week 12, *n* (%)	40 (100)	0 (0.0)
ACR70 at week 6, *n* (%)	12 (30.0)	0 (0.0)
ACR70 at week 12, *n* (%)	40 (100)	0 (0.0)
Self-reported ethnicity, *n* (%)		
Caucasian	30 (75)	75 (93)
CRP, mg/l; Median (interquartile range)	9 (3.8–21.3)	7 (4.0–16.0)
ESR, mm/h: Median (interquartile range)	42.0 (26.3–57.8)	38.0 (27.0–52.0)
Anti-CCP positive at baseline, *n* (%)^a^	24 (66.6)	45 (61.6)
RF positive at baseline, *n* (%)^a^	28 (75.7)	57 (76.0)
**Treatment history**		
Previous TNF inhibitor use, *n* (%)^a^	16 (40.0)	37 (45.6)
Other Medication at baseline, *n* (%)		
Methotrexate	32 (80.0)	59 (72.8)
Steroids	26 (65.0)	47 (58.0)
Statins	7 (17.5)	20 (24.7)
Lefluonamide	0 (0.0)	3 (3.7)
Azathioprine	0 (0.0)	0 (0.0)

^a^Incomplete clinical data.

#### Sequencing and quality control

Whole-exome sequencing (WES) was performed using the Roche Nimblegen Human Exon v2 capture kit and KAPA library prep kit according to the manufacturer’s instructions. Sequencing was performed using the 100bp paired-end read protocol on the Illumina HiSeq 2000 instrument. After quality filtering the raw sequence data using CASAVA (Illumina, Inc., San Diego, CA, USA), adapter sequences were trimmed and the sequencing reads were aligned to the reference human genome (NCBI37/hg19) using BWA software [36]. Duplicate reads were then removed from the dataset using Picard (Broad Institute, Boston, MA, USA). Variant calling was performed using GATK [37] with local re-alignment around insertion/deletion variants (indels) and base quality recalibration for single-nucleotide variants (SNVs) [38]. Similarly sequenced control samples available at the Duke Center for Human Genome Variation (CHGV) served as the comparison group for a subset of the exome sequencing analyses.

#### Assesment of population structure

**Fig 1:**
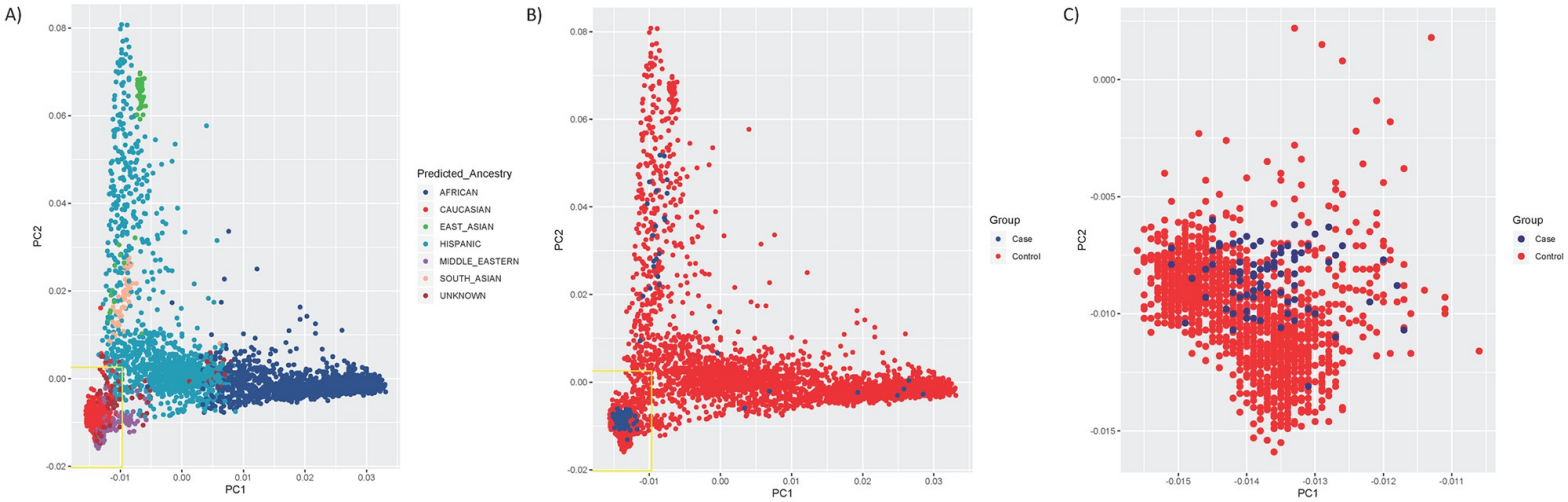
Scatterplot of Principal Component 1 (PC1) vs Principal Component 2 (PC2) from EIGENSTRAT ancestry analysis. (A) Scatter plot of PCs 1 & 2 prior to ancestry pruning, with subjects colored according to their predicted genetic ancestry. Yellow box indicates the region encompassing 6 s.d on PC1 and PC2. (B) Scatter plot of PCs 1 & 2 prior to ancestry pruning, with subjects colored by case/control status. Yellow box indicates the region encompassing 6 s.d on PC1 and PC2. (C) Scatter plot of PCs 1 & 2 after ancestry pruning and PCA outlier removal.

As the majority of subjects were self-described Caucasians, we sought to limit our analyses to that ancestry group in order to minimize the influence of population stratification between cases and controls. Preliminary ancestry predictions were perfomed during bioinfomatic processing using a panel of 12840 high coverage SNPs, based on principal components generated by EIGENSTRAT for cases, controls, and set of 4289 samples with pre-evaluated genetic ancestries. All cases and controls were initially assigned to one of six geographic ancestry groups (Caucasian, African, Hispanic, East Asian, South Asian or Middle Eastern) using a multinomial logistic regression model which provided a probability estimate of a sample belonging to each of the six groups. After restricting the cases and control samples to those predicted to be of Caucasian ancestry, all samples were further required to have EIGENSTRAT PC scores within 6 standard deviations of the mean on PCs 1 and 2. From an initial 121 CZP-treated patients and 2654 CHGV controls, ancestry pruning resulted in 74 CZP-treated patients (19 responders, 55 non-responders) and 1546 controls of European ancestry (Fig 1A and 1B). After removal of ancestry outliers, no major differences in population structure were observed between case-control groups (Fig 1C). Therefore no additional adjustment for genetic ancestry was included in the WES association analysis. The first two principal components accounted for 0.3% of the observed variance. Study characteristics after ancestry pruning are shown in S1 Table.

#### Variant quality filtering and association testing

Variants were filtered to include only those located in CCDS genes, with a GATK variant quality score in the 99.9 percentile and in the following functional categories: stop-gained, stop-lost, nonsynonymous coding, essential splice site, and coding region indels. Genotypes with ≤3 reads were considered missing for any individual. Variants were excluded if they were missing in ≥10% of cases or controls, or if they showed significant deviation from Hardy-Weinberg equilibrium in controls. In addition, 6044 variants previously identified as being artifacts based on comparison of CHGV controls with publically available data from the Exome Variant Server (EVS; http://evs.gs.washington.edu/EVS/) were removed from the dataset.

Individual variants were tested for association using Fisher’s exact test. In addition, gene-based collapsing tests were performed following the method of Li and Leal [39], with adjustment for differences in coverage at the exon level between cases and controls. Variants qualified for inclusion in these tests if they had a minor allele frequency (MAF) <1% in both the study sample (combined cases and controls) and EVS, and fell into the functional categories above.

There were three major comparisons in the study: responders vs non-responders, responders vs controls + non-responders, and non-responders vs controls + responders. The within-cohort comparison of responders and non-responders benefits from the clarity of the two alternative phenotypes; however, since the patients in these groups were selected from the extremes of the outcome distribution, it is reasonable to also compare each extreme to a population control sample, as the misclassification rate in controls is unlikely to be high and the sample sizes available are much larger. Statistical significance was assessed using Bonferroni correction for the number of tested variants or genes (in the collapsing analysis).

## Results

We have carried out a two-stage genetic analysis seeking to identify DNA variants associated with response to CZP in a North American cohort with moderate to severe RA.

### Patient population

The patient population was derived from the REALISTIC phase IIIb study [30] and the subjects used for the GWAS are summarized in Table 1. 79.5% of participants in the study were women, with average age at entry of 56 years. Mean duration of disease was 9.1 years with 22% of the population having disease duration of <2 years. Median CRP level at entry was 9.0 mg/l and median ESR was 36.0 mm/h. Mean DAS 28 ESR at entry was 6.4. 60% of the population for which data were available was positive for anti-citrullinated peptide antibodies and 69% positive for rheumatoid factor. Previous exposure to anti-TNF agents had occurred in 47% of patients, while 72% were on methotrexate, 62% on steroids and 23% on statins at baseline. A small number of patients were receiving other DMARDs on study entry (lefluonamide n = 21; azathioprine n = 1).

### GWAS

**Fig 2:**
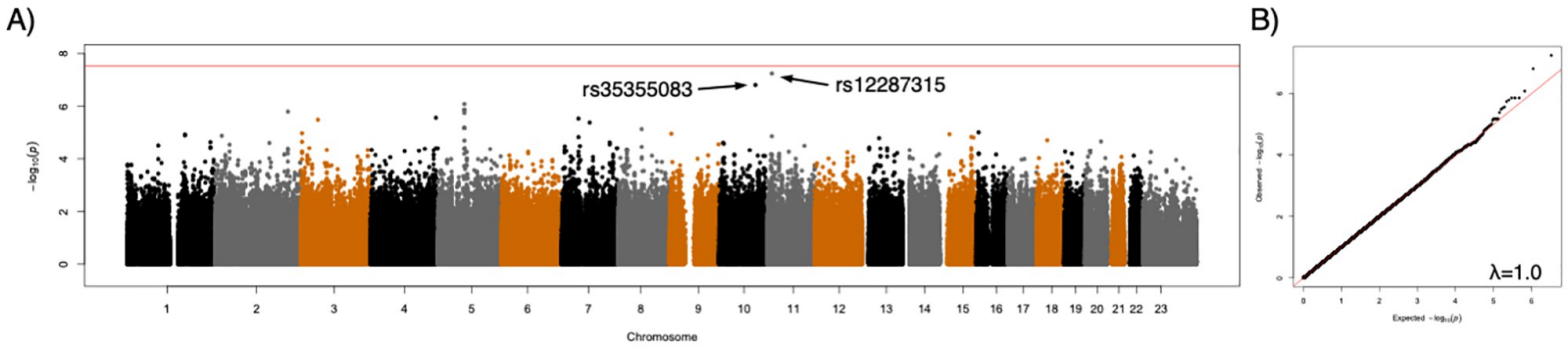
Results of genome-wide association study of certolizumab pegol response, as measured by DAS28 (week 12) scores. (A) Manhattan plot of significance scores from linear regression analysis. (B) Quantile-quantile plot of observed significance scores vs expected under the null hypothesis. The red line indicates the Bonferroni-adjusted threshold for statistical significance.

**Fig 3:**
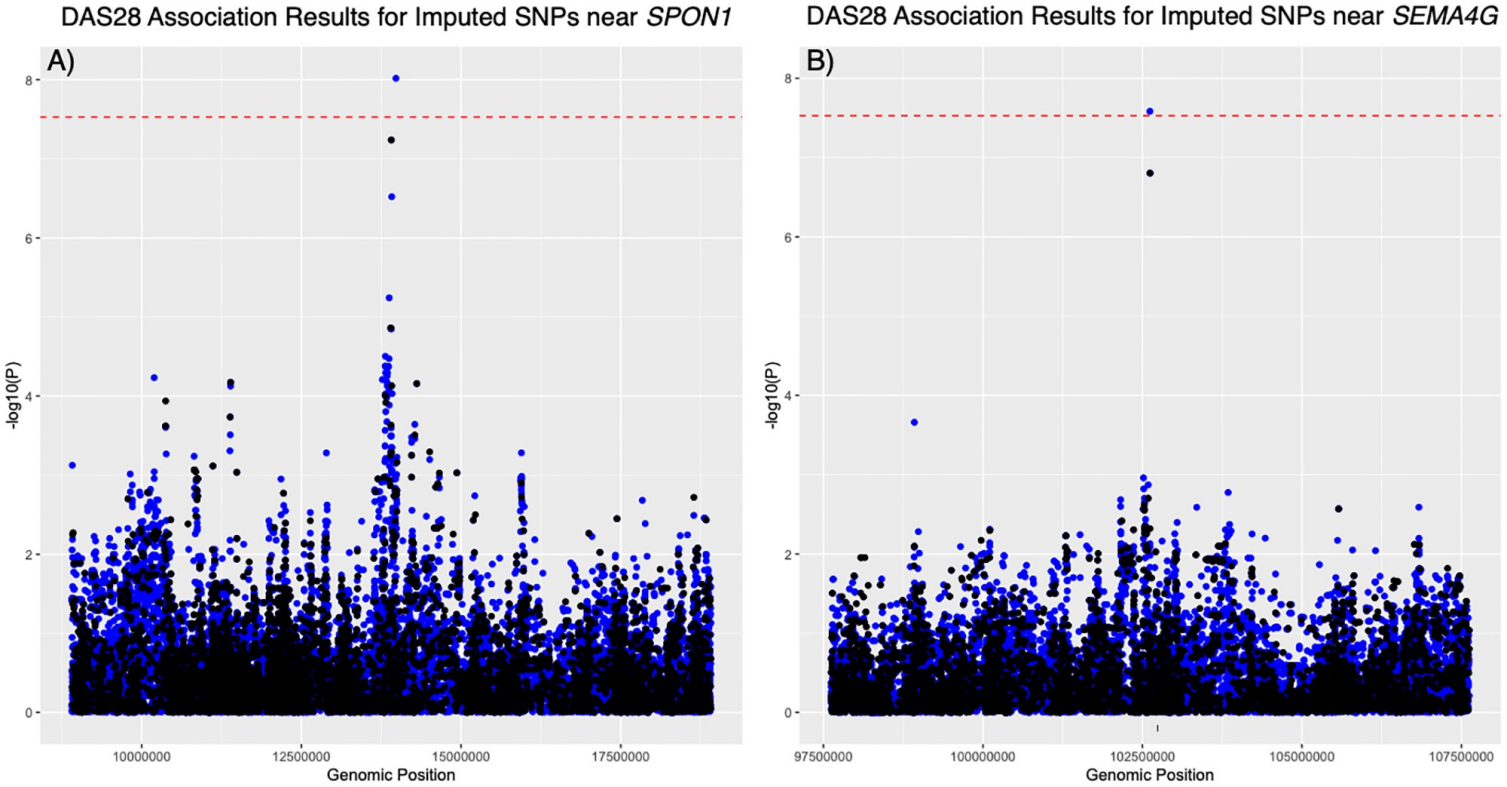
Fine mapping of regions with suggestive associations with DAS28 (week 12). (A) Manhattan plot of significance scores from linear regression analysis of DAS28 for common SNPs in a 10Mb window around the *SPON1* locus. (B) Manhattan plot of significance scores from linear regression analysis of DAS28 for common SNPs in a 10Mb window around the *SEMA4G* locus. SNPs genotyped on the Omni2.5M array are shown in black while imputed SNPs appear in blue. The dotted red line indicates the original Bonferroni threshold for significance.

After Bonferroni correction for multiple testing, using categorical or continuous outcome variables (ACR20 and DAS28), we were unable to demonstrate genome-wide significant associations between any genotyped SNP and therapeutic response to CZP for any of the outcome measures. For three of the measures, ACR20 and DAS28 ESR reduction of >1.2 at week 6 (dichotomous variables), and change in DAS28 ESR at week 6 (a continuous variable), there was no evidence of SNPs associated with outcome (S1 Fig). For the change in DAS28 ESR at week 12 outcome measure, two SNPs approaching genome-wide significance were observed (rs12287315 and rs35355083) (Fig 2). Neither of these SNPs were located within genes; rs12287315 (p = 5.78x10^-8^; β = -1.08) is located 76 kb upstream of the spondin-1 (*SPON1*) gene and rs35355083 (p = 1.57x10^-7^; β = -1.14) is located in an intergenic region 110 kb upstream of the semaphorin 4G (*SEMA4G*) gene. Imputation of ungenotyped SNPs in a 10Mb window around each lead SNP identified two additional SNPs in these regions with stronger associations with DAS28 ESR at week 12 (Fig 3). These included rs78675205 (p = 2.61x10^-8^; β = -1.21) on chromosome 10, located downstream of the Paired Box 2 (*PAX2*) gene, and rs72873110 (p = 9.61x10^-9^; β = -1.13) located on chromosome 11, approximately 3kb upstream of the *SPON1* coding region. While the significance scores for these two variants exceeded the original threshold for statistical significance, they were not statistically significant when the association analysis was performed using all common SNPs from a genome-wide imputation and no additional associations were detected outside of these two regions (S2 Fig).

### Candidate SNP and pathway analysis

**Fig 4:**
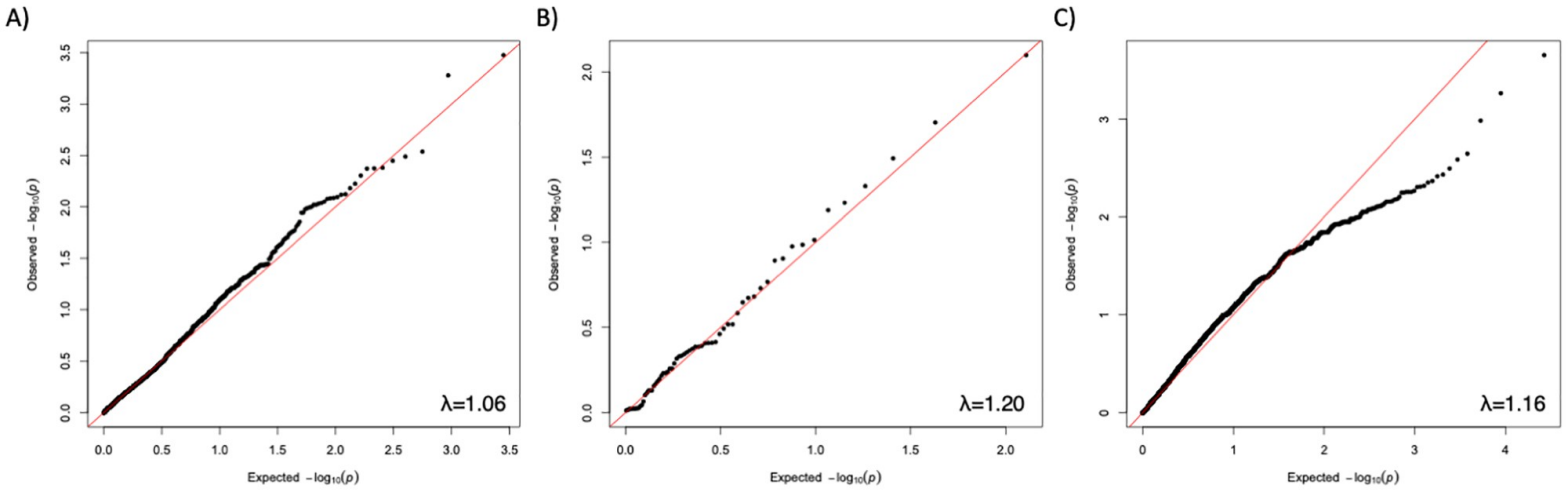
Quantile-quantile (QQ) plots of results from candidate SNP analysis. Plot of ACR20 significance scores from (A) 1403 SNPs previously reported to be associated with autoimmune disease, (B) 64 SNPs previously associated with rheumatoid arthritis, and (C) 13303 SNPs occurring in genes involved in TNF biological pathways.

We supplemented our genome-wide search for variants with a targeted analysis focused on SNPs with a higher prior probability of association, through previous documentation of association with auto-immune disease, as well as a sub-group of 64 SNPs associated specifically with RA. Similarly, we generated a candidate list of 13303 common variants observed in 112 genes reported to be involved in TNF biology [35]. No enrichment of association beyond that expected under the null hypothesis was observed using these approaches (Fig 4, S2–S4 Tables).

We further tested whether SNPs previously associated with response to TNF inhibitors showed evidence of association in the current study. As shown in Table 4, the previously reported associations with TNF inhibitor response were not observed in our cohort.

**Table 4:** SNPs previously associated with therapeutic response to TNF inhibitors.

SNP	PUBMEDID	Region	Nearest Gene	Genotyped/ Imputed	P-value (ACR20 Wk6)	P-value (DAS28 Wk6)	P-value (DAS28 Wk12)	P-value (RD28 Wk 6)
rs1800896	18615156	1q32.1	*IL19*	Genotyped	0.398	0.3498	0.6376	0.1547
rs1800629	18615156	6p21.33	*TNF*	Genotyped	0.4269	0.5255	0.3321	0.78
rs983332	18615156	1p22.3	*LMO4*	Genotyped	0.8091	0.7796	0.6857	0.2909
rs928655	18615156	1p22.2	*GBP6*	Genotyped	0.1043	0.1377	0.1266	0.1585
rs13393173	18615156	2q24.3	*CERS6*	Genotyped	0.5742	0.1443	0.1691	0.1655
rs437943	18615156	4p15.1	*ARAP2*	Genotyped	0.7274	0.2707	0.429	0.3062
rs10945919	18615156	6q26	*QKI*	Genotyped	0.579	0.1387	0.0937	0.2423
rs854555	18615156	7q21.3	*PON1*	Genotyped	0.5385	0.557	0.9665	0.9778
rs854548	18615156	7q21.3	*PPP1R9A*	Genotyped	0.2678	0.6907	0.5764	0.5743
rs854547	18615156	7q21.3	*PPP1R9A*	Genotyped	0.3115	0.6884	0.7034	0.6514
rs7046653	18615156	9p21.2	*MOB3B*	Genotyped	0.8241	0.4069	0.1583	0.3181
rs868856	18615156	9p21.2	*MOB3B*	Genotyped	0.8859	0.5538	0.1812	0.3844
rs774359	18615156	9p21.2	*C9ORF72*	Genotyped	0.8746	0.358	0.1237	0.4198
rs2814707	18615156	9p21.2	*MOB3B*	Genotyped	0.7717	0.4574	0.09011	0.3859
rs3849942	18615156	9p21.2	*C9ORF72*	Genotyped	0.8352	0.2486	0.06975	0.3508
rs6028945	18615156	20q12	*MAFB*	Genotyped	0.04575	0.3062	0.681	0.4589
rs6138150	18615156	20p11.21	*CST5*	Genotyped	0.7479	0.4045	0.6091	0.3948
rs6071980	18615156	20q12	*MAFB*	Genotyped	0.005448	0.2701	0.8796	0.157
rs2372536	20847201	2q35	*ATIC*	Imputed	0.4728	0.3225	0.8358	0.8671
rs1127354	20847201	20p13	*ITPA*	Imputed	0.8614	0.573	0.04869	0.4101
rs1801133	20847201	1p36.22	*MTHFR*	Genotyped	0.9395	0.8252	0.5648	0.4344
rs10919563	20847201	1q32.1	*PTPRC*	Imputed	0.6454	0.04959	0.1749	0.008771
rs12081765	21061259	1q23.3	*RXRG*	Genotyped	0.08769	0.4604	0.9424	0.3248
rs1532269	21061259	5p13.3	*PDZD2*	Imputed	0.06234	0.1323	0.6655	0.6793
rs17301249	21061259	6q23.2	*EYA4*	Imputed	0.2642	0.7368	0.8703	0.5782
rs7305646	21061259	12p12.3	*LMO3*	Genotyped	0.9131	0.7942	0.6261	0.8969
rs4694890	21061259	4p11	*TEC*	Genotyped	0.9198	0.7517	0.04464	0.3293
rs1350948	21061259	11p14.3	*CCDC179*	Genotyped	0.7382	0.907	0.6072	0.9468
rs4411591	23233654	18p11.31	*LOC100130480*	Imputed	0.9059	0.2898	0.1311	0.5541
rs7767069	23233654	6q12	*LOC102723883*	Imputed	0.1295	0.1288	0.7267	0.494
rs4651370	23233654	1q31.1	*PLA2G4A*	Imputed	0.1486	0.6554	0.6513	0.2614
rs1813443	23233654	11q22.1	*CNTN5*	Imputed	0.1955	0.01845	0.05309	0.06672
rs1447722	23233654	3q23	*CLSTN2*	Imputed	0.6619	0.2028	0.114	0.8809
rs1568885	23233654	7p21.3	*ETV1*	Imputed	0.5729	0.8817	0.8937	0.7815
rs12142623	23233654	1q31.1	*PLA2G4A*	Imputed	0.3175	0.6917	0.6671	0.4263
rs2378945	23233654	14q12	*NUBPL*	Imputed	0.1607	0.4346	0.7431	0.1113
rs10520789	22569225	15q26.2	*NR2F2*	Imputed	0.4158	0.5916	0.9036	0.3318
rs11870477	22569225	17q24.3	*KCNJ16*	Genotyped	0.6034	0.3105	0.9661	0.5014
rs16973982	22569225	15q26.2	*NR2F2*	Imputed	0.2349	0.8241	0.6655	0.6806
rs12001550	22569225	9q33.1	*TLR4*	Imputed	0.2199	0.6921	0.216	0.1803
rs885814	22569225	1p36.12	*ALPL*	Genotyped	0.4722	0.8331	0.39	0.6981
rs2293137	22569225	3p13	*FOXP1*	Imputed	0.9456	0.7823	0.6059	0.5943
rs885813	22569225	1p36.12	*ALPL*	Genotyped	0.2844	0.4211	0.6313	0.1756
rs1875620	22569225	9q22.1	*C9ORF47*	Genotyped	0.3423	0.2853	0.4925	0.7107
rs11525966	22569225	9q22.1	*C9ORF47*	Imputed	0.2175	0.3616	0.751	0.6859
rs960902	22569225	2p22.2	*QPCT*	Genotyped	0.6766	0.9054	0.4399	0.7923
rs1539909	22569225	18q22.3	*CBLN2*	Genotyped	0.6148	0.4174	0.8415	0.5087
rs11124586	22569225	2p22.2	*CDC42EP3*	Genotyped	0.7767	0.8684	0.747	0.2929
rs17679567	22569225	16q23.1	*CNTNAP4*	Genotyped	0.5491	0.6337	0.6196	0.9315
rs1827596	22569225	2q14.3	*CNTNAP5*	Imputed	0.9189	0.7095	0.1244	0.9499
rs4412918	22569225	15q21.3	*PRTG*	Imputed	0.2184	0.6313	0.6727	0.7144
rs17002731	22569225	4q21.1	*CXCL13*	Genotyped	0.9293	0.1418	0.8304	0.07673
rs1835353	22569225	2q14.3	*CNTNAP5*	Genotyped	0.5036	0.406	0.1828	0.4105
rs6427528	23555300	1q23.3	*CD84*	Imputed	0.7892	0.5482	0.2071	0.779
rs1503860	23555300	1q23.3	*CD84*	Imputed	0.8815	0.5997	0.1289	0.5712
rs12570744	23555300	10p14	*LINP1*	Genotyped	0.2621	0.4243	0.4004	0.08183
rs7141276	23555300	14q13.1	*SNX6*	Imputed	0.3357	0.4961	0.4497	0.2217
rs10833455	23555300	11p15.1	*NELL1*	Imputed	0.7821	0.3469	0.6566	0.8805
rs10833456	23555300	11p15.1	*NELL1*	Imputed	0.6108	0.255	0.719	0.9805
rs7932820	23555300	11p15.1	*NELL1*	Imputed	0.6176	0.3293	0.5869	0.8421
rs8009551	23555300	14q13.1	*SNX6*	Genotyped	0.2048	0.4228	0.4865	0.1568
rs4336372	23555300	5q35.2	*DRD1*	Genotyped	0.9886	0.8532	0.01123	0.8786
rs10265155	23555300	7q21.11	*MAGI2*	Genotyped	0.04158	0.04413	0.5201	0.3038
rs1990099	23555300	7q21.11	*MAGI2*	Imputed	0.2394	0.03951	0.6735	0.2659
rs284515	26776603	6q15	*MAP3K7*	Imputed	0.1955	0.8197	0.09406	0.4495
rs75908454	26776603	6q27	*WDR27*	Imputed	0.21	0.08461	0.891	0.8489
rs1679568	26776603	10q25.3	*GFRA1*	Imputed	0.5555	0.6671	1	0.9108
rs284511	26776603	6q15	*MAP3K7*	Imputed	0.8658	0.1713	0.8586	0.03014
rs6941263	25896534	6q21	*ARMC2*	Imputed	0.359	0.2057	0.3872	0.132
rs113878252	25896534	22q11.21	*MED15*	Imputed	0.1638	0.2092	0.9521	0.3039
rs6065221	25896534	20q12	*MAFB*	Genotyped	0.7959	0.3276	0.2482	0.5876
rs7195994	30166627	16q12.2	*FTO*	Imputed	0.9607	0.8829	0.7041	0.2118
rs10739537	30166627	9q33.1	*DBC1*	Imputed	0.4139	0.4443	0.6199	0.4562
rs948138	30166627	11q22.2	*MMP20*	Genotyped	0.3166	0.4068	0.7111	0.7946
rs11599217	30166627	10q26.2	*DOCK1*	Imputed	0.8675	0.8999	0.9294	0.9163
rs2187874	30166627	4p16.3	*ZNF595*	Imputed	0.1811	0.5109	0.5323	0.2259
rs150537045	30166627	8q23.3	*CSMD3*	Imputed	0.5835	0.4199	0.3298	0.7194
rs76668869	30166627	1p31.1	*ADGRL2*	Imputed	0.4255	0.164	0.7019	0.05351
rs2295463	30166627	14q13.2	*KIAA0391*	Imputed	0.4716	0.6512	0.3877	0.8045
rs34619498	30166627	4q24	*EMCN*	Imputed	0.2414	0.2441	0.2936	0.6553
rs140142800	30166627	10q21.2	*RHOBTB1*	Imputed	0.8841	0.9169	0.4225	0.664
rs78368496	30166627	11p15.1	*NAV2*	Imputed	0.3588	0.7284	0.4222	0.9777
rs147859879	30166627	5p15.33	*MRPL36*	Imputed	0.6686	0.3167	0.557	0.12
rs337527	30166627	9q22.33	*GABBR2*	Imputed	0.2747	0.441	0.1885	0.8569
rs11045392	22569225	12p12.2	*PDE3A*	Genotyped	0.5394	0.01316	0.06091	0.2323
rs3794271	22569225	12p12.2	*SLCO1C1*	Genotyped	0.4297	0.006361	0.04621	0.1448

### Exome sequencing analysis

In a complementary analysis, we screened for rare variants by WES of 19 extreme responders and 55 non-responders. Additionally, patients from these extremes of the response distribution were compared with a large sample of more than 1500 ethnically matched control samples sequenced in the same laboratory using identical data processing methods. S5 Table shows the sample size and the number of variants tested in each comparison.

**Fig 5:**
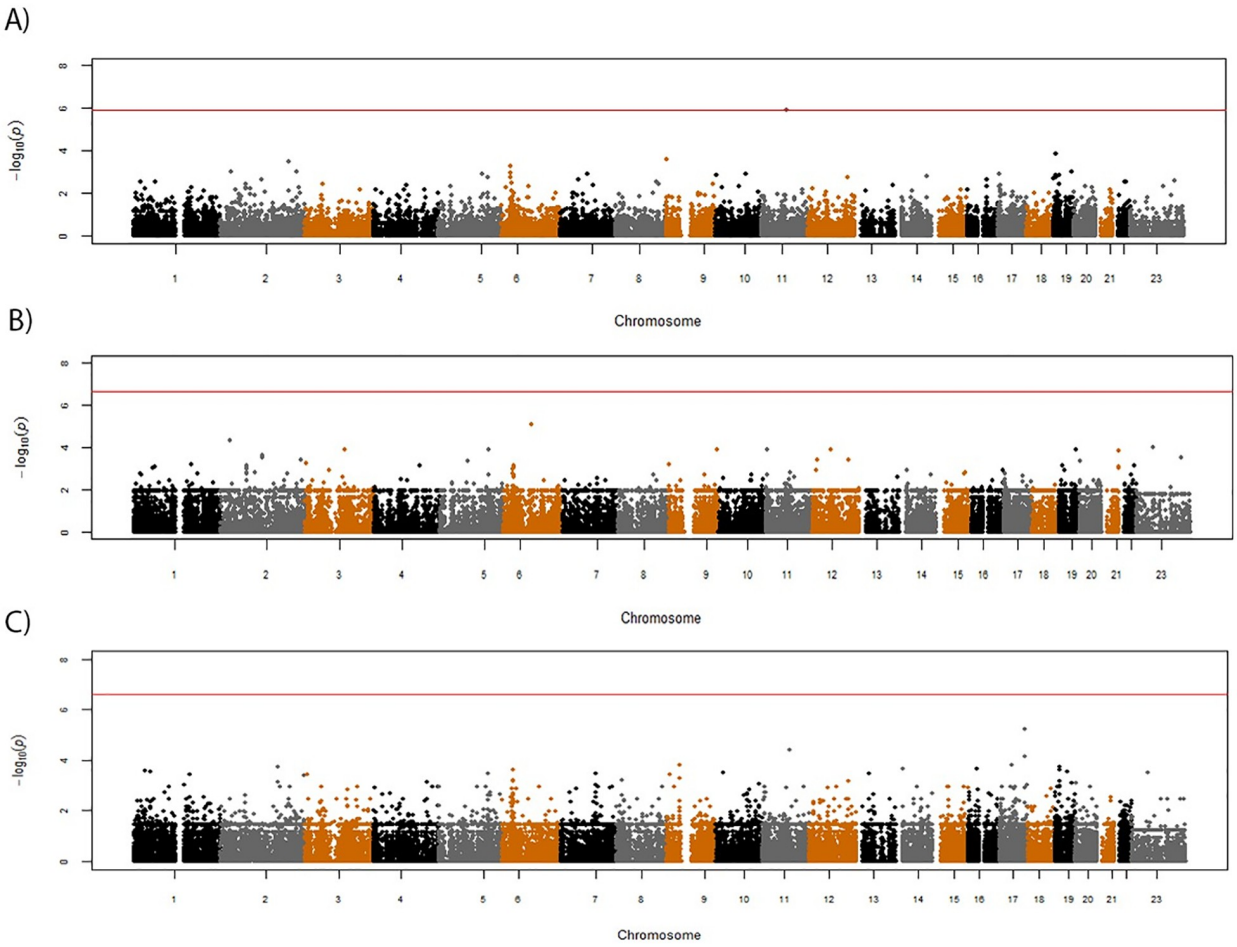
Manhattan plots of significance scores from single-variant analysis of exome sequencing data of patients selected from extremes of the CZP response distribution. In panel A, 19 CZP super-responders are compared to 55 non-responders. Panel B shows FET significance scores for 19 CZP super-responders compared to sequence data from a larger group of 1546 ethnically matched population controls and 55 non-responders, while panel C shows results of analysis of 55 CZP non-responders versus 1546 ethnically matched population controls and 19 super-responders. Horizontal red lines in figures A-C indicate the Bonferroni-corrected threshold for statistical significance.

**Fig 6:**
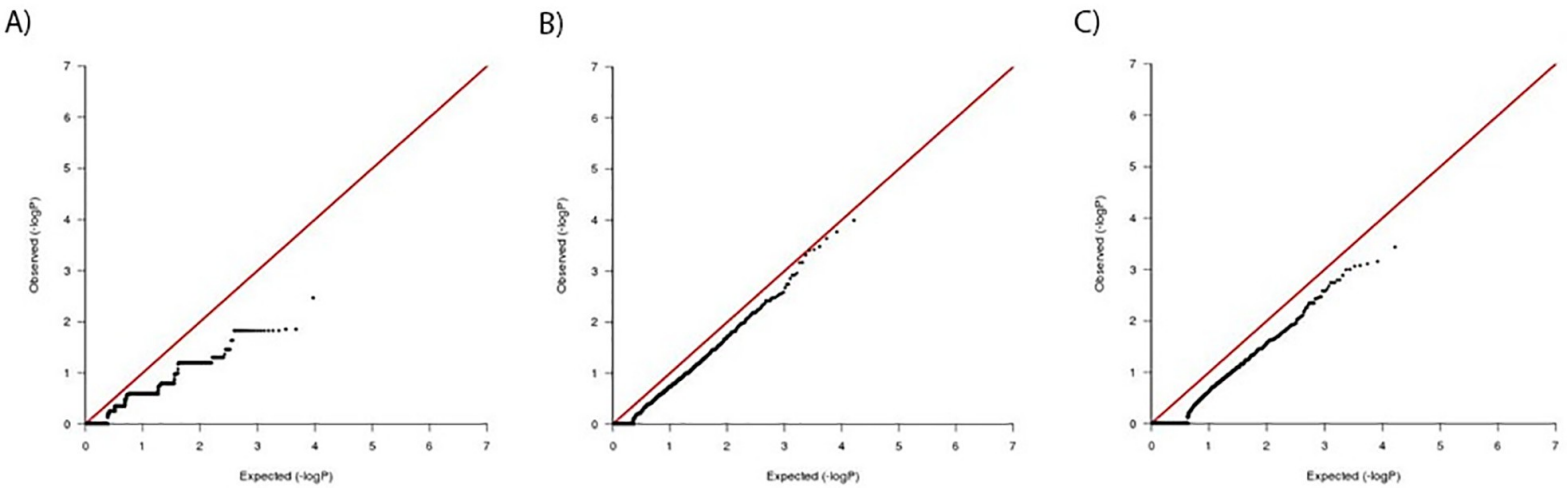
Results of gene-based collapsing analysis of CZP responder and non-responder exome sequencing data. (A) QQ plot of observed vs null FET significance values for CZP super-responders vs non-responders. (B) CZP super-responders are compared to sequence data from 1546 population controls and 55 non-responders, while C shows QQ plot of CZP non-responders compared to 1546 population controls and 19 super-responders. In all three analyses, no individual genes exceeded what was expected under the null hypothesis.

For all comparisons, the single-variant tests did not reveal any variant showing significant association with responder or non-responder status (Fig 5). The p-value distributions were non-uniform and tended to be less significant than expected under the null, probably owing to both the small sample sizes and the relatively low allele frequency distribution of the variants tested. For gene-based collapsing tests, the results were similar (Fig 6), with no individual gene showing significant association with therapeutic response after correcting for multiple testing. The top ten SNPs and genes most significantly associated with CZP response are shown in Tables 5 and 6.

**Table 5:** Top ten most strongly associated individual variants from whole exome sequencing.

	Variant	Gene	P-value
Responders vs. Non-responders + Controls	6_80228535_G	*LCA5*	8.541E-006
	2_26689619_A	*OTOF*	5.046E-005
	X_49082499_T	*CACNA1F*	1.03E-004
	3_113376202_C	*KIAA2018*	1.34E-004
	9_136305552_A	*ADAMTS13*	1.34E-004
	11_3661588_3661588_1bp_INS	*ART5*	1.346E-004
	12_53448166_T	*TENC1*	1.362E-004
	19_47910643_T	*MEIS3*	1.362E-004
	5_140237030_G	*PCDHA10*	1.365E-004
	21_47836571_T	*PCNT*	1.469E-004
Non-responders vs. Responders + Controls	17_73520485_C	*TSEN54*	6.133E-006
	11_76062206_C	*PRKRIR*	3.811E-005
	17_72240177_G	*TTYH2*	7.369E-005
	9_35663069_T	*ARHGEF39*	1.487E-004
	17_33690160_T	*SLFN11*	1.487E-004
	19_15587345_C	*PGLYRP*	1.852E-004
	2_167055393_C	*SCN9A*	1.877E-004
	16_24564879_24564882_4bp_DEL	*RBBP6*	2.213E-004
	14_20711681_A	*OR11H4*	2.256E-004
	6_32797809_T	*TAP2*	2.35E-004
Responders vs. Non-responders	11_71249125_71249145_21bp_INS	*KRTAP5-8*	1.257E-006
	19_7528734_G	*ARHGEF18*	1.362E-004
	9_712156_G	*KANK1*	2.466E-004
	2_196720705_C	*DNAH7*	3.06E-004
	6_25914853_A	*SLC17A2*	4.977E-004
	19_51530741_G	*KLK11*	9.068E-004
	2_220072431_A	*ZFAND2B*	9.082E-004
	2_32713706_T	*BIRC6*	9.897E-004
	6_26017542_C	*HIST1H1A*	0.0011
	10_88930249_T	*FAM35A*	0.0012

**Table 6:** Top ten most strongly associated genes in collapsing tests of multiple rare variants.

	Gene	P-value
Responders vs. Non-responders + Controls	*C11ORF93*	3.637E-004
	*CUL2*	6.964E-004
	*PPME1*	7.715E-004
	*EOGT*	8.394E-004
	*CROT*	8.583E-004
	*PIK3AP1*	9.998E-004
	*OR5AU1*	9.998E-004
	*SAMD10*	0.0013
	*CLRN3*	0.0016
	*KIAA1107*	0.0016
Non-responders vs. Responders + Controls	*KIAA1522*	1.01E-004
	*BTN1A1*	1.703E-004
	*POF1B*	2.297E-004
	*IFNA4*	3.292E-004
	*GGN*	3.786E-004
	*GREB1*	3.887E-004
	*TSEN54*	4.814E-004
	*EIF3B*	6.756E-004
	*ACTL9*	6.779E-004
	*OR11H4*	0.0011
Responders vs. Non-responders	*CROT*	0.0034
	*ADAMTS2*	0.014
	*RBMXL3*	0.014
	*ADAMTS16*	0.0149
	*CLRN3*	0.0149
	*CNGB1*	0.0149
	*DUPD1*	0.0149
	*FARSB*	0.0149
	*KCNK5*	0.0149
	*KDM3B*	0.0149

Interestingly, among the top ten most highly associated results in the gene-based analysis was the potassium channel, subfamily K, member 5 (*KCNK5)* gene, which is known to be a critical factor in T cell activation [40]. In addition, *KCNK5* expression has previously been reported to be strongly correlated with RA disease severity [41] and up-regulation of *KCNK5* expression was found to be predictive of treatment failure in RA patients receiving the anti-IL-6 therapy tocilizumab [41], which inhibits RA inflammation through a different biological pathway to that targeted by anti-TNF therapies. In our collapsing analysis, non-synonymous variants in the *KCNK5* gene were present in 16% (3/16) of CZP extreme responders, while no patients (0/55) from the CZP non-responder group were found to have any predicted functional variants in the *KCNK5* gene.

## Discussion

We have completed an exploratory pharmacogenetic analysis of a cohort of 302 RA patients of Western European descent treated with CZP. A two-stage analytical approach was employed, using high content beadchip genotyping analysis to examine the role of common human genetic variation, followed by a second stage consisting of WES of extreme responders and non-responders, to investigate the association of rare genetic variants with CZP treatment response.

In the first stage of the analysis examining common variation, we were unable to demonstrate robust associations with any of the clinical outcome measures. Two novel candidates (rs35355083 near *SEMA4G* and rs12287315 near *SPON1*), showed strong trends toward association with change in DAS28 ESR at week 12, but did not meet genome-wide statistical significance. These variants have not been associated with response to TNF inhibitors previously, however a low-frequency variant located in the *FAR1*-*SPON1* intergenic region was previously reported to be associated with Etanercept response [42]. Imputation of ungenotyped SNPs near these suggestive associations revealed two additional variants with stronger associations with DAS28 ESR at week 12. While the p-values for these associations exceeded the original threshold for statistical significance, they were not found to be significant when the association analysis was performed using all common variants from a genome-wide imputation.

Semaphorins are a diverse group of proteins involved in regulation of cell movement and migration via interaction with cognate plexin or neuropilin receptors [43]. Recent studies have indicated that they play a role in many aspects of the immune system, including innate immunity and cell trafficking [44]. In the mouse, SEMA4G is required for cerebellar development [45], however, a clear role in auto-immune disease has yet to emerge.

Spondin 1(SPON1) is an extracellular matrix protein involved in regulation of neuronal outgrowth and inhibition of angiogenesis. It has been shown to bind to the extracellular domain of amyloid precursor protein and impairs cleavage by the beta secretase BACE 1, reducing beta-amyloid production [46]. Its expression has been reported to be elevated in osteoarthritis lesions in both humans and rodents, and its activation of TGF-beta *in situ* may promote osteoarthritis pathogenesis [47].

The involvement of these genes in immune signaling, as well as the reported role of spondin 1 in cartilage homeostasis [47], suggests that these associations may be biologically relevant. However, their failure to show significant association with CZP response after correction for multiple testing should prompt caution with regard to interpretation of the findings. As with all such candidates, replication of these SNPs in additional cohorts and biological validation will be required.

Whilst the genotyping stage of the analysis was intended to screen for common variants associated with CZP response, for complex diseases such as RA, such approaches typically require large cohorts and/or large effect sizes. One particularly compelling reason to search for rare variants in the study of therapeutic response to drugs is that drug response may be correlated with the underlying genetic cause of disease, and it is expected that much of the risk for human disease will be found in the rare-variant spectrum [48]. To address the risk of overlooking low frequency variants that contribute to CZP response, we implemented a WES approach in the second stage of the study. This approach enables identification of rare variants with larger effect sizes, despite the small sample sizes available. In this case, sequencing focused on 74 patients from the REALISTIC trial with extreme responses to CZP treatment; including 55 non-responders and 19 super-responders. Neither the single-variant tests nor the gene-based collapsing method identified any gene or variant that was significantly associated with CZP response. A modest, but non-significant, enrichment of variation was identified in the *KCNK5* gene in our collapsing analysis. The previous implication of this gene in RA disease progression, as well as the specific observation that increases in *KCNK5* expression are correlated with the failure of antibody-based therapies targeting similar inflammatory pathways involved in RA disease pathology, may suggest that additional investigation of *KCNK5* as a modifier of RA therapies is merited.

With the caveat that *KCNK5* and the two SNPs in *SEMA4G* and *SPON1* may be worthy of follow up, we have failed to demonstrate the presence of any variants with a clinically robust association with response to the anti-TNF agent CZP. While the lack of statistically significant associations could result from the limited size of our study, this finding is also consistent with many previous candidate gene and genome-wide efforts to discover genetic determinants of response to anti-TNF agents. Researchers have either been unable to discover or replicate initial findings in clinically relevant cohorts, or the statistical performance of the candidates has not been sufficiently compelling to justify follow up or clinical implementation. A *post hoc* analysis of the estimated statistical power for the GWAS component of our study indicated it had over 80% power to detect common variants with a MAF of 25% and a relative risk of 2.5, but was only sufficiently powered to detect more rare variants with a MAF of 5% when the relative risk was over 5 (S3A Fig). Similarly, the WES portion of our study was likely to be underpowered to detect enrichments of rare variants with moderate effect sizes (RR<6.0; S3B Fig).

RA is a heterogeneous disease with complex genetic and environmental etiology. Although we have focused our analysis on a largely homogenous Caucasian cohort of 302 patients, the study population comprised patients with differing clinical characteristics at entry. We have accommodated variability in disease severity and duration, and use of other medications in the analysis but were not able to adjust for the variability in biology underpinning disease heterogeneity and response/non-response. In addition, the development of antibodies against TNF inhibitors has recently been recognized a significant contributor to treatment failure [49, 50]. Our study did not account for the induction of antibodies against CZP. It therefore remains a possibility that heritable biomarkers predictive of risk do exist for sub-populations within diverse RA populations such as this one, but that their penetrance is diluted to an extent whereby they are not detectable.

Further complexity is added by the routine use of composite outcome measures such as DAS28 and ACR20, the primary endpoints used in this clinical study. They are comprised of a mixture of objective physical measurements, laboratory biomarker data, and subjective patient assessments. Robust associations may be discernible with a more refined focus on a single objective endpoint, although it should be noted that this may be misleading in a mixed patient population.

The goal of personalizing RA therapy is to identify patients where the likelihood of a beneficial outcome is optimized and the risk of adverse events is reduced or eliminated. Many studies with conflicting and ambiguous outcomes have been published investigating the pharmacogenomics of etanercept, infliximab and adalimumab in RA populations, and to date there have been no predictive anti-TNF genomic biomarkers established for clinical use. This report, the first pharmacogenomic analysis of CZP response, is in accord with these earlier efforts, and has been unable to demonstrate compelling evidence for the existence of genomic biomarkers of value in targeting this anti-TNF to a responsive patient sub-population.

In diseases such as RA, where there are complex, polygenic contributions to pathology and response to therapeutic intervention, it remains a possibility that combinations of clinical, environmental, and molecular markers will hold more promise for managing therapy.

## Supporting information

S1 Fig Results of genome-wide association study of certolizumab pegol response. Manhattan and quantile-quantile plots of significance scores for association with (A) ACR20 week 6 response, (B) logistic regression of reduction in DAS28 ESR week 6, and (C) linear regression of DAS28 ESR at week 6. The red line indicates the Bonferroni-adjusted threshold for statistical significance. Genomic inflation factor (λ) for each analysis is indicated in the bottom right of quantile-quantile plots.
(TIF)

S2 Fig Results of genome-wide association study of certolizumab pegol response using fully imputed common SNPs. Manhattan and quantile-quantile plots of significance scores for association with (A) ACR20 week 6 response, (B) logistic regression of reduction in DAS28 ESR week 6, (C) linear regression of DAS28 ESR at week 6, and (D) linear regression of DAS28 ESR at week 12. The red line indicates the Bonferroni-adjusted threshold for statistical significance. Genomic inflation factor (λ) for each analysis is indicated in the bottom right of quantile-quantile plots.
(TIF)

S3 Fig Power calculations for GWAS and WES studies. Graphs of statistical power estimated across a range of minor allele frequencies and effect sizes for (A) the GWAS of common SNPs and ACR20 response, and (B) collapsing analysis of rare variants identified by WES for non-responders vs population controls and super-responders. For rare variant collapsing analysis, cMAF indicates the cumulative minor allele frequency summed across multiple rare variants. All power calculations were performed using CaTS.
(TIF)

S1 Table Characteristics of exome sequencing sample after ancestry pruning (N = 74). (DOCX)

S2 Table ACR20 associations for SNPs previously associated with autoimmune disease. (DOCX)

S3 Table ACR20 associations for SNPs previously associated with rheumatoid arthritis. (DOCX)

S4 Table Top 200 ACR20 associations for SNPs located in or near TNF pathway genes. (DOCX)

S5 Table Sample sizes and number of variants tested in WES analyses. (DOCX)
