# Supplementary figures and images for: A genome-wide screen for variants influencing certolizumab pegol response in a moderate to severe rheumatoid arthritis population
Source: PLoS One. 2022 Apr 12;17(4):e0261165. doi: 10.1371/journal.pone.0261165 (PMC9004786; doi:10.1371/journal.pone.0261165)

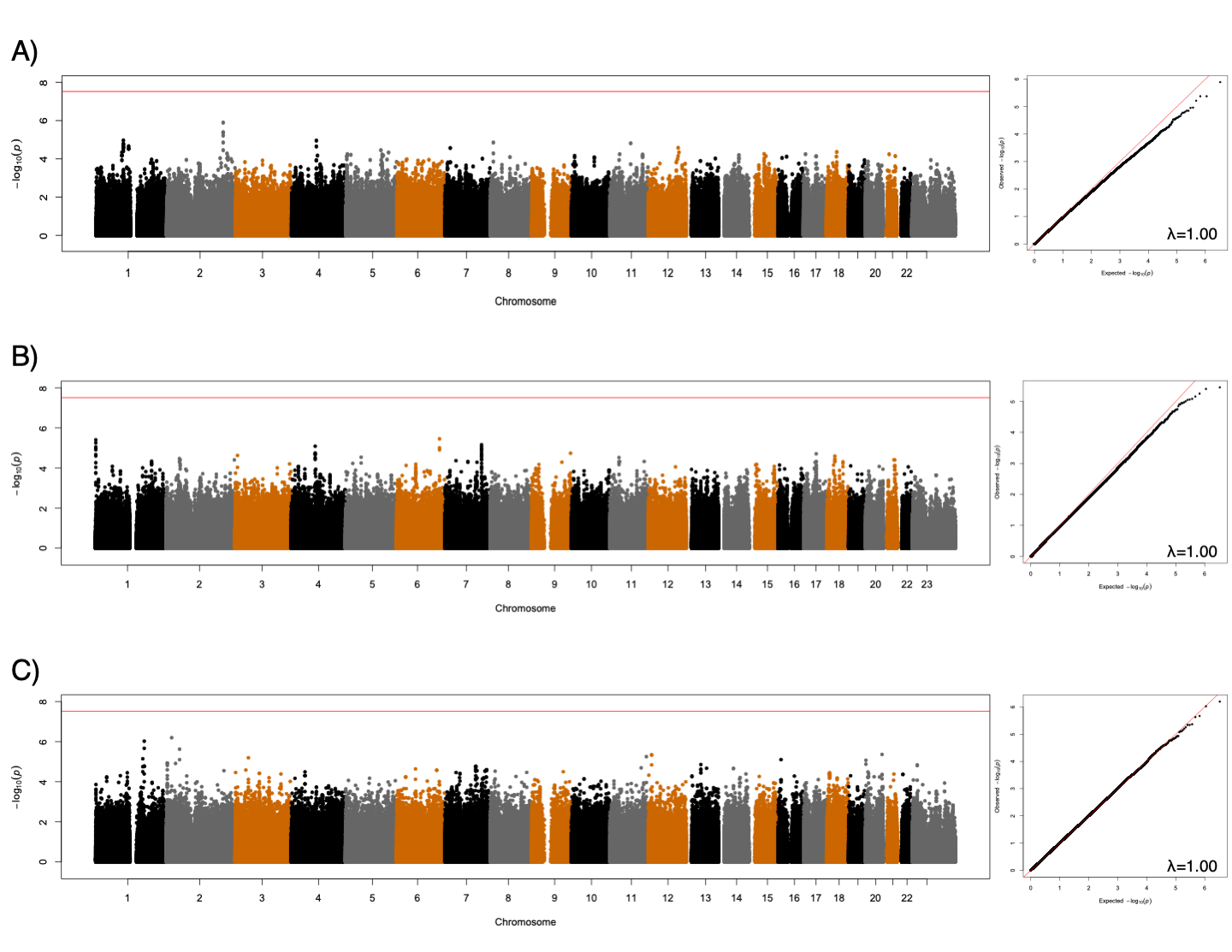

Supplement: S1 Fig — Manhattan and quantile-quantile plots of significance scores for association with (A) ACR20 week 6 response, (B) logistic regression of reduction in DAS28 ESR week 6, and (C) linear regression of DAS28 ESR at week 6. The red line indicates the Bonferroni-adjusted threshold for statistical significance. Genomic inflation factor (λ) for each analysis is indicated in the bottom right of quantile-quantile plots. (TIF) [file pone.0261165.s001.tif]

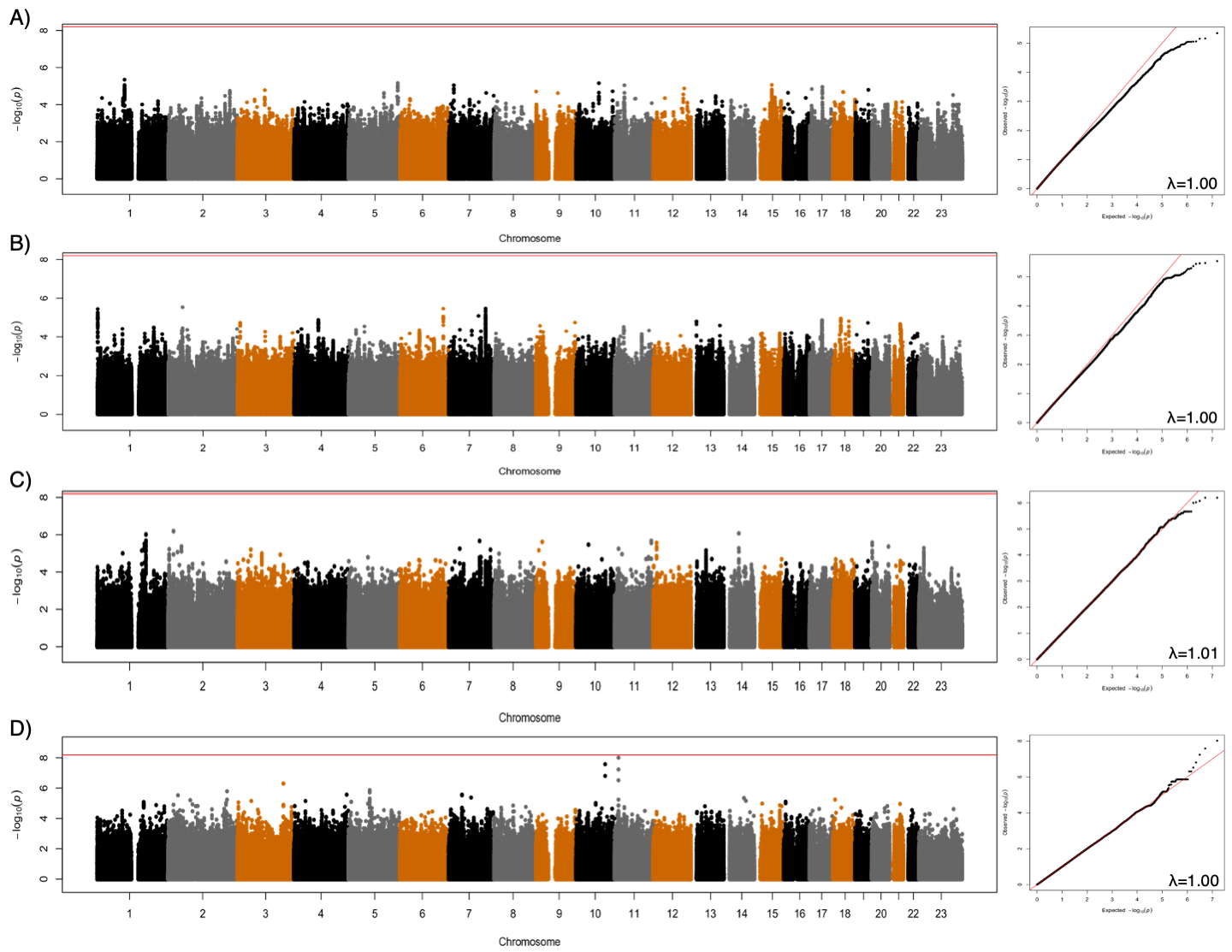

Supplement: S2 Fig — Manhattan and quantile-quantile plots of significance scores for association with (A) ACR20 week 6 response, (B) logistic regression of reduction in DAS28 ESR week 6, (C) linear regression of DAS28 ESR at week 6, and (D) linear regression of DAS28 ESR at week 12. The red line indicates the Bonferroni-adjusted threshold for statistical significance. Genomic inflation factor (λ) for each analysis is indicated in the bottom right of quantile-quantile plots. (TIF) [file pone.0261165.s002.tif]

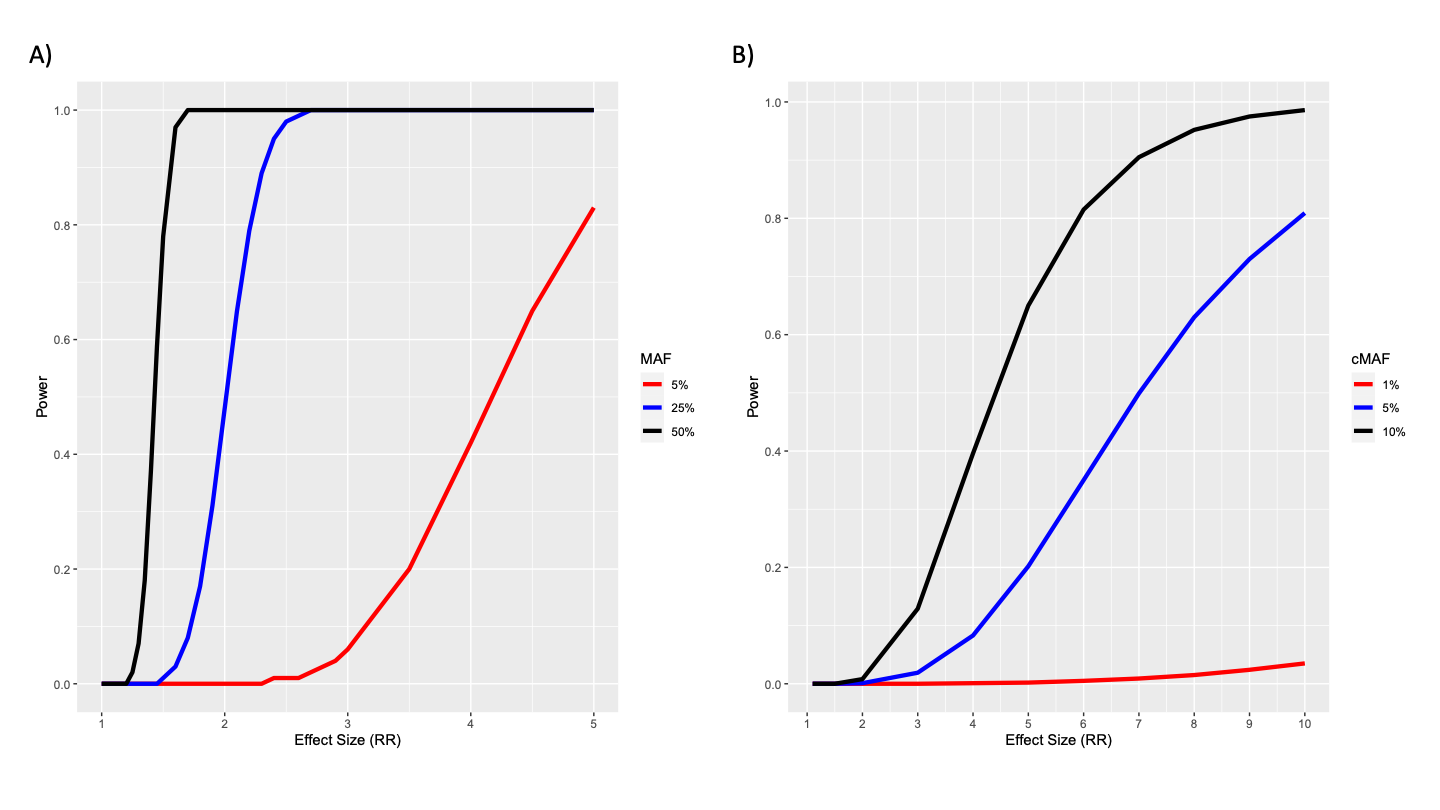

Supplement: S3 Fig — Graphs of statistical power estimated across a range of minor allele frequencies and effect sizes for (A) the GWAS of common SNPs and ACR20 response, and (B) collapsing analysis of rare variants identified by WES for non-responders vs population controls and super-responders. For rare variant collapsing analysis, cMAF indicates the cumulative minor allele frequency summed across multiple rare variants. All power calculations were performed using CaTS. (TIF) [file pone.0261165.s003.tif]
