# Supplementary material for: A genome-wide screen for variants influencing certolizumab pegol response in a moderate to severe rheumatoid arthritis population
Source: PLoS One. 2022 Apr 12;17(4):e0261165. doi: 10.1371/journal.pone.0261165 (PMC9004786; doi:10.1371/journal.pone.0261165)
Supplement: S1 Table — (DOCX) [file pone.0261165.s004.docx]

**Patient demographics Responders Non-responders**

Self-reported gender, % female 68.4 78.2

Age, mean (S.D) years 59.9+/-14 55.0+/-11

Duration, years

Mean (S.D.) 10.0+/-9.0 7.1+/-7.6

Median (interquartile range) 7.0 (2.4-14.0) 4.0 (1.5-10.4)

Disease duration <2 years, *n* (%) 4 (21.1) 17 (30.9)

Tender joint count, mean (S.D.) 15.2+/-7.2 16.0/-7.2

DAS 28 (ESR), mean (S.D.) 6.5+/-1.0 6.3+/-1.1

ACR20 at week 6, *n* (%) 15 (78.9) 0 (0.0)

ACR20 at week 12, *n* (%) 19 (100) 0 (0.0)

ACR70 at week 6, *n* (%) 2 (10.5) 0 (0.0)

ACR70 at week 12, *n* (%) 19 (100) 0 (0.0)

Self-reported ethnicity, *n* (%)

Caucasian 19 (100) 55 (100)

CRP, mg/l; Median (interquartile range) 9.0 (6.0-21.0) 7.0 (3.0-14.5)

ESR, mm/h: Median (interquartile range) 36.0 (21.0-72.5) 38.0 (25.5-53.5)

Anti-CCP positive at baseline, *n* (%) * 10 (55.6) 27 (56.3)

RF positive at baseline, *n* (%)* 14 (73.7) 37 (74.0)

**Treatment history**

Previous TNF inhibitor use, *n* (%) 8 (42.1) 21 (38.2)

Other Medication at baseline, *n* (%)

Methotrexate 16 (84.2) 37 (67.3)

Steroids 11 (57.9) 31 (56.4)

Statins 3 (15.8) 13 (23.6)

Lefluonamide 0 (0.0) 3 (5.5)

Azathioprine 0 (0.0) 0 (0.0)
