# Supplementary material for: A genome-wide screen for variants influencing certolizumab pegol response in a moderate to severe rheumatoid arthritis population
Source: PLoS One. 2022 Apr 12;17(4):e0261165. doi: 10.1371/journal.pone.0261165 (PMC9004786; doi:10.1371/journal.pone.0261165)
Supplement: S2 Table — (DOCX) [file pone.0261165.s005.docx]

| SNP | Chromosome | Position | identifier | Reported P-value | Dataset | P-value  (ACR20 at Wk 6) | OR |
| --- | --- | --- | --- | --- | --- | --- | --- |
| rs660895 | 6 | 32577380 | HGVM265114 | 1.00E-300 | Genotyped | 0.05792 | 0.7143 |
| rs6910071 | 6 | 32282854 | HGVM7231436 | 1.00E-299 | Genotyped | 0.9514 | 0.9888 |
| rs9268839 | 6 | 32428772 | HGVM8420016 | 1.00E-250 | Genotyped | 0.06663 | 1.379 |
| rs1770 | 6 | 32627833 | HGVM4383 | 2.00E-232 | Imputed | 0.07442 | 0.7316 |
| rs3129889 | 6 | 32413545 | HGVM1598142 | 1.00E-206 | Genotyped | 0.537 | 1.185 |
| rs689 | 11 | 2182224 | HGVM421 | 5.00E-196 | Imputed | 0.991 | 1.002 |
| rs7775055 | 6 | 32657916 | HGVM8040778 | 3.00E-174 | Genotyped | 0.3718 | 0.6598 |
| rs1270942 | 6 | 31918860 | HGVM280139 | 2.00E-165 | Genotyped | 0.5945 | 0.8606 |
| rs9271573 | 6 | 32590501 | HGVM8421389 | 3.00E-154 | Imputed | 0.1999 | 1.265 |
| rs9273363 | 6 | 32626272 | HGVM8421900 | 5.00E-138 | Genotyped | 0.1152 | 0.7607 |
| rs9272346 | 6 | 32604372 | HGVM11028170 | 2.42E-134 | Imputed | 0.2709 | 1.218 |
| rs2395175 | 6 | 32405026 | HGVM1533373 | 3.87E-122 | Genotyped | 0.4252 | 0.8561 |
| rs11889341 | 2 | 191943742 | HGVM4534129 | 6.00E-122 | Genotyped | 0.1892 | 1.281 |
| rs3021304 | 6 | 32575658 | HGVM1578539 | 1.00E-118 | Imputed | 0.6393 | 1.087 |
| rs1150755 | 6 | 32038550 | HGVM276681 | 6.00E-117 | Genotyped | 0.3202 | 0.7698 |
| rs115575857 | 6 | 32659645 | HGVM35526491 | 8.00E-114 | Imputed | 0.898 | 1.035 |
| rs3135394 | 6 | 32408497 | HGVM1601050 | 5.00E-113 | Genotyped | 0.7184 | 0.9045 |
| rs6457617 | 6 | 32663851 | HGVM6832549 | 5.28E-111 | Genotyped | 0.06649 | 1.379 |
| rs10488631 | 7 | 128594183 | HGVM3212077 | 9.00E-110 | Genotyped | 0.1278 | 0.6706 |
| rs2395163 | 6 | 32387809 | HGVM1533363 | 4.21E-109 | Genotyped | 0.479 | 0.881 |
| rs9268853 | 6 | 32429643 | HGVM8420027 | 5.00E-109 | Genotyped | 0.09684 | 0.7442 |
| rs1150757 | 6 | 32029205 | HGVM276683 | 6.00E-107 | Imputed | 0.6988 | 0.8939 |
| rs17831251 | 2 | 160914156 | HGVM14973625 | 5.00E-103 | Imputed | 0.5348 | 0.8991 |
| rs3763309 | 6 | 32375973 | HGVM6260891 | 1.17E-102 | Genotyped | 0.5259 | 0.8911 |
| rs35000415 | 7 | 128585616 | HGVM20268081 | 1.00E-99 | Imputed | 0.1181 | 0.6625 |
| rs116232857 | 6 | 32597064 | HGVM35576275 | 1.00E-96 | Imputed | 0.2686 | 1.227 |
| rs114092478 | 6 | 32682135 | HGVM35414630 | 3.00E-93 | Imputed | 0.8892 | 1.037 |
| rs2187668 | 6 | 32605884 | HGVM1121679 | 8.00E-93 | Genotyped | 0.7337 | 0.9153 |
| rs3130544 | 6 | 31058340 | HGVM1599167 | 2.00E-90 | Genotyped | 0.1747 | 0.7154 |
| rs17849501 | 1 | 183542323 | HGVM11221850 | 3.00E-88 | Imputed | 0.5435 | 1.254 |
| rs34572943 | 16 | 31272353 | HGVM13528667 | 9.00E-85 | Imputed | 0.6665 | 1.121 |
| rs2395185 | 6 | 32433167 | HGVM1126755 | 1.15E-83 | Genotyped | 0.09684 | 0.7442 |
| rs9275572 | 6 | 32678999 | HGVM8422649 | 2.61E-82 | Genotyped | 0.2732 | 1.223 |
| rs3135388 | 6 | 32413051 | HGVM1601045 | 8.94E-81 | Genotyped | 0.537 | 1.185 |
| rs2516049 | 6 | 32570400 | HGVM1131094 | 1.43E-79 | Genotyped | 0.06688 | 0.7279 |
| rs7937915 | 11 | 2214388 | HGVM8199396 | 4.00E-79 | Imputed | 0.6065 | 1.111 |
| rs9273368 | 6 | 32626475 | HGVM8421904 | 3.00E-78 | Genotyped | 0.1152 | 0.7607 |
| rs7568275 | 2 | 191966452 | HGVM7835229 | 1.00E-77 | Imputed | 0.1877 | 1.283 |
| rs7310615 | 12 | 111865049 | HGVM7599125 | 2.00E-76 | Imputed | 0.02455 | 0.6798 |
| rs2647012 | 6 | 32664458 | HGVM1134490 | 2.07E-76 | Genotyped | 0.1202 | 1.343 |
| rs7192 | 6 | 32411646 | HGVM14178 | 1.85E-75 | Genotyped | 0.9414 | 0.986 |
| rs9268832 | 6 | 32427789 | HGVM8420011 | 4.56E-75 | Genotyped | 0.866 | 1.033 |
| rs3117242 | 6 | 33069893 | HGVM6245462 | 1.00E-71 | Genotyped | 0.3975 | 0.8462 |
| rs12706861 | 7 | 128616582 | HGVM5351826 | 4.00E-71 | Imputed | 0.1687 | 0.697 |
| rs9277341 | 6 | 33039625 | HGVM8423326 | 6.00E-71 | Genotyped | 0.5907 | 0.9095 |
| rs2395157 | 6 | 32348145 | HGVM1533358 | 1.76E-70 | Genotyped | 0.5889 | 0.9074 |
| rs10801908 | 1 | 117090493 | HGVM3507877 | 5.00E-70 | Imputed | 0.6099 | 0.8821 |
| rs3817973 | 6 | 32361111 | HGVM6262650 | 7.41E-70 | Genotyped | 0.2211 | 1.233 |
| rs9275390 | 6 | 32669156 | HGVM8422521 | 9.20E-70 | Genotyped | 0.03665 | 0.6852 |
| rs6932542 | 6 | 32380262 | HGVM7252374 | 9.25E-70 | Genotyped | 0.3039 | 1.195 |
| rs4424066 | 6 | 32354428 | HGVM2543996 | 1.23E-69 | Genotyped | 0.2211 | 1.233 |
| rs9275383 | 6 | 32668846 | HGVM8422514 | 3.28E-69 | Imputed | 0.114 | 0.7204 |
| rs7582694 | 2 | 191970120 | HGVM7849699 | 4.00E-69 | Genotyped | 0.2015 | 1.275 |
| rs5000634 | 6 | 32663564 | HGVM6400525 | 2.16E-68 | Genotyped | 0.1482 | 1.279 |
| rs9268542 | 6 | 32384721 | HGVM8419933 | 4.33E-67 | Genotyped | 0.8026 | 1.044 |
| rs3817963 | 6 | 32368087 | HGVM2310349 | 4.43E-67 | Genotyped | 0.6102 | 0.9135 |
| rs9275313 | 6 | 32665759 | HGVM8422457 | 7.06E-67 | Genotyped | 0.1009 | 0.7335 |
| rs3957148 | 6 | 32682137 | HGVM2365774 | 1.20E-66 | Genotyped | 0.08137 | 0.6938 |
| rs2281388 | 6 | 33060118 | HGVM1124369 | 2.00E-65 | Genotyped | 0.8564 | 0.8585 |
| rs11256593 | 10 | 6117322 | HGVM3965039 | 3.00E-65 | Imputed | 0.3349 | 1.181 |
| rs992969 | 9 | 6209697 | HGVM735360 | 3.00E-64 | Genotyped | 8.21E-03 | 1.793 |
| rs1008723 | 17 | 38066267 | HGVM958777 | 2.00E-62 | Imputed | 0.2701 | 1.205 |
| rs230540 | 4 | 103496667 | HGVM614768 | 2.00E-61 | Imputed | 0.1883 | 1.277 |
| rs547077 | 6 | 32289318 | HGVM260056 | 8.09E-61 | Genotyped | 0.3257 | 0.8448 |
| rs72823641 | 2 | 102936159 | HGVM22243472 | 2.00E-60 | Imputed | 0.6576 | 1.119 |
| rs9268384 | 6 | 32336586 | HGVM8419825 | 5.22E-60 | Genotyped | 0.4547 | 0.8796 |
| rs9275312 | 6 | 32665728 | HGVM8422456 | 9.90E-60 | Genotyped | 0.06163 | 0.6922 |
| rs9275328 | 6 | 32666822 | HGVM8422469 | 1.02E-59 | Genotyped | 0.06163 | 0.6922 |
| rs9405090 | 6 | 32298372 | HGVM8535628 | 1.31E-59 | Genotyped | 0.4547 | 0.8796 |
| rs9268132 | 6 | 32254654 | HGVM8419689 | 1.51E-59 | Genotyped | 0.3405 | 0.8484 |
| rs1033500 | 6 | 32307382 | HGVM275090 | 1.80E-59 | Genotyped | 0.4547 | 0.8796 |
| rs547261 | 6 | 32282033 | HGVM260066 | 2.20E-59 | Genotyped | 0.4547 | 0.8796 |
| rs9268368 | 6 | 32333955 | HGVM8419817 | 3.26E-59 | Genotyped | 0.4547 | 0.8796 |
| rs13192471 | 6 | 32671103 | HGVM5839446 | 2.00E-58 | Genotyped | 0.3853 | 0.832 |
| rs601945 | 6 | 32573415 | HGVM262648 | 1.53E-57 | Imputed | 0.1038 | 0.7397 |
| rs3129871 | 6 | 32406342 | HGVM1598782 | 1.87E-57 | Genotyped | 0.906 | 0.9781 |
| rs2647050 | 6 | 32669767 | HGVM1134518 | 1.02E-55 | Genotyped | 0.4792 | 1.141 |
| rs2856718 | 6 | 32670255 | HGVM1142097 | 1.59E-55 | Genotyped | 0.4792 | 1.141 |
| rs16898264 | 6 | 32677152 | HGVM11050006 | 3.06E-55 | Genotyped | 0.4937 | 1.137 |
| rs6679677 | 1 | 114303808 | HGVM7011783 | 1.00E-53 | Imputed | 0.04668 | 0.6068 |
| rs35472514 | 16 | 31283323 | HGVM13551554 | 4.00E-53 | Imputed | 0.4867 | 1.19 |
| rs3131379 | 6 | 31721033 | HGVM1599538 | 1.71E-52 | Genotyped | 0.7794 | 0.9248 |
| rs2395173 | 6 | 32404859 | HGVM1533371 | 2.35E-52 | Genotyped | 0.86 | 0.9672 |
| rs1837253 | 5 | 110401872 | HGVM659305 | 1.00E-51 | Genotyped | 0.6903 | 0.9261 |
| rs4947296 | 6 | 31058178 | HGVM2874567 | 4.00E-51 | Genotyped | 0.3978 | 1.331 |
| rs9277554 | 6 | 33055538 | HGVM8423530 | 2.00E-50 | Genotyped | 0.3461 | 0.8309 |
| rs438613 | 3 | 28072086 | HGVM149540 | 2.00E-49 | Imputed | 0.9913 | 1.002 |
| rs3087243 | 2 | 204738919 | HGVM1690397 | 7.00E-49 | Genotyped | 0.5859 | 1.097 |
| rs1444782 | 10 | 9058671 | HGVM829215 | 1.00E-48 | Imputed | 0.1887 | 1.264 |
| rs7936312 | 11 | 76293726 | HGVM8197812 | 1.00E-48 | Imputed | 0.7186 | 1.064 |
| rs3757387 | 7 | 128576086 | HGVM9697538 | 1.00E-48 | Imputed | 0.7981 | 0.956 |
| rs7111341 | 11 | 2213166 | HGVM7418467 | 4.43E-48 | Genotyped | 0.9085 | 1.023 |
| rs1800693 | 12 | 6440009 | HGVM5196 | 2.00E-47 | Genotyped | 0.5148 | 0.8929 |
| rs2104286 | 10 | 6099045 | HGVM1277163 | 2.00E-47 | Genotyped | 0.8442 | 0.9614 |
| rs3135338 | 6 | 32401217 | HGVM1601012 | 5.55E-47 | Genotyped | 0.86 | 0.9672 |
| rs7775397 | 6 | 32261252 | HGVM8041113 | 8.00E-47 | Genotyped | 0.8212 | 0.9361 |
| rs1794282 | 6 | 32666526 | HGVM9834780 | 2.60E-46 | Genotyped | 0.9974 | 0.999 |
| rs12927355 | 16 | 11194771 | HGVM5572356 | 6.00E-46 | Imputed | 0.5202 | 1.12 |
| rs10036748 | 5 | 150458146 | HGVM2932717 | 1.00E-45 | Genotyped | 0.2404 | 1.241 |
| rs9272219 | 6 | 32602269 | HGVM10528836 | 1.00E-45 | Genotyped | 0.6576 | 1.093 |
| rs72743461 | 15 | 67441750 | HGVM21660443 | 4.00E-45 | Imputed | 0.02227 | 1.578 |
| rs2858331 | 6 | 32681277 | HGVM1142241 | 5.61E-45 | Genotyped | 0.7573 | 1.056 |
| rs2239804 | 6 | 32411523 | HGVM1123066 | 1.40E-44 | Genotyped | 0.4241 | 0.8672 |
| rs653178 | 12 | 112007756 | HGVM867561 | 2.00E-44 | Imputed | 7.98E-03 | 0.6191 |
| rs7745656 | 6 | 32680970 | HGVM8012349 | 3.64E-44 | Genotyped | 0.3879 | 1.212 |
| rs3129890 | 6 | 32414273 | HGVM1598143 | 4.36E-43 | Genotyped | 0.7657 | 0.9356 |
| rs10174238 | 2 | 191973034 | HGVM3047136 | 3.00E-42 | Imputed | 0.1426 | 1.317 |
| rs2261033 | 6 | 31603591 | HGVM1123843 | 3.09E-42 | Genotyped | 0.3533 | 0.8553 |
| rs34290285 | 2 | 242698640 | HGVM15014773 | 4.00E-42 | Imputed | 0.3249 | 0.8345 |
| rs6903608 | 6 | 32428285 | HGVM7225388 | 4.55E-42 | Genotyped | 5.94E-03 | 1.805 |
| rs7574865 | 2 | 191964633 | HGVM7841836 | 5.00E-42 | Genotyped | 0.1866 | 1.285 |
| rs6677309 | 1 | 117080166 | HGVM7009520 | 5.00E-42 | Genotyped | 0.4543 | 0.8345 |
| rs9275595 | 6 | 32681355 | HGVM8422671 | 1.72E-41 | Genotyped | 0.04849 | 0.6965 |
| rs2072633 | 6 | 31919578 | HGVM573275 | 3.44E-41 | Genotyped | 0.5426 | 1.107 |
| rs9275592 | 6 | 32680620 | HGVM8422669 | 1.00E-40 | Genotyped | 0.07749 | 0.6896 |
| rs2061831 | 8 | 11339882 | HGVM1176499 | 1.00E-40 | Imputed | 0.5922 | 0.8985 |
| rs3129934 | 6 | 32336187 | HGVM1598805 | 2.60E-40 | Genotyped | 0.7776 | 1.071 |
| rs477515 | 6 | 32569691 | HGVM256482 | 4.00E-40 | Genotyped | 0.06688 | 0.7279 |
| rs3806156 | 6 | 32373698 | HGVM2301806 | 7.00E-40 | Genotyped | 0.425 | 0.8705 |
| rs1443438 | 9 | 100550028 | HGVM1204936 | 1.00E-39 | Genotyped | 0.4949 | 1.128 |
| rs9272729 | 6 | 32609594 | HGVM9990068 | 2.00E-39 | Imputed | 0.6136 | 1.154 |
| rs9275141 | 6 | 32651117 | HGVM8422348 | 2.17E-39 | Genotyped | 0.2879 | 1.2 |
| rs9270986 | 6 | 32574060 | HGVM11147441 | 2.38E-39 | Genotyped | 0.1413 | 1.458 |
| rs61839660 | 10 | 6094697 | HGVM11776317 | 3.00E-39 | Imputed | 0.127 | 1.638 |
| rs9275371 | 6 | 32668296 | HGVM8422502 | 3.07E-39 | Genotyped | 0.03665 | 0.6852 |
| rs9275388 | 6 | 32669084 | HGVM8422519 | 6.04E-39 | Genotyped | 0.03665 | 0.6852 |
| rs3135363 | 6 | 32389648 | HGVM1601026 | 8.24E-39 | Genotyped | 0.5792 | 1.123 |
| rs13238352 | 7 | 128647942 | HGVM5885665 | 1.00E-38 | Imputed | 0.1687 | 0.697 |
| rs12722495 | 10 | 6097283 | HGVM5367076 | 1.00E-38 | Imputed | 0.2554 | 1.395 |
| rs3129882 | 6 | 32409530 | HGVM1598793 | 1.54E-38 | Genotyped | 0.3713 | 1.182 |
| rs3184504 | 12 | 111884608 | HGVM1711117 | 2.00E-38 | Genotyped | 0.01724 | 0.661 |
| rs7763262 | 6 | 32424882 | HGVM8029394 | 2.00E-38 | Genotyped | 0.3843 | 1.196 |
| rs9296015 | 6 | 32218989 | HGVM8439581 | 2.00E-38 | Genotyped | 0.4692 | 0.849 |
| rs17484292 | 1 | 183300050 | HGVM19104960 | 1.00E-37 | Imputed | 0.191 | 1.766 |
| rs3129900 | 6 | 32305979 | HGVM2241132 | 2.63E-37 | Genotyped | 0.7776 | 1.071 |
| rs2071295 | 6 | 32038700 | HGVM571942 | 2.88E-37 | Genotyped | 0.527 | 1.115 |
| rs2239689 | 6 | 32030284 | HGVM1120803 | 7.21E-37 | Genotyped | 0.4913 | 1.125 |
| rs9271588 | 6 | 32590953 | HGVM8421402 | 9.00E-37 | Genotyped | 0.06047 | 1.399 |
| rs6670198 | 1 | 2520527 | HGVM7002832 | 2.00E-36 | Imputed | 0.03021 | 1.488 |
| rs10975416 | 9 | 6051924 | HGVM3682308 | 5.00E-36 | Imputed | 0.1819 | 0.7418 |
| rs62420820 | 6 | 137438057 | HGVM18027700 | 9.00E-36 | Imputed | 0.7594 | 0.9433 |
| rs1738074 | 6 | 159465977 | HGVM683195 | 3.00E-35 | Genotyped | 8.33E-03 | 0.6196 |
| rs28483633 | 6 | 32624874 | HGVM17498833 | 3.00E-35 | Genotyped | 0.4141 | 2.002 |
| rs3129768 | 6 | 32595083 | HGVM1598722 | 3.37E-35 | Genotyped | 0.5835 | 1.139 |
| rs1571878 | 6 | 167540842 | HGVM287002 | 5.00E-35 | Imputed | 0.9291 | 0.9852 |
| rs3129963 | 6 | 32380208 | HGVM1598826 | 1.30E-33 | Genotyped | 0.8665 | 0.9576 |
| rs3916765 | 6 | 32685550 | HGVM2349620 | 1.72E-33 | Genotyped | 0.9451 | 0.9846 |
| rs5912838 | X | 78497118 | HGVM6590831 | 2.00E-33 | Genotyped | 0.1732 | 1.279 |
| rs3129941 | 6 | 32337686 | HGVM1598811 | 2.11E-33 | Genotyped | 0.5673 | 1.131 |
| rs35540610 | 2 | 231121829 | HGVM15102521 | 3.00E-33 | Imputed | 0.01137 | 1.752 |
| rs1323292 | 1 | 192541021 | HGVM1227384 | 4.00E-33 | Imputed | 0.359 | 1.219 |
| rs1077667 | 19 | 6668972 | HGVM9609493 | 8.00E-33 | Genotyped | 4.15E-03 | 1.909 |
| rs615672 | 6 | 32574171 | HGVM263272 | 1.92E-32 | Imputed | 0.5251 | 1.131 |
| rs1813375 | 3 | 28078571 | HGVM1513328 | 2.00E-32 | Imputed | 0.9374 | 0.987 |
| rs2205960 | 1 | 173191475 | HGVM2041997 | 3.00E-32 | Imputed | 0.4313 | 0.8477 |
| rs705705 | 12 | 56435504 | HGVM868233 | 4.00E-32 | Imputed | 0.6405 | 0.9094 |
| rs3131294 | 6 | 32180146 | HGVM1599518 | 4.04E-32 | Genotyped | 0.4102 | 1.261 |
| rs115008099 | 5 | 131991881 | HGVM35065093 | 6.00E-32 | Imputed | 0.3715 | 0.8161 |
| rs2736336 | 8 | 11341870 | HGVM1199545 | 6.00E-32 | Imputed | 0.5628 | 0.89 |
| rs6932056 | 6 | 138242437 | HGVM7251933 | 2.00E-31 | Genotyped | 9.51E-03 | 0.2697 |
| rs35486093 | 1 | 85729820 | HGVM19233598 | 2.00E-31 | Imputed | 0.1747 | 0.6992 |
| rs9275596 | 6 | 32681631 | HGVM8422672 | 3.00E-31 | Genotyped | 0.1458 | 1.333 |
| rs2150879 | 17 | 57859210 | HGVM1412654 | 4.00E-31 | Imputed | 0.5163 | 0.891 |
| rs28688825 | 6 | 32587157 | HGVM11202207 | 5.00E-31 | Imputed | 0.2248 | 1.52 |
| rs12539741 | 7 | 128596805 | HGVM5184354 | 6.00E-31 | Imputed | 0.1278 | 0.6706 |
| rs35441874 | 16 | 11213021 | HGVM13550795 | 1.00E-30 | Imputed | 0.0199 | 1.618 |
| rs701006 | 12 | 58106836 | HGVM411239 | 1.00E-30 | Genotyped | 0.9961 | 0.9991 |
| rs3130315 | 6 | 32220685 | HGVM1599016 | 1.91E-30 | Genotyped | 0.2442 | 0.8159 |
| rs2030519 | 3 | 188119901 | HGVM1033807 | 2.00E-30 | Imputed | 0.1672 | 0.7886 |
| rs3115573 | 6 | 32218843 | HGVM1595868 | 2.19E-30 | Genotyped | 0.2022 | 0.7989 |
| rs9843355 | 3 | 119228508 | HGVM8831607 | 4.00E-30 | Imputed | 0.0574 | 1.521 |
| rs9275224 | 6 | 32659878 | HGVM8422427 | 6.00E-30 | Genotyped | 0.08802 | 1.349 |
| rs707939 | 6 | 31726688 | HGVM570181 | 7.33E-30 | Genotyped | 0.6609 | 1.078 |
| rs502055 | 6 | 32579003 | HGVM257738 | 1.00E-29 | Imputed | 0.5133 | 1.142 |
| rs13239597 | 7 | 128695983 | HGVM5886918 | 1.00E-29 | Imputed | 0.1278 | 0.6706 |
| rs9267873 | 6 | 32199352 | HGVM8419591 | 1.04E-29 | Genotyped | 0.438 | 1.148 |
| rs2050190 | 6 | 32339076 | HGVM570867 | 1.14E-29 | Genotyped | 0.4837 | 1.148 |
| rs2070600 | 6 | 32151443 | HGVM571318 | 1.20E-29 | Genotyped | 0.6604 | 0.8769 |
| rs2075800 | 6 | 31777946 | HGVM570178 | 1.98E-29 | Imputed | 0.528 | 1.115 |
| rs7752903 | 6 | 138227364 | HGVM8019356 | 2.00E-29 | Imputed | 4.23E-03 | 0.2408 |
| rs77000060 | 6 | 138237989 | HGVM28744652 | 2.00E-29 | Imputed | 6.56E-03 | 0.2558 |
| rs4939490 | 11 | 60793651 | HGVM2868868 | 2.00E-29 | Imputed | 0.2215 | 1.242 |
| rs17753641 | 3 | 159647674 | HGVM15700076 | 2.00E-29 | Imputed | 0.4667 | 1.231 |
| rs204999 | 6 | 32109979 | HGVM247181 | 3.53E-29 | Genotyped | 0.2699 | 0.8007 |
| rs5029939 | 6 | 138195723 | HGVM6426387 | 5.00E-29 | Genotyped | 0.01976 | 0.3267 |
| rs1150754 | 6 | 32050758 | HGVM276680 | 6.00E-29 | Genotyped | 0.3202 | 0.7698 |
| rs2227139 | 6 | 32413459 | HGVM1527334 | 6.73E-29 | Genotyped | 0.9414 | 0.986 |
| rs4151657 | 6 | 31917540 | HGVM2449993 | 7.42E-29 | Genotyped | 0.4278 | 1.141 |
| rs312729 | 17 | 68306837 | HGVM481336 | 8.00E-29 | Genotyped | 0.5384 | 0.8983 |
| rs55684690 | 11 | 76057946 | HGVM11924422 | 8.00E-29 | Imputed | 0.5549 | 0.6924 |
| rs72928038 | 6 | 90976768 | HGVM23322558 | 8.00E-29 | Imputed | 0.6739 | 1.097 |
| rs4664308 | 2 | 160917497 | HGVM2667735 | 9.00E-29 | Imputed | 0.5895 | 0.9109 |
| rs1980493 | 6 | 32363215 | HGVM689592 | 1.01E-28 | Genotyped | 0.9173 | 1.029 |
| rs4530903 | 6 | 32581889 | HGVM2596685 | 1.31E-28 | Genotyped | 0.8445 | 0.9536 |
| rs1026916 | 17 | 40529835 | HGVM1711606 | 2.00E-28 | Genotyped | 0.5198 | 0.8976 |
| rs10063294 | 5 | 35877505 | HGVM2954201 | 2.00E-28 | Genotyped | 0.7672 | 1.055 |
| rs3130287 | 6 | 32050544 | HGVM1599006 | 2.26E-28 | Genotyped | 0.7843 | 1.074 |
| rs1014486 | 3 | 159691112 | HGVM1495193 | 3.00E-28 | Genotyped | 0.9672 | 1.007 |
| rs34536443 | 19 | 10463118 | HGVM14309152 | 4.00E-28 | Imputed | 0.3305 | 0.4842 |
| rs3129943 | 6 | 32338695 | HGVM1598812 | 4.96E-28 | Genotyped | 0.5142 | 0.8713 |
| rs2431697 | 5 | 159879978 | HGVM1103569 | 8.00E-28 | Genotyped | 0.5135 | 0.8919 |
| rs2395182 | 6 | 32413317 | HGVM1533377 | 8.86E-28 | Genotyped | 0.891 | 0.9696 |
| rs3122929 | 12 | 57509102 | HGVM1710924 | 1.00E-27 | Imputed | 0.7606 | 0.9513 |
| rs2738058 | 8 | 6821617 | HGVM1200081 | 2.00E-27 | Imputed | 0.1805 | 1.268 |
| rs11079784 | 17 | 45702280 | HGVM3787303 | 2.00E-27 | Imputed | 0.2469 | 1.22 |
| rs2596560 | 6 | 31355318 | HGVM1133495 | 2.83E-27 | Genotyped | 0.9615 | 1.009 |
| rs2292239 | 12 | 56482180 | HGVM1339937 | 3.00E-27 | Genotyped | 0.5619 | 0.8928 |
| rs17264332 | 6 | 138005515 | HGVM17487187 | 3.00E-27 | Imputed | 0.6082 | 1.106 |
| rs4244808 | 11 | 2163110 | HGVM2458055 | 4.00E-27 | Imputed | 0.3019 | 0.8468 |
| rs61816761 | 1 | 152285861 | HGVM19777687 | 5.00E-27 | Imputed | 0.8677 | 0.9013 |
| rs910049 | 6 | 32315727 | HGVM272447 | 5.42E-27 | Genotyped | 0.4611 | 1.171 |
| rs1003878 | 6 | 32299822 | HGVM274497 | 7.31E-27 | Genotyped | 0.4946 | 0.8599 |
| rs926070 | 6 | 32257566 | HGVM272830 | 7.95E-27 | Genotyped | 0.7063 | 0.9324 |
| rs3748817 | 1 | 2525665 | HGVM2263808 | 1.00E-26 | Imputed | 0.02311 | 1.508 |
| rs41286801 | 1 | 92975464 | HGVM19281406 | 1.00E-26 | Imputed | 0.759 | 0.9327 |
| rs2736428 | 6 | 31843924 | HGVM1135744 | 1.67E-26 | Genotyped | 0.5311 | 1.113 |
| rs3130340 | 6 | 32244627 | HGVM1599025 | 1.75E-26 | Genotyped | 0.6062 | 0.8872 |
| rs3115553 | 6 | 32245827 | HGVM1595858 | 1.93E-26 | Genotyped | 0.6062 | 0.8872 |
| rs9888739 | 16 | 31313253 | HGVM8869745 | 2.02E-26 | Genotyped | 0.7735 | 1.073 |
| rs6935269 | 6 | 32260350 | HGVM7254924 | 2.25E-26 | Genotyped | 0.6062 | 0.8872 |
| rs8111 | 6 | 32083175 | HGVM10573 | 2.32E-26 | Genotyped | 0.8324 | 1.037 |
| rs10484565 | 6 | 32795032 | HGVM3209080 | 3.00E-26 | Genotyped | 0.3774 | 1.31 |
| rs2734335 | 6 | 31893944 | HGVM1135524 | 3.65E-26 | Genotyped | 0.8556 | 0.9688 |
| rs6589706 | 11 | 118747813 | HGVM6947734 | 5.00E-26 | Genotyped | 0.5804 | 1.1 |
| rs6941112 | 6 | 31946614 | HGVM7260323 | 7.10E-26 | Genotyped | 0.8933 | 1.022 |
| rs9268005 | 6 | 32224388 | HGVM8419648 | 8.73E-26 | Genotyped | 0.7063 | 0.9324 |
| rs12123821 | 1 | 152179152 | HGVM4768715 | 1.00E-25 | Imputed | NA | NA |
| rs377763 | 6 | 32199144 | HGVM253472 | 1.36E-25 | Genotyped | 0.5503 | 0.8846 |
| rs3135353 | 6 | 32392877 | HGVM1601024 | 1.78E-25 | Genotyped | 0.7205 | 0.9097 |
| rs6638512 | X | 5184702 | HGVM6978698 | 2.00E-25 | Imputed | 0.06216 | 1.396 |
| rs6590330 | 11 | 128311059 | HGVM6948303 | 2.00E-25 | Imputed | 0.1426 | 0.6402 |
| rs2849015 | 6 | 32198936 | HGVM1141621 | 3.71E-25 | Genotyped | 0.6532 | 0.9197 |
| rs3129860 | 6 | 32401079 | HGVM1598772 | 3.84E-25 | Genotyped | 0.5244 | 1.186 |
| rs631204 | 6 | 137959455 | HGVM263870 | 5.00E-25 | Genotyped | 0.05028 | 0.7128 |
| rs7454108 | 6 | 32681483 | HGVM7726294 | 5.00E-25 | Genotyped | 0.06338 | 0.6761 |
| rs11749040 | 5 | 40396425 | HGVM4393571 | 5.00E-25 | Imputed | 0.1057 | 0.6488 |
| rs3115663 | 6 | 31601843 | HGVM1595926 | 5.56E-25 | Genotyped | 0.9083 | 1.026 |
| rs4808760 | 19 | 18301979 | HGVM2776035 | 6.00E-25 | Imputed | 0.9272 | 1.018 |
| rs805294 | 6 | 31688217 | HGVM269800 | 7.28E-25 | Genotyped | 0.8302 | 0.9635 |
| rs4282438 | 6 | 33072172 | HGVM2475800 | 9.00E-25 | Genotyped | 0.5464 | 0.624 |
| rs36014129 | 6 | 25884519 | HGVM17618664 | 1.00E-24 | Imputed | 0.1504 | 0.6239 |
| rs1972809 | X | 119867475 | HGVM1965187 | 1.00E-24 | Imputed | 0.3146 | 1.228 |
| rs2255214 | 3 | 121770539 | HGVM1037165 | 1.00E-24 | Imputed | 0.6632 | 1.074 |
| rs3117583 | 6 | 31619576 | HGVM1596519 | 1.20E-24 | Genotyped | 0.9083 | 1.026 |
| rs3130618 | 6 | 31632134 | HGVM1599211 | 1.56E-24 | Genotyped | 0.9083 | 1.026 |
| rs404890 | 6 | 32198867 | HGVM1765801 | 1.66E-24 | Genotyped | 0.6532 | 0.9197 |
| rs9275428 | 6 | 32670978 | HGVM8422551 | 2.00E-24 | Genotyped | 0.03665 | 0.6852 |
| rs13263709 | 8 | 81287175 | HGVM5911254 | 2.00E-24 | Imputed | 0.3138 | 0.8341 |
| rs11554159 | 19 | 18285944 | HGVM4207651 | 2.00E-24 | Imputed | 0.6214 | 1.099 |
| rs7812879 | 8 | 11340181 | HGVM8078315 | 2.00E-24 | Imputed | 0.91 | 1.027 |
| rs12478539 | 2 | 43355324 | HGVM5123070 | 2.00E-24 | Imputed | 0.9112 | 0.9794 |
| rs58166386 | 19 | 16559421 | HGVM14391429 | 4.00E-24 | Imputed | 0.04385 | 0.6874 |
| rs6889239 | 5 | 150457771 | HGVM7211829 | 4.00E-24 | Imputed | 0.1874 | 1.274 |
| rs71508903 | 10 | 63779871 | HGVM20979891 | 5.00E-24 | Imputed | 0.3312 | 0.8241 |
| rs7977720 | 12 | 9866349 | HGVM8239031 | 5.00E-24 | Imputed | 0.6627 | 1.078 |
| rs2070197 | 7 | 128589000 | HGVM1150025 | 6.00E-24 | Imputed | 0.1307 | 0.6712 |
| rs312691 | 17 | 68326338 | HGVM1409296 | 6.00E-24 | Genotyped | 0.6721 | 0.9232 |
| rs3117098 | 6 | 32358513 | HGVM1596356 | 6.22E-24 | Genotyped | 0.2158 | 1.294 |
| rs12101261 | 14 | 81451229 | HGVM4746391 | 7.00E-24 | Imputed | 0.6248 | 1.091 |
| rs6899623 | 6 | 90986559 | HGVM7221730 | 9.00E-24 | Imputed | 0.3275 | 1.189 |
| rs2732549 | 11 | 35088399 | HGVM1327787 | 1.00E-23 | Genotyped | 0.03948 | 1.449 |
| rs1131265 | 3 | 119222456 | HGVM4016708 | 1.00E-23 | Imputed | 0.0574 | 1.521 |
| rs34947566 | 16 | 11412926 | HGVM13538306 | 1.00E-23 | Imputed | 0.6811 | 1.095 |
| rs4728142 | 7 | 128573967 | HGVM2714829 | 1.00E-23 | Genotyped | 0.9484 | 0.9887 |
| rs210120 | 6 | 33574413 | HGVM247709 | 1.13E-23 | Genotyped | 0.8962 | 1.022 |
| rs9356551 | 6 | 167400345 | HGVM8493258 | 2.00E-23 | Imputed | 0.08311 | 1.414 |
| rs1250551 | 10 | 81059335 | HGVM825560 | 2.00E-23 | Genotyped | 0.3177 | 1.192 |
| rs72784452 | 10 | 9111826 | HGVM21026583 | 2.00E-23 | Imputed | 0.4063 | 1.315 |
| rs11571297 | 2 | 204745003 | HGVM4216121 | 2.00E-23 | Imputed | 0.6502 | 1.081 |
| rs17066096 | 6 | 137452908 | HGVM17480781 | 2.00E-23 | Imputed | 0.8268 | 0.9596 |
| rs1004446 | 11 | 2170143 | HGVM849153 | 3.00E-23 | Genotyped | 0.4729 | 0.8837 |
| rs4917014 | 7 | 50305863 | HGVM2853251 | 3.00E-23 | Genotyped | 0.5253 | 1.123 |
| rs2736177 | 6 | 31586094 | HGVM1135738 | 3.15E-23 | Genotyped | 0.8964 | 1.036 |
| rs6457536 | 6 | 32273765 | HGVM6832503 | 3.65E-23 | Genotyped | 0.7731 | 0.9348 |
| rs9989735 | 2 | 231115454 | HGVM8958191 | 4.00E-23 | Imputed | 0.01027 | 1.846 |
| rs532098 | 6 | 32578052 | HGVM259243 | 4.00E-23 | Genotyped | 0.1637 | 1.263 |
| rs34383631 | 11 | 60793330 | HGVM11847379 | 4.00E-23 | Imputed | 0.2214 | 1.238 |
| rs58721818 | 6 | 138243739 | HGVM17840364 | 5.00E-23 | Imputed | 9.51E-03 | 0.2697 |
| rs34725611 | 19 | 10477067 | HGVM14312997 | 5.00E-23 | Imputed | 0.1281 | 1.361 |
| rs7731626 | 5 | 55444683 | HGVM7998590 | 8.00E-23 | Imputed | 0.2821 | 1.221 |
| rs2763979 | 6 | 31794592 | HGVM1137650 | 8.74E-23 | Genotyped | 0.8917 | 0.9768 |
| rs7775228 | 6 | 32658079 | HGVM8040950 | 8.83E-23 | Genotyped | 0.9038 | 0.9658 |
| rs623011 | 17 | 68259446 | HGVM485073 | 1.00E-22 | Imputed | 0.3524 | 0.8372 |
| rs2241116 | 2 | 103003265 | HGVM1660239 | 1.00E-22 | Genotyped | 0.5761 | 0.883 |
| rs707928 | 6 | 31742590 | HGVM669482 | 1.00E-22 | Genotyped | 0.9553 | 0.99 |
| rs494620 | 6 | 31838713 | HGVM6384451 | 1.10E-22 | Genotyped | 0.6907 | 0.935 |
| rs2071554 | 6 | 32784676 | HGVM572183 | 1.37E-22 | Imputed | 0.7952 | 1.084 |
| rs2736332 | 8 | 11339965 | HGVM1199541 | 2.00E-22 | Imputed | 0.2977 | 0.8206 |
| rs7444 | 22 | 21976934 | HGVM14381 | 2.00E-22 | Genotyped | 0.5412 | 1.118 |
| rs2273017 | 6 | 32337630 | HGVM1528014 | 2.00E-22 | Genotyped | 0.7252 | 0.9403 |
| rs6936204 | 6 | 32217092 | HGVM7255805 | 2.98E-22 | Genotyped | 0.6834 | 0.9211 |
| rs2051549 | 6 | 32730086 | HGVM1521830 | 3.00E-22 | Genotyped | 0.3822 | 1.154 |
| rs9314614 | 8 | 6697731 | HGVM8457869 | 5.00E-22 | Imputed | 0.198 | 0.8004 |
| rs28703878 | 8 | 79417222 | HGVM18097193 | 5.00E-22 | Imputed | 0.4089 | 0.8335 |
| rs2736337 | 8 | 11341880 | HGVM1199546 | 5.00E-22 | Imputed | 0.5487 | 0.8863 |
| rs12716641 | 8 | 6898998 | HGVM5361639 | 5.00E-22 | Imputed | 0.705 | 1.068 |
| rs2074488 | 6 | 31240431 | HGVM568938 | 6.65E-22 | Genotyped | 0.7988 | 1.061 |
| rs12928725 | 16 | 31283996 | HGVM5573730 | 7.00E-22 | Imputed | 0.5385 | 1.167 |
| rs1920296 | 3 | 121543577 | HGVM1517718 | 7.00E-22 | Imputed | 0.7616 | 1.057 |
| rs9469220 | 6 | 32658310 | HGVM8584534 | 7.00E-22 | Genotyped | 0.9046 | 1.02 |
| rs9275772 | 6 | 32689503 | HGVM11028201 | 7.53E-22 | Genotyped | 0.5222 | 1.125 |
| rs9268615 | 6 | 32402889 | HGVM8419963 | 9.07E-22 | Genotyped | 0.8758 | 1.027 |
| rs3809627 | 16 | 30103160 | HGVM2304348 | 1.00E-21 | Imputed | 0.1034 | 0.7564 |
| rs117710327 | 19 | 33726578 | HGVM33029259 | 1.00E-21 | Imputed | 0.662 | 1.229 |
| rs140522 | 22 | 50971266 | HGVM1645988 | 1.00E-21 | Genotyped | 0.8613 | 0.9697 |
| rs2395150 | 6 | 32326045 | HGVM1533352 | 1.65E-21 | Genotyped | 0.7515 | 0.9462 |
| rs12617656 | 2 | 162851147 | HGVM5262436 | 2.00E-21 | Imputed | 0.3926 | 0.8556 |
| rs2431098 | 5 | 159887336 | HGVM1103242 | 3.00E-21 | Imputed | 0.1294 | 1.302 |
| rs67111717 | 5 | 176790162 | HGVM22986347 | 3.00E-21 | Imputed | 0.4613 | 1.146 |
| rs117633859 | 1 | 67627828 | HGVM30740005 | 3.00E-21 | Imputed | 0.7142 | 1.207 |
| rs9275793 | 6 | 32690027 | HGVM11028205 | 3.13E-21 | Genotyped | 0.5222 | 1.125 |
| rs4409785 | 11 | 95311422 | HGVM2536975 | 4.00E-21 | Genotyped | 0.3124 | 1.262 |
| rs389883 | 6 | 31947460 | HGVM666106 | 4.74E-21 | Genotyped | 0.6485 | 1.093 |
| rs2301888 | 1 | 17672730 | HGVM1242341 | 5.00E-21 | Genotyped | 0.3435 | 1.19 |
| rs9271850 | 6 | 32595060 | HGVM17463922 | 5.37E-21 | Genotyped | 0.09394 | 1.394 |
| rs59655222 | 1 | 200875897 | HGVM19594218 | 6.00E-21 | Imputed | 0.05154 | 0.673 |
| rs9267649 | 6 | 31824828 | HGVM8419523 | 6.41E-21 | Genotyped | 0.3313 | 0.7856 |
| rs212405 | 6 | 159470559 | HGVM247873 | 8.00E-21 | Imputed | 0.03885 | 0.6579 |
| rs558702 | 6 | 31870326 | HGVM260641 | 8.00E-21 | Genotyped | 0.5074 | 0.8285 |
| rs1131665 | 11 | 613208 | HGVM10000 | 9.00E-21 | Imputed | 0.8382 | 0.961 |
| rs12087340 | 1 | 85746993 | HGVM4732429 | 1.00E-20 | Imputed | 0.2213 | 0.7166 |
| rs3794060 | 11 | 71187679 | HGVM6261882 | 1.00E-20 | Genotyped | 0.2472 | 1.242 |
| rs2071550 | 6 | 32730940 | HGVM572178 | 1.05E-20 | Genotyped | 0.3895 | 0.8577 |
| rs3134945 | 6 | 32146492 | HGVM1600843 | 1.10E-20 | Genotyped | 0.1545 | 0.7261 |
| rs7755852 | 6 | 31277988 | HGVM8022218 | 1.98E-20 | Genotyped | 0.1954 | 1.232 |
| rs3893464 | 6 | 29935250 | HGVM272203 | 2.00E-20 | Genotyped | 0.06709 | 1.371 |
| rs74796499 | 14 | 88432328 | HGVM25696422 | 2.00E-20 | Imputed | 0.2643 | 0.5578 |
| rs7202844 | 16 | 75247391 | HGVM7506600 | 2.00E-20 | Imputed | 0.3267 | 1.328 |
| rs11171739 | 12 | 56470625 | HGVM3879801 | 2.00E-20 | Genotyped | 0.5834 | 0.9042 |
| rs9271640 | 6 | 32592200 | HGVM11146781 | 2.00E-20 | Genotyped | 0.5835 | 1.139 |
| rs11860650 | 16 | 31326706 | HGVM4505381 | 2.00E-20 | Imputed | 0.6262 | 1.129 |
| rs11154801 | 6 | 135739355 | HGVM3862813 | 2.00E-20 | Genotyped | 0.8092 | 1.045 |
| rs2894249 | 6 | 32325835 | HGVM1545590 | 3.49E-20 | Genotyped | 0.5673 | 1.131 |
| rs4796791 | 17 | 40530763 | HGVM2767202 | 4.00E-20 | Genotyped | 0.5881 | 0.9127 |
| rs6594500 | 5 | 110470994 | HGVM6951906 | 4.00E-20 | Imputed | 0.8356 | 0.9644 |
| rs204990 | 6 | 32161430 | HGVM664028 | 4.32E-20 | Genotyped | 0.243 | 0.7639 |
| rs2856705 | 6 | 32670956 | HGVM1142088 | 4.34E-20 | Genotyped | 0.5972 | 1.205 |
| rs1359062 | 1 | 192541472 | HGVM1227893 | 5.00E-20 | Imputed | 0.4414 | 1.176 |
| rs2331964 | 3 | 121542898 | HGVM1530298 | 5.00E-20 | Imputed | 0.6883 | 1.076 |
| rs229527 | 22 | 37581485 | HGVM551789 | 5.00E-20 | Genotyped | 0.7118 | 1.065 |
| rs7090512 | 10 | 6110829 | HGVM7398582 | 5.00E-20 | Genotyped | 0.7585 | 0.9453 |
| rs2857161 | 6 | 32759297 | HGVM1142195 | 5.42E-20 | Genotyped | 0.3989 | 1.154 |
| rs2621383 | 6 | 32759335 | HGVM1134055 | 5.42E-20 | Genotyped | 0.3989 | 1.154 |
| rs71624119 | 5 | 55440730 | HGVM23031634 | 6.00E-20 | Imputed | 0.2497 | 1.289 |
| rs6990534 | 8 | 128814091 | HGVM7307742 | 6.00E-20 | Genotyped | 0.3351 | 1.194 |
| rs2736340 | 8 | 11343973 | HGVM1199547 | 6.00E-20 | Genotyped | 0.5274 | 0.8814 |
| rs17406680 | 10 | 9208204 | HGVM11403435 | 6.00E-20 | Imputed | 0.9053 | 1.054 |
| rs437179 | 6 | 31929014 | HGVM573272 | 6.15E-20 | Genotyped | 0.9421 | 1.014 |
| rs17208888 | 6 | 32379506 | HGVM10699357 | 6.34E-20 | Imputed | 0.02054 | 2.587 |
| rs3129939 | 6 | 32336766 | HGVM1598810 | 6.97E-20 | Genotyped | 0.6922 | 0.9054 |
| rs705699 | 12 | 56384804 | HGVM868227 | 7.00E-20 | Genotyped | 0.5206 | 0.89 |
| rs6880778 | 5 | 40399096 | HGVM7203768 | 8.00E-20 | Imputed | 0.6644 | 1.081 |
| rs2327586 | 6 | 135495226 | HGVM2078233 | 9.00E-20 | Imputed | 0.07959 | 0.7244 |
| rs760293 | 6 | 31611777 | HGVM1604290 | 9.72E-20 | Genotyped | 0.7417 | 0.9242 |
| rs10152595 | 15 | 67475488 | HGVM3029188 | 1.00E-19 | Imputed | 0.05633 | 1.508 |
| rs9591325 | 13 | 50811220 | HGVM8688614 | 1.00E-19 | Genotyped | 0.1129 | 1.757 |
| rs12925972 | 16 | 79111297 | HGVM5570972 | 1.00E-19 | Imputed | 0.2594 | 1.217 |
| rs2233424 | 6 | 44233921 | HGVM1122503 | 1.00E-19 | Imputed | 0.2929 | 0.659 |
| rs9610458 | 22 | 22205353 | HGVM8705232 | 1.00E-19 | Imputed | 0.7874 | 1.045 |
| rs1042663 | 6 | 31905130 | HGVM1115587 | 1.12E-19 | Genotyped | 0.2137 | 1.558 |
| rs241425 | 6 | 32804909 | HGVM250176 | 1.31E-19 | Genotyped | 0.6197 | 0.9153 |
| rs3134954 | 6 | 32071893 | HGVM1600852 | 1.39E-19 | Genotyped | 0.7843 | 1.074 |
| rs9501626 | 6 | 32400344 | HGVM8610149 | 1.42E-19 | Genotyped | 0.7513 | 1.099 |
| rs9296021 | 6 | 32297690 | HGVM8439587 | 1.90E-19 | Genotyped | 0.4547 | 0.8796 |
| rs12612769 | 2 | 191953998 | HGVM5257544 | 2.00E-19 | Imputed | 0.0601 | 1.474 |
| rs12365699 | 11 | 118743286 | HGVM5010344 | 2.00E-19 | Genotyped | 0.2345 | 1.379 |
| rs7190997 | 16 | 31368178 | HGVM7495047 | 2.00E-19 | Genotyped | 0.395 | 0.8615 |
| rs1177228 | 2 | 61242410 | HGVM998580 | 2.00E-19 | Imputed | 0.4191 | 1.173 |
| rs34986765 | 15 | 61069201 | HGVM13263553 | 2.00E-19 | Imputed | 0.5627 | 1.147 |
| rs2248137 | 20 | 52789743 | HGVM1449438 | 2.00E-19 | Imputed | 0.5669 | 1.108 |
| rs2524279 | 6 | 31392906 | HGVM6169076 | 2.99E-19 | Genotyped | 0.5335 | 1.216 |
| rs8056814 | 16 | 75252327 | HGVM8317749 | 3.00E-19 | Imputed | 0.1076 | 1.704 |
| rs1087056 | 10 | 31395761 | HGVM826820 | 3.00E-19 | Imputed | 0.2827 | 1.217 |
| rs1076928 | 6 | 36348689 | HGVM9620269 | 3.00E-19 | Genotyped | 0.5545 | 1.105 |
| rs704840 | 1 | 173226195 | HGVM84363 | 3.00E-19 | Genotyped | 0.7031 | 0.9338 |
| rs12537284 | 7 | 128717906 | HGVM5181898 | 3.61E-19 | Imputed | 0.01134 | 0.5272 |
| rs204991 | 6 | 32161366 | HGVM664029 | 3.74E-19 | Genotyped | 0.243 | 0.7639 |
| rs55838263 | 1 | 200874728 | HGVM19306510 | 4.00E-19 | Imputed | 0.04923 | 0.6691 |
| rs8026898 | 15 | 69991417 | HGVM8288055 | 4.00E-19 | Imputed | 0.2933 | 0.8155 |
| rs28834106 | 19 | 10592144 | HGVM14296852 | 4.00E-19 | Imputed | 0.4432 | 1.162 |
| rs10912578 | 1 | 173251856 | HGVM3619113 | 4.00E-19 | Imputed | 0.6657 | 1.081 |
| rs241427 | 6 | 32804414 | HGVM250178 | 4.34E-19 | Genotyped | 0.5179 | 0.8885 |
| rs722086 | 6 | 25428954 | HGVM1782094 | 5.00E-19 | Genotyped | 0.9496 | 0.9863 |
| rs2546890 | 5 | 158759900 | HGVM1106900 | 5.00E-19 | Genotyped | 0.1206 | 0.7643 |
| rs35789010 | 6 | 25514179 | HGVM17606993 | 5.00E-19 | Imputed | 0.1927 | 0.6546 |
| rs60600003 | 7 | 37382465 | HGVM20562699 | 5.00E-19 | Imputed | 0.2173 | 0.687 |
| rs6032662 | 20 | 44734310 | HGVM6684809 | 5.00E-19 | Imputed | 0.4013 | 1.203 |
| rs9271366 | 6 | 32586854 | HGVM8421336 | 5.57E-19 | Genotyped | 0.3674 | 1.275 |
| rs8062322 | 16 | 11092319 | HGVM8323225 | 5.61E-19 | Genotyped | 0.9055 | 1.022 |
| rs2233434 | 6 | 44232920 | HGVM1122707 | 6.00E-19 | Genotyped | 0.2929 | 0.659 |
| rs3093024 | 6 | 167532793 | HGVM1589373 | 8.00E-19 | Genotyped | 0.9513 | 1.01 |
| rs9268528 | 6 | 32383108 | HGVM8419927 | 8.18E-19 | Genotyped | 0.8026 | 1.044 |
| rs7382297 | 6 | 31247067 | HGVM7661771 | 9.76E-19 | Genotyped | 0.7516 | 1.081 |
| rs12619531 | 2 | 182057640 | HGVM5264315 | 1.00E-18 | Imputed | 0.2812 | 0.8187 |
| rs10951042 | 7 | 3139417 | HGVM3657803 | 1.00E-18 | Imputed | 0.3836 | 1.153 |
| rs3873444 | 6 | 32682724 | HGVM272195 | 1.06E-18 | Genotyped | 0.8648 | 1.06 |
| rs2596437 | 6 | 31339086 | HGVM2150708 | 1.19E-18 | Genotyped | 0.7274 | 0.9203 |
| rs3130952 | 6 | 31177915 | HGVM9963435 | 1.19E-18 | Genotyped | 0.7516 | 1.081 |
| rs6920220 | 6 | 138006504 | HGVM7240882 | 2.00E-18 | Genotyped | 0.6082 | 1.106 |
| rs9653442 | 2 | 100825367 | HGVM8734227 | 2.00E-18 | Imputed | 0.717 | 1.062 |
| rs13332649 | 16 | 85966683 | HGVM5980592 | 2.00E-18 | Imputed | 0.7544 | 0.9373 |
| rs9878602 | 3 | 71535338 | HGVM8861550 | 2.00E-18 | Imputed | 0.7948 | 0.9571 |
| rs1265883 | 1 | 160464911 | HGVM785672 | 2.00E-18 | Imputed | 0.9319 | 1.027 |
| rs8032939 | 15 | 38834033 | HGVM8294051 | 2.00E-18 | Imputed | 0.9943 | 1.001 |
| rs3132946 | 6 | 32190028 | HGVM1599976 | 2.31E-18 | Genotyped | 0.3613 | 1.291 |
| rs11066188 | 12 | 112610714 | HGVM3773606 | 2.67E-18 | Imputed | 0.09231 | 0.7358 |
| rs3130532 | 6 | 31208453 | HGVM1599159 | 2.82E-18 | Genotyped | 0.7516 | 1.081 |
| rs10494079 | 1 | 108374875 | HGVM3217485 | 3.00E-18 | Imputed | 0.496 | 0.8137 |
| rs12708716 | 16 | 11179873 | HGVM5353691 | 3.00E-18 | Imputed | 0.6152 | 1.091 |
| rs1465697 | 19 | 49837246 | HGVM522903 | 3.00E-18 | Imputed | 0.7673 | 1.063 |
| rs7864027 | 9 | 6504189 | HGVM8129085 | 4.00E-18 | Imputed | 0.4045 | 1.225 |
| rs2111485 | 2 | 163110536 | HGVM6138632 | 4.00E-18 | Genotyped | 0.4143 | 0.8685 |
| rs4976646 | 5 | 176788570 | HGVM2894483 | 4.00E-18 | Genotyped | 0.4471 | 1.15 |
| rs4239702 | 20 | 44749251 | HGVM2454615 | 4.00E-18 | Imputed | 0.5664 | 1.134 |
| rs706778 | 10 | 6098949 | HGVM817038 | 4.00E-18 | Imputed | 0.9548 | 1.01 |
| rs1701704 | 12 | 56412487 | HGVM880359 | 5.00E-18 | Genotyped | 0.5873 | 0.8985 |
| rs2858870 | 6 | 32572251 | HGVM1141168 | 5.54E-18 | Genotyped | 0.6232 | 0.8683 |
| rs2523485 | 6 | 31351035 | HGVM1130558 | 5.80E-18 | Genotyped | 0.8921 | 0.965 |
| rs17696736 | 12 | 112486818 | HGVM12232301 | 6.00E-18 | Genotyped | 0.0492 | 0.7003 |
| rs10821944 | 10 | 63785089 | HGVM3528025 | 6.00E-18 | Imputed | 0.1559 | 0.7692 |
| rs8083786 | 18 | 12881361 | HGVM8344588 | 6.00E-18 | Imputed | 0.3325 | 0.7799 |
| rs6498184 | 16 | 11435990 | HGVM6870135 | 7.00E-18 | Genotyped | 0.8324 | 1.047 |
| rs11172086 | 12 | 57449206 | HGVM3880148 | 8.00E-18 | Imputed | 0.284 | 0.7483 |
| rs2227956 | 6 | 31778272 | HGVM1122411 | 8.19E-18 | Genotyped | 0.47 | 0.8359 |
| rs2230926 | 6 | 138196066 | HGVM6149168 | 1.00E-17 | Genotyped | 8.87E-03 | 0.2905 |
| rs2523393 | 6 | 29705659 | HGVM1131917 | 1.00E-17 | Genotyped | 0.1787 | 1.275 |
| rs4637409 | 4 | 102753408 | HGVM2649937 | 1.00E-17 | Genotyped | 0.3201 | 0.8316 |
| rs483180 | 1 | 120267505 | HGVM755596 | 1.00E-17 | Imputed | 0.9583 | 0.9908 |
| rs11644034 | 16 | 85972612 | HGVM4288374 | 1.00E-17 | Genotyped | 0.9897 | 0.9974 |
| rs3134603 | 6 | 32126002 | HGVM1600746 | 1.82E-17 | Genotyped | 0.13 | 1.511 |
| rs1980422 | 2 | 204610396 | HGVM6129285 | 2.00E-17 | Imputed | 0.05175 | 0.6698 |
| rs10168266 | 2 | 191935804 | HGVM3041934 | 2.00E-17 | Genotyped | 0.05804 | 1.506 |
| rs74956615 | 19 | 10427721 | HGVM26532837 | 2.00E-17 | Imputed | 0.4933 | 0.6244 |
| rs1887428 | 9 | 4984530 | HGVM1937181 | 2.00E-17 | Imputed | 0.8254 | 1.04 |
| rs3024622 | 16 | 27365453 | HGVM1729856 | 2.00E-17 | Imputed | 0.9589 | 1.009 |
| rs1024161 | 2 | 204721752 | HGVM996846 | 2.00E-17 | Imputed | 0.9611 | 1.008 |
| rs1112718 | 10 | 94479107 | HGVM822750 | 2.00E-17 | Imputed | 0.9885 | 1.002 |
| rs35703946 | 16 | 86021505 | HGVM13557305 | 3.00E-17 | Imputed | 0.1163 | 0.6534 |
| rs10178845 | 2 | 8443803 | HGVM3051078 | 3.00E-17 | Imputed | 0.2265 | 1.245 |
| rs13389408 | 2 | 191933283 | HGVM6037660 | 3.00E-17 | Imputed | 0.2459 | 1.378 |
| rs111945767 | 6 | 32517045 | HGVM35288049 | 3.00E-17 | Imputed | 0.3026 | 1.228 |
| rs28383364 | 6 | 32606912 | HGVM17495870 | 3.00E-17 | Imputed | 0.743 | 1.098 |
| rs1015166 | 6 | 32798731 | HGVM274749 | 3.94E-17 | Genotyped | 0.6436 | 1.09 |
| rs13426947 | 2 | 191933254 | HGVM6075400 | 4.00E-17 | Imputed | 0.09622 | 1.422 |
| rs12434551 | 14 | 69253364 | HGVM5079015 | 4.00E-17 | Imputed | 0.2432 | 0.8216 |
| rs13099273 | 3 | 188133518 | HGVM5745298 | 4.00E-17 | Imputed | 0.3393 | 0.8432 |
| rs10845606 | 12 | 12834894 | HGVM3551800 | 4.00E-17 | Imputed | 0.4503 | 0.8612 |
| rs6881706 | 5 | 35879156 | HGVM7204660 | 4.00E-17 | Imputed | 0.568 | 1.116 |
| rs17513503 | 5 | 110146446 | HGVM16949322 | 4.00E-17 | Imputed | 0.6718 | 0.8926 |
| rs7923837 | 10 | 94481917 | HGVM8185381 | 4.00E-17 | Genotyped | 0.8806 | 0.9746 |
| rs10028805 | 4 | 102737250 | HGVM2926003 | 4.00E-17 | Genotyped | 0.884 | 1.026 |
| rs9272723 | 6 | 32609427 | HGVM10846018 | 4.76E-17 | Imputed | 0.1926 | 1.264 |
| rs3101942 | 6 | 32870057 | HGVM1591719 | 4.82E-17 | Genotyped | 0.5723 | 0.9043 |
| rs12722502 | 10 | 6093139 | HGVM5367083 | 6.00E-17 | Imputed | 0.1008 | 0.3153 |
| rs10954214 | 7 | 128589633 | HGVM3660984 | 7.00E-17 | Imputed | 0.2751 | 0.8207 |
| rs9652601 | 16 | 11174365 | HGVM8733510 | 7.00E-17 | Imputed | 0.6499 | 1.083 |
| rs3807307 | 7 | 128579202 | HGVM6262248 | 7.00E-17 | Imputed | 0.8276 | 1.039 |
| rs6705628 | 2 | 74208362 | HGVM7036337 | 7.00E-17 | Imputed | 0.8909 | 0.892 |
| rs2905747 | 6 | 31451476 | HGVM2207845 | 7.07E-17 | Genotyped | 0.3962 | 1.19 |
| rs9268557 | 6 | 32389305 | HGVM8419938 | 7.37E-17 | Genotyped | 0.9568 | 1.01 |
| rs9268877 | 6 | 32431147 | HGVM8420039 | 7.71E-17 | Genotyped | 0.02755 | 1.491 |
| rs389884 | 6 | 31940897 | HGVM666107 | 7.86E-17 | Genotyped | 0.6988 | 0.8939 |
| rs486416 | 6 | 31856070 | HGVM256930 | 7.87E-17 | Genotyped | 0.4462 | 0.8581 |
| rs1893217 | 18 | 12809340 | HGVM976103 | 8.00E-17 | Genotyped | 0.1244 | 0.6724 |
| rs34026809 | 11 | 118480695 | HGVM11832758 | 8.00E-17 | Imputed | 0.6588 | 0.8046 |
| rs62013236 | 15 | 79247482 | HGVM13466310 | 9.00E-17 | Imputed | 0.06141 | 0.6173 |
| rs1021156 | 8 | 79575804 | HGVM1495433 | 9.00E-17 | Genotyped | 0.09557 | 0.7184 |
| rs9909593 | 17 | 37970149 | HGVM8888930 | 9.00E-17 | Imputed | 0.541 | 1.113 |
| rs6736175 | 2 | 191946322 | HGVM7065662 | 9.00E-17 | Imputed | 0.6008 | 1.096 |
| rs9268861 | 6 | 32429894 | HGVM8420035 | 9.32E-17 | Genotyped | 0.2094 | 0.7415 |
| rs7766843 | 6 | 32430729 | HGVM8032858 | 9.44E-17 | Genotyped | 0.4493 | 0.8394 |
| rs12379034 | 9 | 123162666 | HGVM5023390 | 1.00E-16 | Genotyped | 0.06501 | 1.531 |
| rs6901084 | 6 | 32736936 | HGVM7223077 | 1.00E-16 | Genotyped | 0.13 | 0.7828 |
| rs909685 | 22 | 39747671 | HGVM1800985 | 1.00E-16 | Imputed | 0.1821 | 0.784 |
| rs2284178 | 6 | 31432125 | HGVM1124464 | 1.00E-16 | Genotyped | 0.1942 | 1.258 |
| rs4639966 | 11 | 118573519 | HGVM2651226 | 1.00E-16 | Genotyped | 0.2264 | 0.7922 |
| rs652888 | 6 | 31851234 | HGVM264782 | 1.00E-16 | Imputed | 0.2489 | 0.7632 |
| rs2071469 | 6 | 32784783 | HGVM572104 | 1.00E-16 | Genotyped | 0.2496 | 0.8162 |
| rs2442749 | 6 | 31352040 | HGVM1127702 | 1.00E-16 | Genotyped | 0.2638 | 0.8202 |
| rs131654 | 22 | 21917190 | HGVM548789 | 1.00E-16 | Genotyped | 0.3139 | 1.218 |
| rs10807113 | 6 | 32722186 | HGVM3513102 | 1.00E-16 | Genotyped | 0.3292 | 1.164 |
| rs1480380 | 6 | 32913246 | HGVM285293 | 1.00E-16 | Genotyped | 0.4157 | 0.7789 |
| rs12177980 | 6 | 32686084 | HGVM4822986 | 1.00E-16 | Genotyped | 0.4757 | 0.8948 |
| rs7762279 | 6 | 32755290 | HGVM8028447 | 1.00E-16 | Genotyped | 0.481 | 0.8114 |
| rs1052486 | 6 | 31610686 | HGVM674350 | 1.00E-16 | Genotyped | 0.4921 | 1.121 |
| rs3131296 | 6 | 32172993 | HGVM1599520 | 1.00E-16 | Genotyped | 0.518 | 0.8511 |
| rs3134942 | 6 | 32168771 | HGVM1600840 | 1.00E-16 | Genotyped | 0.518 | 0.8511 |
| rs705700 | 12 | 56389293 | HGVM868228 | 1.00E-16 | Imputed | 0.5226 | 0.888 |
| rs241452 | 6 | 32796346 | HGVM12856 | 1.00E-16 | Genotyped | 0.53 | 0.8825 |
| rs131658 | 22 | 21917626 | HGVM1023390 | 1.00E-16 | Imputed | 0.5984 | 1.106 |
| rs1265759 | 6 | 32322393 | HGVM279864 | 1.00E-16 | Genotyped | 0.6269 | 0.9191 |
| rs3892710 | 6 | 32682862 | HGVM272059 | 1.00E-16 | Genotyped | 0.6598 | 1.104 |
| rs1894406 | 6 | 32787056 | HGVM685951 | 1.00E-16 | Genotyped | 0.695 | 0.9306 |
| rs1150752 | 6 | 32064726 | HGVM276678 | 1.00E-16 | Imputed | 0.6988 | 0.8939 |
| rs3130299 | 6 | 32203537 | HGVM6246553 | 1.00E-16 | Genotyped | 0.7051 | 1.085 |
| rs11933540 | 4 | 26120001 | HGVM4578415 | 1.00E-16 | Imputed | 0.7097 | 0.9329 |
| rs9267658 | 6 | 31845985 | HGVM8419531 | 1.00E-16 | Genotyped | 0.7129 | 1.104 |
| rs874040 | 4 | 26108197 | HGVM179972 | 1.00E-16 | Imputed | 0.7409 | 0.9379 |
| rs492899 | 6 | 31933518 | HGVM667041 | 1.00E-16 | Genotyped | 0.7564 | 0.9146 |
| rs3117582 | 6 | 31620520 | HGVM1596518 | 1.00E-16 | Genotyped | 0.7794 | 0.9248 |
| rs2855812 | 6 | 31472720 | HGVM1142029 | 1.00E-16 | Genotyped | 0.8727 | 1.034 |
| rs9268530 | 6 | 32383223 | HGVM8419928 | 1.00E-16 | Genotyped | 0.8963 | 0.9667 |
| rs9268856 | 6 | 32429719 | HGVM8420030 | 1.09E-16 | Genotyped | 0.4823 | 0.8507 |
| rs9268858 | 6 | 32429758 | HGVM8420032 | 1.10E-16 | Genotyped | 0.08083 | 0.7317 |
| rs9268862 | 6 | 32430167 | HGVM8420036 | 1.17E-16 | Genotyped | 0.4823 | 0.8507 |
| rs151719 | 6 | 32903900 | HGVM244773 | 1.43E-16 | Genotyped | 0.7401 | 0.9386 |
| rs644045 | 6 | 31883957 | HGVM264421 | 1.84E-16 | Genotyped | 0.5382 | 1.122 |
| rs13327021 | 3 | 27783015 | HGVM5974926 | 2.00E-16 | Genotyped | 0.02688 | 0.6786 |
| rs56817615 | 4 | 10718846 | HGVM16573287 | 2.00E-16 | Imputed | 0.07495 | 1.351 |
| rs7552544 | 1 | 101240893 | HGVM7819541 | 2.00E-16 | Imputed | 0.08202 | 0.7351 |
| rs17339836 | 7 | 128681062 | HGVM20185993 | 2.00E-16 | Imputed | 0.1687 | 0.697 |
| rs741172 | 16 | 11200798 | HGVM472811 | 2.00E-16 | Imputed | 0.5022 | 1.127 |
| rs872863 | 9 | 126154354 | HGVM733742 | 2.00E-16 | Imputed | 0.5276 | 0.8316 |
| rs2041670 | 16 | 11174652 | HGVM1982190 | 2.00E-16 | Genotyped | 0.6003 | 1.096 |
| rs2163226 | 2 | 43361256 | HGVM1657790 | 2.00E-16 | Genotyped | 0.6467 | 0.9189 |
| rs12133753 | 1 | 92222089 | HGVM4778670 | 2.00E-16 | Imputed | 0.8909 | 1.034 |
| rs17321999 | 2 | 30479857 | HGVM14964571 | 2.00E-16 | Imputed | 0.9532 | 1.011 |
| rs6804441 | 3 | 119260944 | HGVM7130981 | 3.00E-16 | Genotyped | 0.04827 | 1.576 |
| rs2542148 | 18 | 12777573 | HGVM2142252 | 3.00E-16 | Imputed | 0.3219 | 0.7778 |
| rs2317231 | 1 | 157686337 | HGVM2073624 | 3.00E-16 | Imputed | 0.591 | 0.9165 |
| rs229541 | 22 | 37591318 | HGVM551795 | 3.00E-16 | Imputed | 0.7969 | 1.044 |
| rs1143678 | 16 | 31343005 | HGVM1834869 | 3.70E-16 | Imputed | 0.5999 | 1.129 |
| rs34695944 | 2 | 61124850 | HGVM15043475 | 4.00E-16 | Imputed | 0.1088 | 1.35 |
| rs4410871 | 8 | 128815029 | HGVM2537514 | 4.00E-16 | Imputed | 0.2414 | 1.241 |
| rs7726414 | 5 | 133431834 | HGVM7993405 | 4.00E-16 | Genotyped | 0.9536 | 0.9821 |
| rs5987194 | X | 153301467 | HGVM6643530 | 5.00E-16 | Imputed | 0.4726 | 0.8542 |
| rs12622670 | 2 | 68646536 | HGVM5267463 | 5.00E-16 | Imputed | 0.4949 | 1.134 |
| rs983494 | 1 | 160703965 | HGVM91065 | 6.00E-16 | Genotyped | 0.7616 | 0.9346 |
| rs2283792 | 22 | 22131125 | HGVM1482551 | 6.00E-16 | Imputed | 0.8806 | 1.025 |
| rs72702900 | 1 | 152771963 | HGVM20850075 | 6.00E-16 | Imputed | 0.8884 | 0.9388 |
| rs10239340 | 7 | 128668510 | HGVM3101214 | 6.98E-16 | Genotyped | 0.6787 | 0.9302 |
| rs6533052 | 4 | 103911781 | HGVM6896145 | 7.00E-16 | Imputed | 0.06673 | 0.7308 |
| rs4810485 | 20 | 44747947 | HGVM2777373 | 8.00E-16 | Genotyped | 0.4431 | 1.183 |
| rs11123811 | 2 | 100760172 | HGVM3831679 | 9.00E-16 | Imputed | 0.4034 | 1.151 |
| rs2855772 | 4 | 55548475 | HGVM1085029 | 1.00E-15 | Imputed | 0.07465 | 2.256 |
| rs2856717 | 6 | 32670308 | HGVM1142096 | 1.00E-15 | Genotyped | 0.1376 | 1.328 |
| rs13385731 | 2 | 33701890 | HGVM6033965 | 1.00E-15 | Genotyped | 0.2387 | 0.6554 |
| rs72922276 | 1 | 65429319 | HGVM20873614 | 1.00E-15 | Imputed | 0.2615 | 1.45 |
| rs4796224 | 17 | 34842521 | HGVM2766709 | 1.00E-15 | Imputed | 0.5078 | 1.125 |
| rs58029167 | 9 | 5850375 | HGVM18726833 | 1.00E-15 | Imputed | 0.8213 | 1.043 |
| rs11203202 | 21 | 43825357 | HGVM3911363 | 1.00E-15 | Imputed | 0.9978 | 0.9995 |
| rs7535818 | 1 | 192545099 | HGVM7803011 | 1.51E-15 | Genotyped | 0.5429 | 1.134 |
| rs6498168 | 16 | 11235130 | HGVM6870119 | 1.98E-15 | Imputed | 0.2088 | 0.803 |
| rs1870071 | 19 | 16505106 | HGVM1933281 | 2.00E-15 | Imputed | 0.07986 | 0.7264 |
| rs2371108 | 3 | 27757018 | HGVM1042412 | 2.00E-15 | Imputed | 0.09614 | 1.344 |
| rs12147246 | 14 | 103265844 | HGVM4792188 | 2.00E-15 | Imputed | 0.1187 | 1.296 |
| rs12722559 | 10 | 6070273 | HGVM5367129 | 2.00E-15 | Imputed | 0.3794 | 0.8035 |
| rs1734787 | X | 153325446 | HGVM1486519 | 2.00E-15 | Genotyped | 0.4524 | 0.8464 |
| rs28412876 | 17 | 47454515 | HGVM13788536 | 2.00E-15 | Imputed | 0.6476 | 0.9227 |
| rs7005834 | 8 | 134214204 | HGVM7322509 | 2.00E-15 | Imputed | 0.7436 | 0.9409 |
| rs1801133 | 1 | 11856378 | HGVM2390 | 2.00E-15 | Genotyped | 0.8446 | 1.036 |
| rs669607 | 3 | 28071444 | HGVM145182 | 2.00E-15 | Genotyped | 0.9913 | 1.002 |
| rs2157082 | 6 | 32760714 | HGVM2022480 | 2.07E-15 | Genotyped | 0.4093 | 1.151 |
| rs2857154 | 6 | 32762616 | HGVM1142189 | 2.18E-15 | Genotyped | 0.5705 | 1.108 |
| rs35570272 | 3 | 33047662 | HGVM15815536 | 3.00E-15 | Imputed | 0.07982 | 0.7394 |
| rs1782645 | 10 | 81048611 | HGVM832734 | 3.00E-15 | Genotyped | 0.1013 | 1.332 |
| rs9736016 | 11 | 118724894 | HGVM8771694 | 3.00E-15 | Imputed | 0.6413 | 1.086 |
| rs3825932 | 15 | 79235446 | HGVM2315589 | 3.00E-15 | Imputed | 0.7033 | 1.072 |
| rs1858037 | 2 | 65598300 | HGVM1010471 | 3.00E-15 | Imputed | 0.7847 | 0.9549 |
| rs405343 | 16 | 1067832 | HGVM9353753 | 3.00E-15 | Imputed | 0.8601 | 1.044 |
| rs11574914 | 9 | 34710338 | HGVM4219389 | 3.00E-15 | Genotyped | 0.9961 | 0.9991 |
| rs7453920 | 6 | 32730012 | HGVM7726124 | 3.99E-15 | Genotyped | 0.3822 | 1.154 |
| rs1250568 | 10 | 81045280 | HGVM825575 | 4.00E-15 | Imputed | 0.06484 | 1.396 |
| rs7655915 | 4 | 149633468 | HGVM7923134 | 4.00E-15 | Imputed | 0.1995 | 0.7701 |
| rs12416116 | 10 | 90035654 | HGVM5060538 | 4.00E-15 | Imputed | 0.231 | 0.7899 |
| rs1610677 | 6 | 29789171 | HGVM681100 | 4.00E-15 | Genotyped | 0.3214 | 0.8521 |
| rs9373839 | 6 | 106655617 | HGVM8508386 | 4.00E-15 | Imputed | 0.3738 | 1.228 |
| rs17849502 | 1 | 183532580 | HGVM11189972 | 4.00E-15 | Imputed | 0.4484 | 1.323 |
| rs6762714 | 3 | 188470238 | HGVM7091193 | 4.00E-15 | Imputed | 0.4908 | 0.892 |
| rs10021288 | 4 | 123005534 | HGVM2919672 | 4.00E-15 | Imputed | 0.6053 | 0.9003 |
| rs9276432 | 6 | 32712384 | HGVM8422879 | 4.28E-15 | Genotyped | 0.4746 | 1.125 |
| rs7768538 | 6 | 32729821 | HGVM8034491 | 4.59E-15 | Genotyped | 0.3822 | 1.154 |
| rs3828840 | 6 | 32520907 | HGVM2317511 | 5.00E-15 | Genotyped | 0.644 | 1.084 |
| rs9276429 | 6 | 32712104 | HGVM8422876 | 5.39E-15 | Genotyped | 0.5322 | 1.111 |
| rs3130311 | 6 | 32217367 | HGVM2241190 | 5.76E-15 | Genotyped | 0.2442 | 0.8159 |
| rs2299864 | 6 | 106667994 | HGVM1124887 | 6.00E-15 | Imputed | 0.3947 | 1.213 |
| rs2289583 | 15 | 75311036 | HGVM2066011 | 6.00E-15 | Genotyped | 0.3977 | 0.8453 |
| rs523604 | 11 | 118755738 | HGVM373244 | 6.00E-15 | Genotyped | 0.8126 | 1.04 |
| rs8107548 | 19 | 49870643 | HGVM8368242 | 6.00E-15 | Imputed | 0.9029 | 1.026 |
| rs574710 | 6 | 32288190 | HGVM261477 | 6.03E-15 | Genotyped | 0.3804 | 0.8611 |
| rs1854853 | 6 | 167533062 | HGVM686204 | 7.00E-15 | Imputed | 0.7819 | 0.9551 |
| rs926591 | 6 | 32305690 | HGVM272873 | 7.12E-15 | Genotyped | 0.4547 | 0.8796 |
| rs11073328 | 15 | 38764843 | HGVM3780788 | 1.00E-14 | Genotyped | 0.1583 | 1.402 |
| rs2542151 | 18 | 12779947 | HGVM1433217 | 1.00E-14 | Imputed | 0.3219 | 0.7778 |
| rs10018951 | 4 | 184609373 | HGVM2917672 | 1.00E-14 | Genotyped | 0.4824 | 0.8518 |
| rs9405192 | 6 | 382537 | HGVM8535716 | 1.00E-14 | Genotyped | 0.4954 | 1.129 |
| rs77000868 | 10 | 9070677 | HGVM24769796 | 1.00E-14 | Imputed | 0.5739 | 0.7049 |
| rs34593439 | 15 | 79234957 | HGVM13253515 | 1.00E-14 | Imputed | 0.5756 | 1.175 |
| rs11676922 | 2 | 100806940 | HGVM4321311 | 1.00E-14 | Imputed | 0.6998 | 1.066 |
| rs58394161 | 1 | 92939959 | HGVM19504517 | 1.00E-14 | Imputed | 0.7159 | 0.927 |
| rs3862488 | 19 | 23089316 | HGVM6279258 | 1.00E-14 | Genotyped | 0.9426 | 0.976 |
| rs5753037 | 22 | 30581722 | HGVM6440152 | 1.83E-14 | Imputed | 0.5487 | 0.8967 |
| rs1801274 | 1 | 161479745 | HGVM3442 | 2.00E-14 | Genotyped | 0.167 | 1.259 |
| rs12764378 | 10 | 63800004 | HGVM5409027 | 2.00E-14 | Imputed | 0.4151 | 0.845 |
| rs12655443 | 5 | 141467856 | HGVM5300300 | 2.00E-14 | Genotyped | 0.4444 | 1.134 |
| rs1990760 | 2 | 163124051 | HGVM1970084 | 2.00E-14 | Genotyped | 0.5705 | 0.9066 |
| rs2493291 | 1 | 3329384 | HGVM1250254 | 2.00E-14 | Imputed | 0.6981 | 0.9123 |
| rs2841277 | 14 | 105391005 | HGVM1377055 | 2.00E-14 | Genotyped | 0.7992 | 1.042 |
| rs842639 | 2 | 61095245 | HGVM994145 | 2.00E-14 | Genotyped | 0.9112 | 0.9797 |
| rs146644295 | 17 | 37574592 | HGVM45763329 | 2.00E-14 | Imputed | 0.967 | 1.029 |
| rs539703 | 6 | 32288462 | HGVM259665 | 2.14E-14 | Genotyped | 0.4547 | 0.8796 |
| rs2857136 | 6 | 32775686 | HGVM9880608 | 2.17E-14 | Genotyped | 0.5705 | 1.108 |
| rs6902723 | 6 | 32731960 | HGVM7224573 | 2.40E-14 | Genotyped | 0.1401 | 1.272 |
| rs9311676 | 3 | 58470351 | HGVM8454992 | 3.00E-14 | Genotyped | 0.05795 | 0.7045 |
| rs12574073 | 11 | 128319478 | HGVM5218756 | 3.00E-14 | Imputed | 0.1195 | 0.6243 |
| rs244656 | 5 | 133449827 | HGVM205474 | 3.00E-14 | Imputed | 0.4827 | 0.8469 |
| rs73366469 | 7 | 74033600 | HGVM23546228 | 3.00E-14 | Imputed | 0.5481 | 1.212 |
| rs984971 | 2 | 163224521 | HGVM1813290 | 3.00E-14 | Genotyped | 0.6129 | 0.9134 |
| rs3778753 | 7 | 128580042 | HGVM2283531 | 3.00E-14 | Imputed | 0.7747 | 0.9501 |
| rs953387 | 2 | 136907170 | HGVM8636605 | 3.00E-14 | Genotyped | 0.8203 | 0.9594 |
| rs9863496 | 3 | 18798848 | HGVM8848705 | 3.00E-14 | Imputed | 0.8453 | 0.9631 |
| rs3823536 | 7 | 128579666 | HGVM2313946 | 3.00E-14 | Imputed | 0.8695 | 1.029 |
| rs10425559 | 19 | 4837487 | HGVM3162175 | 3.00E-14 | Imputed | 0.8836 | 1.026 |
| rs2738048 | 8 | 6822785 | HGVM1198430 | 3.00E-14 | Genotyped | 0.9567 | 0.9901 |
| rs3737798 | 1 | 160389984 | HGVM2256383 | 4.00E-14 | Imputed | 0.2473 | 1.216 |
| rs6564681 | 16 | 79652720 | HGVM6925304 | 4.00E-14 | Imputed | 0.717 | 1.068 |
| rs2857210 | 6 | 32741742 | HGVM1142213 | 4.10E-14 | Genotyped | 0.5449 | 1.117 |
| rs2621384 | 6 | 32759273 | HGVM1134056 | 4.64E-14 | Genotyped | 0.4099 | 1.15 |
| rs516246 | 19 | 49206172 | HGVM518748 | 5.00E-14 | Genotyped | 0.3948 | 0.8591 |
| rs6677604 | 1 | 196686918 | HGVM7009814 | 5.00E-14 | Genotyped | 0.641 | 0.9034 |
| rs2075302 | 2 | 163076146 | HGVM569726 | 5.00E-14 | Genotyped | 0.8544 | 1.032 |
| rs6568431 | 6 | 106588806 | HGVM6928492 | 5.00E-14 | Genotyped | 0.8829 | 0.9744 |
| rs4852324 | 2 | 74202578 | HGVM2808034 | 6.00E-14 | Genotyped | 0.06216 | 1.852 |
| rs17724508 | 16 | 79350204 | HGVM13491847 | 6.00E-14 | Imputed | 0.852 | 1.054 |
| rs212407 | 6 | 159470417 | HGVM247875 | 7.00E-14 | Imputed | 0.05288 | 0.6786 |
| rs12525220 | 6 | 32675470 | HGVM5169826 | 7.00E-14 | Genotyped | 0.3718 | 0.6598 |
| rs12296430 | 12 | 6503500 | HGVM4941118 | 7.00E-14 | Imputed | 0.811 | 0.9518 |
| rs1059702 | X | 153284192 | HGVM564915 | 8.00E-14 | Genotyped | 0.347 | 0.8074 |
| rs12506688 | 4 | 26104113 | HGVM5151259 | 8.00E-14 | Imputed | 0.9148 | 1.021 |
| rs2755459 | X | 89804127 | HGVM9758109 | 9.00E-14 | Imputed | 0.2173 | 1.304 |
| rs883871 | 17 | 38252660 | HGVM1797979 | 9.00E-14 | Imputed | 0.3769 | 0.8102 |
| rs9308424 | 1 | 212877776 | HGVM8451795 | 9.00E-14 | Imputed | 0.6108 | 0.9047 |
| rs73135369 | 7 | 73940978 | HGVM23520630 | 9.00E-14 | Imputed | 0.9624 | 1.033 |
| rs11085727 | 19 | 10466123 | HGVM3793315 | 1.00E-13 | Imputed | 0.07707 | 1.431 |
| rs5754467 | 22 | 21985094 | HGVM6441442 | 1.00E-13 | Imputed | 0.1002 | 1.381 |
| rs6832151 | 4 | 40303633 | HGVM7157387 | 1.00E-13 | Genotyped | 0.1182 | 0.7343 |
| rs77315098 | 16 | 27381648 | HGVM26121899 | 1.00E-13 | Imputed | 0.3089 | 1.405 |
| rs4794058 | 17 | 45597098 | HGVM2765053 | 1.00E-13 | Imputed | 0.3176 | 1.19 |
| rs7595717 | 2 | 68587477 | HGVM7862769 | 1.00E-13 | Genotyped | 0.3909 | 0.8493 |
| rs2269434 | 11 | 47360412 | HGVM1314410 | 1.00E-13 | Imputed | 0.5858 | 0.9074 |
| rs1534430 | 2 | 12644736 | HGVM1005354 | 1.00E-13 | Genotyped | 0.6915 | 0.9332 |
| rs4248154 | 6 | 31002616 | HGVM2459924 | 1.00E-13 | Genotyped | 0.881 | 0.9685 |
| rs1059312 | 12 | 129278864 | HGVM1706287 | 1.00E-13 | Genotyped | 0.9143 | 1.02 |
| rs3768792 | 2 | 213871709 | HGVM2276750 | 1.00E-13 | Genotyped | 0.9193 | 1.024 |
| rs12251307 | 10 | 6123495 | HGVM4895900 | 1.33E-13 | Genotyped | 0.7706 | 1.082 |
| rs6903130 | 6 | 32732210 | HGVM7224946 | 1.40E-13 | Genotyped | 0.1401 | 1.272 |
| rs719316 | 6 | 16672760 | HGVM1781540 | 2.00E-13 | Imputed | 3.24E-03 | 0.5916 |
| rs7132277 | 12 | 123593382 | HGVM7438229 | 2.00E-13 | Imputed | 0.03588 | 1.585 |
| rs4073285 | 1 | 2539796 | HGVM2415550 | 2.00E-13 | Imputed | 0.03776 | 1.478 |
| rs10181656 | 2 | 191969879 | HGVM3053499 | 2.00E-13 | Imputed | 0.2015 | 1.275 |
| rs6738544 | 2 | 191989356 | HGVM7067945 | 2.00E-13 | Genotyped | 0.3318 | 0.8436 |
| rs1883832 | 20 | 44746982 | HGVM1447955 | 2.00E-13 | Genotyped | 0.4431 | 1.183 |
| rs10892299 | 11 | 118726843 | HGVM3598726 | 2.00E-13 | Imputed | 0.4546 | 1.2 |
| rs2327832 | 6 | 137973068 | HGVM1125610 | 2.00E-13 | Genotyped | 0.4986 | 1.142 |
| rs2248359 | 20 | 52791518 | HGVM1449442 | 2.00E-13 | Genotyped | 0.6521 | 1.079 |
| rs10911628 | 1 | 184649503 | HGVM3618158 | 2.00E-13 | Imputed | 0.6557 | 1.164 |
| rs3761959 | 1 | 157669278 | HGVM2272412 | 2.00E-13 | Imputed | 0.8518 | 1.03 |
| rs3024493 | 1 | 206943968 | HGVM1632424 | 2.00E-13 | Imputed | 0.8607 | 1.04 |
| rs12451295 | 17 | 68376823 | HGVM5095786 | 2.00E-13 | Imputed | 0.9281 | 1.017 |
| rs2254546 | 8 | 11343680 | HGVM1180357 | 2.00E-13 | Imputed | 0.9309 | 1.021 |
| rs2084007 | 5 | 133891282 | HGVM1095728 | 2.00E-13 | Genotyped | 0.9893 | 1.002 |
| rs7975763 | 12 | 123604053 | HGVM8237074 | 3.00E-13 | Imputed | 0.01052 | 1.669 |
| rs387619 | 11 | 35098193 | HGVM367202 | 3.00E-13 | Imputed | 0.07781 | 1.376 |
| rs3747093 | 22 | 21984379 | HGVM2262643 | 3.00E-13 | Imputed | 0.1345 | 1.35 |
| rs1794275 | 6 | 32671248 | HGVM685244 | 3.00E-13 | Genotyped | 0.4393 | 0.8498 |
| rs2093816 | 13 | 43046036 | HGVM1997221 | 3.00E-13 | Imputed | 0.4709 | 1.137 |
| rs16878091 | 4 | 26088128 | HGVM16280105 | 3.00E-13 | Imputed | 0.8946 | 1.026 |
| rs76413021 | 1 | 173206297 | HGVM24354445 | 3.00E-13 | Imputed | 0.9079 | 1.061 |
| rs9268831 | 6 | 32427748 | HGVM8420010 | 3.03E-13 | Genotyped | 0.7535 | 1.056 |
| rs9461799 | 6 | 32689529 | HGVM8578629 | 3.86E-13 | Genotyped | 0.4757 | 0.8948 |
| rs2304256 | 19 | 10475652 | HGVM1442746 | 4.00E-13 | Genotyped | 0.1281 | 1.361 |
| rs6018432 | 20 | 36051846 | HGVM6671482 | 4.00E-13 | Imputed | 0.136 | 1.541 |
| rs7708392 | 5 | 150457485 | HGVM7975467 | 4.00E-13 | Imputed | 0.265 | 1.228 |
| rs59186511 | 13 | 99986238 | HGVM12801062 | 4.00E-13 | Imputed | 0.8831 | 0.9638 |
| rs2076533 | 6 | 32363527 | HGVM570862 | 4.01E-13 | Genotyped | 0.2211 | 1.233 |
| rs4505848 | 4 | 123132492 | HGVM2584237 | 4.68E-13 | Imputed | 0.2741 | 0.8201 |
| rs5029937 | 6 | 138195151 | HGVM6426385 | 5.00E-13 | Imputed | 7.50E-03 | 0.2801 |
| rs12148050 | 14 | 103263788 | HGVM4792992 | 5.00E-13 | Genotyped | 0.1179 | 1.297 |
| rs58688157 | 11 | 625085 | HGVM12047172 | 5.00E-13 | Imputed | 0.5469 | 0.8888 |
| rs7441808 | 4 | 26090375 | HGVM7715003 | 5.00E-13 | Imputed | 0.8691 | 0.9684 |
| rs28617673 | 15 | 67371244 | HGVM13225770 | 5.00E-13 | Imputed | 0.9277 | 0.9784 |
| rs735542 | 8 | 128175696 | HGVM320394 | 6.00E-13 | Imputed | 0.0624 | 1.402 |
| rs34695601 | 14 | 76014298 | HGVM12957158 | 6.00E-13 | Imputed | 0.1064 | 1.422 |
| rs12531711 | 7 | 128617466 | HGVM5176322 | 6.00E-13 | Imputed | 0.1687 | 0.697 |
| rs249677 | 5 | 141539339 | HGVM206952 | 6.00E-13 | Imputed | 0.5724 | 1.105 |
| rs2477923 | 10 | 8565990 | HGVM2130058 | 6.00E-13 | Imputed | 0.8256 | 0.9615 |
| rs11052877 | 12 | 9905690 | HGVM3760186 | 6.00E-13 | Genotyped | 0.8769 | 0.973 |
| rs17630466 | 4 | 26086569 | HGVM16310162 | 6.00E-13 | Imputed | 0.8946 | 1.026 |
| rs2305482 | 17 | 38140927 | HGVM1415183 | 6.00E-13 | Imputed | 0.9799 | 0.9959 |
| rs4713693 | 6 | 33817929 | HGVM2704019 | 7.00E-13 | Genotyped | 0.5862 | 0.9122 |
| rs2105325 | 1 | 173349725 | HGVM2003108 | 7.00E-13 | Imputed | 0.7913 | 1.055 |
| rs11574637 | 16 | 31368874 | HGVM4219171 | 8.00E-13 | Genotyped | 0.3315 | 1.242 |
| rs9273076 | 6 | 32612301 | HGVM17464449 | 8.00E-13 | Imputed | 0.3607 | 1.288 |
| rs11915402 | 3 | 58982075 | HGVM4560233 | 8.00E-13 | Genotyped | 0.5525 | 0.8458 |
| rs13022699 | 2 | 102287835 | HGVM5667974 | 8.00E-13 | Imputed | 0.6807 | 0.8626 |
| rs6496663 | 15 | 90887584 | HGVM6868673 | 1.00E-12 | Imputed | 0.01682 | 1.574 |
| rs3734266 | 6 | 34823187 | HGVM2253959 | 1.00E-12 | Genotyped | 0.1704 | 0.6905 |
| rs6671847 | 1 | 161478810 | HGVM7004391 | 1.00E-12 | Imputed | 0.1775 | 1.255 |
| rs3828058 | 1 | 151786281 | HGVM2316980 | 1.00E-12 | Imputed | 0.1784 | 1.274 |
| rs2847293 | 18 | 12782448 | HGVM1434972 | 1.00E-12 | Imputed | 0.2371 | 0.7426 |
| rs2867461 | 4 | 79513215 | HGVM1540733 | 1.00E-12 | Genotyped | 0.2678 | 0.8246 |
| rs9271100 | 6 | 32576478 | HGVM17463585 | 1.00E-12 | Genotyped | 0.3305 | 1.205 |
| rs11217037 | 11 | 118677086 | HGVM3925237 | 1.00E-12 | Imputed | 0.5181 | 1.163 |
| rs45613035 | 4 | 123141070 | HGVM16525753 | 1.00E-12 | Imputed | 0.627 | 0.86 |
| rs35776863 | 17 | 7226957 | HGVM13843040 | 1.00E-12 | Genotyped | 0.627 | 1.112 |
| rs4426778 | 4 | 102780724 | HGVM2545338 | 1.00E-12 | Imputed | 0.6338 | 0.9197 |
| rs10892279 | 11 | 118611781 | HGVM3598706 | 1.00E-12 | Imputed | 0.6368 | 1.114 |
| rs4820830 | 22 | 30531091 | HGVM2785177 | 1.00E-12 | Imputed | 0.6524 | 0.9222 |
| rs74290525 | 6 | 31835162 | HGVM24174534 | 1.00E-12 | Imputed | 0.8262 | 1.097 |
| rs13330176 | 16 | 86019087 | HGVM5978105 | 1.00E-12 | Imputed | 0.8331 | 1.043 |
| rs11073337 | 15 | 38847763 | HGVM3780798 | 1.00E-12 | Genotyped | 0.9378 | 0.9863 |
| rs2300747 | 1 | 117104215 | HGVM1242300 | 1.74E-12 | Genotyped | 0.6388 | 0.8914 |
| rs13017599 | 2 | 61164331 | HGVM5662857 | 2.00E-12 | Imputed | 0.1184 | 1.341 |
| rs2301271 | 6 | 32725193 | HGVM1124952 | 2.00E-12 | Genotyped | 0.3822 | 1.154 |
| rs9357155 | 6 | 32809848 | HGVM8493820 | 2.00E-12 | Genotyped | 0.389 | 1.23 |
| rs4252665 | 17 | 37885383 | HGVM2461159 | 2.00E-12 | Genotyped | 0.5757 | 1.264 |
| rs6589939 | 11 | 122518525 | HGVM6947946 | 2.00E-12 | Imputed | 0.6531 | 1.081 |
| rs7626218 | 3 | 176852038 | HGVM7893344 | 2.00E-12 | Imputed | 0.6744 | 0.932 |
| rs150260796 | 17 | 38168828 | HGVM45857159 | 2.00E-12 | Imputed | 0.7978 | 1.177 |
| rs34889541 | 1 | 198594769 | HGVM19193595 | 2.00E-12 | Imputed | 0.845 | 1.07 |
| rs9603616 | 13 | 40368069 | HGVM8699570 | 2.00E-12 | Imputed | 0.8474 | 0.9658 |
| rs9981624 | 21 | 43825722 | HGVM8951867 | 2.00E-12 | Imputed | 0.9978 | 0.9995 |
| rs4548893 | 16 | 31364493 | HGVM2605483 | 2.36E-12 | Genotyped | 0.6589 | 1.101 |
| rs9275406 | 6 | 32669955 | HGVM8422537 | 3.00E-12 | Genotyped | 0.03665 | 0.6852 |
| rs17603856 | 6 | 16630898 | HGVM17489815 | 3.00E-12 | Genotyped | 0.04488 | 1.408 |
| rs4760609 | 12 | 48415757 | HGVM2739145 | 3.00E-12 | Imputed | 0.12 | 1.372 |
| rs28929474 | 14 | 94844947 | HGVM12932591 | 3.00E-12 | Imputed | 0.1246 | 2.495 |
| rs2236262 | 14 | 69261472 | HGVM1369657 | 3.00E-12 | Genotyped | 0.1336 | 0.7763 |
| rs970987 | 9 | 21585265 | HGVM1203881 | 3.00E-12 | Imputed | 0.1447 | 0.7626 |
| rs28411352 | 1 | 38278579 | HGVM19113023 | 3.00E-12 | Imputed | 0.4041 | 0.8464 |
| rs10056243 | 5 | 110259077 | HGVM2948473 | 3.00E-12 | Genotyped | 0.774 | 0.9019 |
| rs10131490 | 14 | 68743307 | HGVM3010973 | 3.00E-12 | Imputed | 0.8322 | 0.9593 |
| rs391851 | 5 | 102677920 | HGVM16920014 | 4.00E-12 | Imputed | 0.2452 | 0.8073 |
| rs9967792 | 2 | 191974435 | HGVM8940680 | 4.00E-12 | Imputed | 0.4292 | 0.8704 |
| rs2071591 | 6 | 31515799 | HGVM572221 | 4.00E-12 | Imputed | 0.5867 | 0.9108 |
| rs13415465 | 2 | 100764004 | HGVM6063856 | 4.00E-12 | Imputed | 0.5928 | 1.1 |
| rs597325 | 6 | 91002494 | HGVM262446 | 4.00E-12 | Genotyped | 0.6649 | 0.9295 |
| rs9394159 | 6 | 33618162 | HGVM8526101 | 4.00E-12 | Imputed | 0.6784 | 1.073 |
| rs10760126 | 9 | 123662618 | HGVM3465747 | 4.00E-12 | Imputed | 0.7222 | 1.064 |
| rs802791 | 6 | 106569270 | HGVM269641 | 4.00E-12 | Imputed | 0.7414 | 0.942 |
| rs4262739 | 11 | 128421175 | HGVM2466291 | 4.00E-12 | Imputed | 0.9513 | 0.99 |
| rs17670280 | 3 | 188400239 | HGVM15699047 | 5.00E-12 | Imputed | 0.1735 | 0.7052 |
| rs2412971 | 22 | 30494371 | HGVM2114786 | 5.00E-12 | Imputed | 0.2524 | 1.206 |
| rs17780048 | 6 | 138179146 | HGVM17492018 | 5.00E-12 | Genotyped | 0.3423 | 1.591 |
| rs11755527 | 6 | 90958231 | HGVM4400060 | 5.00E-12 | Imputed | 0.5982 | 1.093 |
| rs1893592 | 21 | 43855067 | HGVM1455123 | 5.00E-12 | Genotyped | 0.6191 | 1.101 |
| rs34330 | 12 | 12870695 | HGVM864348 | 5.00E-12 | Genotyped | 0.6192 | 1.099 |
| rs2289746 | 3 | 105455955 | HGVM1039857 | 5.00E-12 | Genotyped | 0.7262 | 1.064 |
| rs548234 | 6 | 106568034 | HGVM260104 | 5.00E-12 | Imputed | 0.8008 | 0.9548 |
| rs35929052 | 16 | 85994484 | HGVM13563010 | 6.00E-12 | Imputed | 0.157 | 0.67 |
| rs13054355 | 22 | 21997070 | HGVM5699878 | 6.00E-12 | Genotyped | 0.3469 | 1.258 |
| rs2306848 | 7 | 129962414 | HGVM1155828 | 6.00E-12 | Genotyped | 0.5722 | 1.181 |
| rs10499194 | 6 | 138002637 | HGVM3222613 | 6.00E-12 | Imputed | 0.5767 | 0.9021 |
| rs666930 | 1 | 120258970 | HGVM765793 | 6.00E-12 | Genotyped | 0.7089 | 1.067 |
| rs10753074 | 1 | 173346343 | HGVM3458640 | 6.00E-12 | Imputed | 0.8871 | 0.9753 |
| rs793108 | 10 | 31415106 | HGVM818486 | 6.00E-12 | Imputed | 0.911 | 1.02 |
| rs2843401 | 1 | 2528133 | HGVM1269727 | 7.00E-12 | Genotyped | 0.04317 | 1.455 |
| rs7426056 | 2 | 204612058 | HGVM7700293 | 7.00E-12 | Imputed | 0.05175 | 0.6698 |
| rs1128334 | 11 | 128328959 | HGVM849890 | 7.00E-12 | Genotyped | 0.1477 | 0.6437 |
| rs11123810 | 2 | 100759285 | HGVM3831678 | 7.00E-12 | Imputed | 0.4874 | 1.135 |
| rs1913517 | 10 | 50119054 | HGVM1275783 | 7.00E-12 | Genotyped | 0.8782 | 1.027 |
| rs4247364 | 17 | 43336687 | HGVM2459687 | 8.00E-12 | Imputed | 0.5831 | 0.9014 |
| rs7097397 | 10 | 50025396 | HGVM7405211 | 8.00E-12 | Imputed | 0.8024 | 1.048 |
| rs4902647 | 14 | 69254191 | HGVM2843426 | 9.00E-12 | Genotyped | 0.2844 | 0.8358 |
| rs1921445 | 3 | 105948518 | HGVM611060 | 9.00E-12 | Imputed | 0.4329 | 1.168 |
| rs4622329 | 12 | 102321935 | HGVM2642216 | 9.00E-12 | Imputed | 0.486 | 1.122 |
| rs9784858 | 6 | 32787175 | HGVM8788770 | 9.00E-12 | Imputed | 0.863 | 1.05 |
| rs10797431 | 1 | 2501222 | HGVM3503368 | 1.00E-11 | Imputed | 0.04917 | 1.429 |
| rs13031237 | 2 | 61136129 | HGVM5676550 | 1.00E-11 | Imputed | 0.1135 | 1.35 |
| rs12636784 | 3 | 119174383 | HGVM5281613 | 1.00E-11 | Imputed | 0.1683 | 1.357 |
| rs903361 | 1 | 203091274 | HGVM774966 | 1.00E-11 | Genotyped | 0.2717 | 1.203 |
| rs12537 | 22 | 30423460 | HGVM18837 | 1.00E-11 | Genotyped | 0.2811 | 1.202 |
| rs6859219 | 5 | 55438580 | HGVM7183242 | 1.00E-11 | Imputed | 0.3032 | 1.254 |
| rs2297550 | 1 | 206643772 | HGVM2068042 | 1.00E-11 | Imputed | 0.3087 | 0.7716 |
| rs4958880 | 5 | 150438477 | HGVM2882769 | 1.00E-11 | Imputed | 0.3396 | 1.212 |
| rs11697848 | 20 | 48575315 | HGVM4342312 | 1.00E-11 | Imputed | 0.5282 | 1.305 |
| rs8072449 | 17 | 73312184 | HGVM8333308 | 1.00E-11 | Imputed | 0.6237 | 1.114 |
| rs11757201 | 6 | 138003822 | HGVM4401742 | 1.00E-11 | Imputed | 0.6666 | 1.088 |
| rs9282641 | 3 | 121796768 | HGVM574277 | 1.00E-11 | Genotyped | 0.6981 | 1.134 |
| rs35677470 | 3 | 58183636 | HGVM15822046 | 1.00E-11 | Imputed | 0.7033 | 0.8959 |
| rs11150612 | 16 | 31357760 | HGVM3858606 | 1.00E-11 | Imputed | 0.7038 | 1.071 |
| rs3190930 | 6 | 128291199 | HGVM2245415 | 1.00E-11 | Genotyped | 0.7617 | 0.9403 |
| rs1830035 | 7 | 63726645 | HGVM1513872 | 1.00E-11 | Genotyped | 0.8131 | 0.9078 |
| rs3122605 | 1 | 206955041 | HGVM1639190 | 1.00E-11 | Imputed | 0.8305 | 0.9512 |
| rs56994090 | 14 | 101306447 | HGVM13034826 | 1.00E-11 | Imputed | 0.878 | 0.9729 |
| rs7528684 | 1 | 157670816 | HGVM7795973 | 1.00E-11 | Genotyped | 0.954 | 1.009 |
| rs9378815 | 6 | 426155 | HGVM8512518 | 1.00E-11 | Imputed | NA | NA |
| rs2182410 | 10 | 6122669 | HGVM2031809 | 1.15E-11 | Imputed | 0.764 | 1.053 |
| rs1050391 | 6 | 32917857 | HGVM275536 | 1.46E-11 | Genotyped | 0.4135 | 0.7995 |
| rs2611215 | 4 | 166574267 | HGVM1076647 | 2.00E-11 | Imputed | 0.01754 | 1.803 |
| rs864537 | 1 | 167411384 | HGVM773882 | 2.00E-11 | Genotyped | 0.03209 | 0.6874 |
| rs1385374 | 12 | 129300694 | HGVM1706436 | 2.00E-11 | Imputed | 0.04778 | 1.77 |
| rs2288904 | 19 | 10742170 | HGVM1442279 | 2.00E-11 | Genotyped | 0.07052 | 1.514 |
| rs34840245 | 6 | 34812701 | HGVM17557794 | 2.00E-11 | Imputed | 0.1112 | 0.6385 |
| rs7204270 | 16 | 30156963 | HGVM7507985 | 2.00E-11 | Genotyped | 0.1405 | 0.7732 |
| rs2561477 | 5 | 102608924 | HGVM1108169 | 2.00E-11 | Imputed | 0.1469 | 0.7625 |
| rs76668869 | 1 | 80979421 | HGVM24373268 | 2.00E-11 | Imputed | 0.1707 | 2.123 |
| rs11893432 | 2 | 191921874 | HGVM4538225 | 2.00E-11 | Imputed | 0.2176 | 1.288 |
| rs702814 | 7 | 28172732 | HGVM296994 | 2.00E-11 | Imputed | 0.3456 | 0.8539 |
| rs67676925 | 3 | 46274259 | HGVM22558807 | 2.00E-11 | Imputed | 0.4404 | 1.3 |
| rs11217040 | 11 | 118680648 | HGVM3925240 | 2.00E-11 | Imputed | 0.5385 | 1.156 |
| rs2523946 | 6 | 29941943 | HGVM1132437 | 2.00E-11 | Genotyped | 0.607 | 0.9152 |
| rs4821124 | 22 | 21979289 | HGVM2785410 | 2.00E-11 | Imputed | 0.6071 | 1.104 |
| rs3733345 | 4 | 954247 | HGVM2253364 | 2.00E-11 | Imputed | 0.6158 | 1.089 |
| rs1615504 | 18 | 67526644 | HGVM970682 | 2.00E-11 | Imputed | 0.6346 | 0.9214 |
| rs4336042 | 22 | 30552511 | HGVM2501419 | 2.00E-11 | Imputed | 0.6524 | 0.9222 |
| rs17510339 | 5 | 132029414 | HGVM16949282 | 2.00E-11 | Imputed | 0.6956 | 1.108 |
| rs6712515 | 2 | 100806514 | HGVM7042938 | 2.00E-11 | Imputed | 0.7837 | 1.047 |
| rs2834787 | 21 | 36502558 | HGVM1473955 | 2.00E-11 | Imputed | 0.7994 | 1.062 |
| rs11117433 | 16 | 86019516 | HGVM3825266 | 2.00E-11 | Imputed | 0.8466 | 0.9578 |
| rs3093023 | 6 | 167534290 | HGVM1589372 | 2.00E-11 | Imputed | 0.9153 | 0.9824 |
| rs1883414 | 6 | 33086448 | HGVM1516256 | 2.00E-11 | Genotyped | 0.9221 | 1.019 |
| rs12212193 | 6 | 90996769 | HGVM4857249 | 2.48E-11 | Imputed | 0.7507 | 1.055 |
| rs17421624 | 6 | 32066177 | HGVM17488057 | 2.51E-11 | Genotyped | 0.5496 | 1.108 |
| rs6985109 | 8 | 10761585 | HGVM7302537 | 2.51E-11 | Genotyped | 0.6283 | 1.089 |
| rs12149527 | 16 | 79110596 | HGVM4794473 | 3.00E-11 | Imputed | 0.1664 | 0.7914 |
| rs10213692 | 5 | 55442249 | HGVM3080935 | 3.00E-11 | Imputed | 0.3402 | 1.237 |
| rs7601754 | 2 | 191940451 | HGVM7868811 | 3.00E-11 | Genotyped | 0.3481 | 0.8071 |
| rs2451258 | 6 | 159506600 | HGVM1128110 | 3.00E-11 | Genotyped | 0.5983 | 0.9015 |
| rs442309 | 10 | 64490495 | HGVM350590 | 3.00E-11 | Imputed | 0.7789 | 1.051 |
| rs73013527 | 11 | 128496952 | HGVM21180706 | 3.00E-11 | Imputed | 0.8336 | 0.9648 |
| rs13116227 | 4 | 8558266 | HGVM5762401 | 3.00E-11 | Imputed | 0.8626 | 1.031 |
| rs1516971 | 8 | 129542100 | HGVM1509516 | 3.00E-11 | Imputed | 0.9098 | 1.029 |
| rs2530710 | 6 | 30940387 | HGVM1132683 | 3.01E-11 | Genotyped | 0.534 | 1.158 |
| rs10791824 | 11 | 65559266 | HGVM3497728 | 4.00E-11 | Genotyped | 0.203 | 1.232 |
| rs1953126 | 9 | 123640500 | HGVM1958077 | 4.00E-11 | Genotyped | 0.4313 | 0.8722 |
| rs35967351 | 1 | 160711804 | HGVM19266185 | 4.00E-11 | Imputed | 0.6808 | 0.9219 |
| rs1749792 | 16 | 68569440 | HGVM952049 | 4.00E-11 | Imputed | 0.875 | 0.9681 |
| rs2812378 | 9 | 34710260 | HGVM1222007 | 4.00E-11 | Genotyped | 0.965 | 0.9923 |
| rs2516460 | 6 | 31418700 | HGVM9881123 | 4.61E-11 | Genotyped | 0.9639 | 0.9928 |
| rs17266594 | 4 | 102750922 | HGVM16303260 | 4.74E-11 | Imputed | 0.3201 | 0.8316 |
| rs2176082 | 3 | 58331186 | HGVM1525998 | 5.00E-11 | Genotyped | 0.07807 | 1.373 |
| rs1217403 | 1 | 114388804 | HGVM9306580 | 5.00E-11 | Imputed | 0.3815 | 1.194 |
| rs7595037 | 2 | 68647095 | HGVM7862079 | 5.00E-11 | Genotyped | 0.3824 | 1.177 |
| rs703842 | 12 | 58162739 | HGVM572252 | 5.00E-11 | Genotyped | 0.5975 | 1.101 |
| rs4948496 | 10 | 63805617 | HGVM2875422 | 5.00E-11 | Genotyped | 0.8569 | 1.033 |
| rs3891175 | 6 | 32634467 | HGVM268234 | 5.00E-11 | Imputed | 0.9085 | 1.03 |
| rs8043085 | 15 | 38828140 | HGVM8304144 | 5.00E-11 | Imputed | 0.9319 | 1.016 |
| rs4452313 | 3 | 17047032 | HGVM2557798 | 5.00E-11 | Imputed | 0.9392 | 1.014 |
| rs3130981 | 6 | 31083813 | HGVM1599417 | 5.07E-11 | Genotyped | 0.3507 | 1.202 |
| rs7202877 | 16 | 75247245 | HGVM7506632 | 5.71E-11 | Genotyped | 0.3201 | 1.333 |
| rs2181622 | 10 | 6391061 | HGVM1278968 | 6.00E-11 | Imputed | 0.1323 | 0.7399 |
| rs57791671 | 3 | 121608433 | HGVM15971775 | 6.00E-11 | Imputed | 0.1787 | 0.7842 |
| rs212400 | 6 | 159473574 | HGVM247868 | 6.00E-11 | Imputed | 0.2244 | 0.789 |
| rs6659932 | 1 | 67802371 | HGVM6993176 | 6.00E-11 | Imputed | 0.2422 | 0.7453 |
| rs9275563 | 6 | 32677912 | HGVM9975215 | 6.00E-11 | Genotyped | 0.4097 | 1.153 |
| rs1479924 | 4 | 123387600 | HGVM1506465 | 6.00E-11 | Imputed | 0.439 | 1.169 |
| rs10795791 | 10 | 6108340 | HGVM3501717 | 6.00E-11 | Imputed | 0.6886 | 1.073 |
| rs4918037 | 10 | 105413040 | HGVM2854057 | 6.00E-11 | Imputed | 0.767 | 0.936 |
| rs11117431 | 16 | 86015316 | HGVM3825264 | 6.00E-11 | Imputed | 0.8047 | 1.059 |
| rs539514 | 13 | 76326282 | HGVM891878 | 6.00E-11 | Imputed | 0.8823 | 0.9759 |
| rs1320344 | 12 | 103887477 | HGVM877138 | 6.00E-11 | Imputed | 0.974 | 0.9941 |
| rs744600 | 2 | 191564757 | HGVM1693486 | 7.00E-11 | Imputed | 0.08761 | 1.355 |
| rs11868709 | 17 | 76228571 | HGVM4513448 | 7.00E-11 | Imputed | 0.3863 | 0.8468 |
| rs6749371 | 2 | 191902184 | HGVM7078361 | 7.00E-11 | Imputed | 0.5747 | 0.8264 |
| rs12736195 | 1 | 173307901 | HGVM5380790 | 7.00E-11 | Imputed | 0.834 | 0.9601 |
| rs3130558 | 6 | 31097183 | HGVM1599176 | 7.43E-11 | Genotyped | 0.4493 | 1.16 |
| rs9263804 | 6 | 31135706 | HGVM8418301 | 7.48E-11 | Genotyped | 0.3441 | 1.212 |
| rs947474 | 10 | 6390450 | HGVM821043 | 8.00E-11 | Genotyped | 0.07715 | 0.6744 |
| rs72743477 | 15 | 67464291 | HGVM21660446 | 8.00E-11 | Imputed | 0.09734 | 1.404 |
| rs3821236 | 2 | 191902758 | HGVM2312422 | 8.00E-11 | Genotyped | 0.1711 | 1.325 |
| rs11580078 | 1 | 67669634 | HGVM4224173 | 8.00E-11 | Imputed | 0.2184 | 0.8108 |
| rs17885785 | 11 | 2167850 | HGVM11823709 | 8.00E-11 | Genotyped | 0.3845 | 0.8235 |
| rs36001488 | 2 | 234185267 | HGVM15135119 | 8.00E-11 | Imputed | 0.4284 | 1.141 |
| rs7100025 | 10 | 37592538 | HGVM7407714 | 8.00E-11 | Imputed | 0.4568 | 0.8769 |
| rs117372389 | 16 | 50668077 | HGVM32480762 | 8.00E-11 | Imputed | 0.4743 | 0.6112 |
| rs10822050 | 10 | 64438771 | HGVM3528131 | 8.00E-11 | Imputed | 0.5266 | 0.8935 |
| rs7725052 | 5 | 40487270 | HGVM7992057 | 8.00E-11 | Imputed | 0.5532 | 0.9101 |
| rs2066363 | 1 | 82237577 | HGVM812329 | 8.00E-11 | Imputed | 0.7038 | 0.937 |
| rs533646 | 11 | 118566746 | HGVM843154 | 8.00E-11 | Imputed | 0.7266 | 0.9406 |
| rs761357 | 6 | 135902599 | HGVM1789809 | 8.00E-11 | Imputed | 0.7316 | 1.064 |
| rs4625 | 3 | 49572140 | HGVM8692 | 8.00E-11 | Genotyped | 0.8714 | 1.032 |
| rs2736345 | 8 | 11352485 | HGVM1199550 | 8.00E-11 | Imputed | 0.8811 | 0.972 |
| rs1950897 | 14 | 68760141 | HGVM1956951 | 8.00E-11 | Imputed | 0.9658 | 0.9917 |
| rs2734573 | 6 | 31494738 | HGVM9964710 | 8.38E-11 | Genotyped | 0.01548 | 1.497 |
| rs62132293 | 19 | 838178 | HGVM14480473 | 9.00E-11 | Imputed | 0.1244 | 1.36 |
| rs849142 | 7 | 28185891 | HGVM300991 | 9.00E-11 | Genotyped | 0.142 | 0.7802 |
| rs1332099 | 10 | 101298451 | HGVM827282 | 9.00E-11 | Imputed | 0.1474 | 0.7706 |
| rs3803800 | 17 | 7462969 | HGVM2300285 | 9.00E-11 | Genotyped | 0.3718 | 1.2 |
| rs756699 | 5 | 133446575 | HGVM229942 | 9.00E-11 | Imputed | 0.5131 | 0.854 |
| rs231770 | 2 | 204729153 | HGVM988108 | 9.00E-11 | Imputed | 0.7145 | 1.064 |
| rs72946301 | 2 | 111861838 | HGVM22275587 | 9.00E-11 | Imputed | 0.8349 | 0.9457 |
| rs1061502 | 11 | 614318 | HGVM849748 | 9.00E-11 | Imputed | 0.8382 | 0.961 |
| rs3129877 | 6 | 32408597 | HGVM1598788 | 9.12E-11 | Genotyped | 0.6704 | 0.9203 |
| rs11539216 | 6 | 32917980 | HGVM4198564 | 9.95E-11 | Genotyped | 0.2441 | 0.6407 |
| rs9462027 | 6 | 34797241 | HGVM8578813 | 1.00E-10 | Genotyped | 0.04567 | 0.6791 |
| rs17119 | 6 | 14719496 | HGVM663310 | 1.00E-10 | Genotyped | 0.1852 | 0.7517 |
| rs2240339 | 1 | 17674108 | HGVM1238480 | 1.00E-10 | Genotyped | 0.2256 | 1.263 |
| rs4772201 | 13 | 100086259 | HGVM2747872 | 1.00E-10 | Genotyped | 0.3654 | 0.8196 |
| rs706015 | 7 | 27014988 | HGVM297078 | 1.00E-10 | Imputed | 0.3707 | 1.235 |
| rs3118470 | 10 | 6101713 | HGVM2237895 | 1.00E-10 | Genotyped | 0.4313 | 0.8615 |
| rs485497 | 3 | 159719132 | HGVM141916 | 1.00E-10 | Genotyped | 0.4634 | 0.8844 |
| rs12936409 | 17 | 38043649 | HGVM5581434 | 1.00E-10 | Imputed | 0.4738 | 1.134 |
| rs7196953 | 16 | 79649394 | HGVM7500855 | 1.00E-10 | Genotyped | 0.5028 | 1.129 |
| rs12785018 | 10 | 8515348 | HGVM5429704 | 1.00E-10 | Genotyped | 0.5175 | 1.12 |
| rs13277113 | 8 | 11349186 | HGVM5924738 | 1.00E-10 | Genotyped | 0.5397 | 0.8841 |
| rs212389 | 6 | 159489791 | HGVM247857 | 1.00E-10 | Imputed | 0.5842 | 0.9056 |
| rs773125 | 12 | 56394954 | HGVM869360 | 1.00E-10 | Imputed | 0.6072 | 0.9047 |
| rs7941765 | 11 | 128499000 | HGVM8203213 | 1.00E-10 | Genotyped | 0.6286 | 1.083 |
| rs9392504 | 6 | 412802 | HGVM8524587 | 1.00E-10 | Imputed | 0.6824 | 0.9364 |
| rs411079 | 17 | 68291371 | HGVM482752 | 1.00E-10 | Imputed | 0.7195 | 1.077 |
| rs941576 | 14 | 101306045 | HGVM1806224 | 1.00E-10 | Imputed | 0.7528 | 0.9451 |
| rs4252134 | 6 | 161153527 | HGVM2460693 | 1.00E-10 | Imputed | 0.7536 | 1.063 |
| rs4594881 | 5 | 35846815 | HGVM6359957 | 1.00E-10 | Imputed | 0.7765 | 1.052 |
| rs145268310 | 3 | 12310773 | HGVM51014381 | 1.00E-10 | Imputed | 0.8324 | 0.9521 |
| rs564976 | 3 | 159729059 | HGVM589140 | 1.00E-10 | Imputed | 0.8474 | 1.034 |
| rs2275247 | 1 | 35908451 | HGVM1240734 | 1.00E-10 | Imputed | 0.8608 | 1.061 |
| rs268134 | 2 | 65608363 | HGVM103100 | 1.00E-10 | Genotyped | 0.8851 | 1.03 |
| rs1843938 | 7 | 3113034 | HGVM1925314 | 1.00E-10 | Genotyped | 0.9017 | 0.9804 |
| rs4731532 | 7 | 128572766 | HGVM2717243 | 1.00E-10 | Imputed | 0.9108 | 0.9806 |
| rs3131009 | 6 | 31098832 | HGVM1599441 | 1.26E-10 | Genotyped | 0.4602 | 1.153 |
| rs9296073 | 6 | 33042551 | HGVM8439637 | 1.30E-10 | Genotyped | 0.5259 | 1.358 |
| rs1431403 | 6 | 33047031 | HGVM284679 | 1.56E-10 | Genotyped | 0.3147 | 0.8208 |
| rs3077 | 6 | 33033022 | HGVM222 | 1.77E-10 | Genotyped | 0.2795 | 0.7735 |
| rs6467970 | 7 | 83502255 | HGVM6842222 | 1.97E-10 | Genotyped | 0.699 | 0.9133 |
| rs761426 | 1 | 17413899 | HGVM769094 | 2.00E-10 | Imputed | 3.36E-04 | 2.138 |
| rs10201872 | 2 | 231106724 | HGVM3070911 | 2.00E-10 | Imputed | 9.97E-03 | 1.857 |
| rs729302 | 7 | 128568960 | HGVM297611 | 2.00E-10 | Genotyped | 0.1624 | 0.7675 |
| rs9828629 | 3 | 71530346 | HGVM8818976 | 2.00E-10 | Imputed | 0.2994 | 0.8336 |
| rs2050568 | 1 | 157770241 | HGVM811787 | 2.00E-10 | Imputed | 0.3098 | 0.8511 |
| rs6984496 | 8 | 10796093 | HGVM7301945 | 2.00E-10 | Genotyped | 0.342 | 1.182 |
| rs755374 | 5 | 158829294 | HGVM646910 | 2.00E-10 | Imputed | 0.347 | 1.186 |
| rs9277378 | 6 | 33050279 | HGVM8423357 | 2.00E-10 | Genotyped | 0.4055 | 0.8492 |
| rs827637 | 10 | 9010624 | HGVM354190 | 2.00E-10 | Genotyped | 0.4396 | 1.143 |
| rs9657904 | 3 | 105586714 | HGVM8737078 | 2.00E-10 | Imputed | 0.4675 | 1.172 |
| rs7929541 | 11 | 633689 | HGVM8191058 | 2.00E-10 | Imputed | 0.5232 | 0.8789 |
| rs3757727 | 7 | 20411897 | HGVM2269747 | 2.00E-10 | Imputed | 0.5502 | 0.9022 |
| rs1364229 | 16 | 62628775 | HGVM476295 | 2.00E-10 | Imputed | 0.6258 | 1.141 |
| rs2119704 | 14 | 88487689 | HGVM1365923 | 2.00E-10 | Genotyped | 0.6803 | 1.167 |
| rs2395148 | 6 | 32321554 | HGVM1126749 | 2.00E-10 | Genotyped | 0.7065 | 0.8237 |
| rs11785995 | 8 | 130952111 | HGVM4430567 | 2.00E-10 | Genotyped | 0.7526 | 1.116 |
| rs114357009 | 6 | 28965512 | HGVM35434644 | 2.00E-10 | Imputed | 0.8235 | 1.114 |
| rs10048743 | 2 | 213890232 | HGVM2942389 | 2.00E-10 | Imputed | 0.9193 | 1.024 |
| rs505922 | 9 | 136149229 | HGVM729310 | 2.00E-10 | Imputed | 0.9261 | 1.017 |
| rs3135021 | 6 | 33045558 | HGVM1600876 | 2.22E-10 | Genotyped | 0.8006 | 0.9572 |
| rs9277542 | 6 | 33055247 | HGVM8423518 | 2.40E-10 | Genotyped | 0.3461 | 0.8309 |
| rs6457710 | 6 | 33029437 | HGVM6832603 | 2.52E-10 | Genotyped | 0.8951 | 0.977 |
| rs2068204 | 6 | 33058718 | HGVM1119166 | 2.79E-10 | Genotyped | 0.7189 | 0.8064 |
| rs9277463 | 6 | 33053307 | HGVM8423440 | 2.93E-10 | Genotyped | 0.3461 | 0.8309 |
| rs6664969 | 1 | 2534978 | HGVM6997911 | 3.00E-10 | Imputed | 0.03171 | 1.496 |
| rs17466626 | 12 | 40760631 | HGVM11320170 | 3.00E-10 | Imputed | 0.03958 | 8.773 |
| rs11085725 | 19 | 10462513 | HGVM3793313 | 3.00E-10 | Imputed | 0.096 | 1.423 |
| rs4958881 | 5 | 150450236 | HGVM2882770 | 3.00E-10 | Genotyped | 0.2126 | 1.359 |
| rs9663421 | 10 | 6055604 | HGVM8740652 | 3.00E-10 | Genotyped | 0.3807 | 0.8448 |
| rs10492972 | 1 | 10353112 | HGVM3216390 | 3.00E-10 | Imputed | 0.4491 | 1.152 |
| rs801429 | 17 | 37435378 | HGVM486674 | 3.00E-10 | Imputed | 0.4832 | 1.161 |
| rs2253717 | 17 | 38868236 | HGVM1413866 | 3.00E-10 | Imputed | 0.4851 | 0.8762 |
| rs2298428 | 22 | 21982892 | HGVM1482715 | 3.00E-10 | Imputed | 0.5157 | 1.133 |
| rs6043409 | 20 | 1616206 | HGVM6694224 | 3.00E-10 | Genotyped | 0.542 | 1.117 |
| rs744166 | 17 | 40514201 | HGVM486148 | 3.00E-10 | Imputed | 0.5757 | 0.9101 |
| rs6662618 | 1 | 92935411 | HGVM6995703 | 3.00E-10 | Genotyped | 0.694 | 1.085 |
| rs657075 | 5 | 131430118 | HGVM227587 | 3.00E-10 | Imputed | 0.6946 | 0.8901 |
| rs2278600 | 5 | 71756670 | HGVM1100060 | 3.00E-10 | Genotyped | 0.7021 | 1.094 |
| rs917116 | 7 | 28172739 | HGVM302603 | 3.00E-10 | Imputed | 0.7859 | 0.9455 |
| rs6914622 | 6 | 148514301 | HGVM7235637 | 3.00E-10 | Imputed | 0.7963 | 1.047 |
| rs11581062 | 1 | 101407519 | HGVM4225158 | 3.00E-10 | Genotyped | 0.8482 | 0.9631 |
| rs805297 | 6 | 31622606 | HGVM269803 | 3.00E-10 | Genotyped | 0.9393 | 0.9873 |
| rs4963128 | 11 | 589564 | HGVM2885799 | 3.00E-10 | Genotyped | 0.9435 | 0.9872 |
| rs12711490 | 16 | 85973028 | HGVM5356472 | 3.00E-10 | Imputed | 0.9897 | 0.9974 |
| rs3128966 | 6 | 33055946 | HGVM1598442 | 3.06E-10 | Genotyped | 0.8542 | 0.9585 |
| rs3128965 | 6 | 33055899 | HGVM1598441 | 3.08E-10 | Genotyped | 0.8542 | 0.9585 |
| rs2179920 | 6 | 33058874 | HGVM1526107 | 3.11E-10 | Genotyped | 0.4598 | 0.8583 |
| rs3117229 | 6 | 33056069 | HGVM1596418 | 3.15E-10 | Genotyped | 0.6586 | 0.9079 |
| rs3128963 | 6 | 33055780 | HGVM1598439 | 3.62E-10 | Genotyped | 0.3461 | 0.8309 |
| rs367645 | 6 | 33024499 | HGVM253124 | 3.70E-10 | Genotyped | 0.8004 | 0.8703 |
| rs10516487 | 4 | 102751076 | HGVM3239546 | 3.74E-10 | Imputed | 0.267 | 0.8126 |
| rs6689470 | 1 | 92951210 | HGVM7021001 | 3.93E-10 | Imputed | 0.7113 | 0.9176 |
| rs3765209 | 21 | 16789349 | HGVM2274449 | 4.00E-10 | Imputed | 0.2678 | 1.238 |
| rs4780346 | 16 | 11288806 | HGVM2754166 | 4.00E-10 | Imputed | 0.2697 | 0.794 |
| rs72727394 | 15 | 38847022 | HGVM21657593 | 4.00E-10 | Imputed | 0.4085 | 0.8499 |
| rs193778 | 16 | 11351211 | HGVM467259 | 4.00E-10 | Imputed | 0.4696 | 1.159 |
| rs5986948 | X | 153266172 | HGVM6643306 | 4.00E-10 | Imputed | 0.4773 | 0.8545 |
| rs10478040 | 5 | 110590399 | HGVM3204323 | 4.00E-10 | Imputed | 0.4985 | 1.303 |
| rs4227 | 17 | 7491177 | HGVM3698 | 4.00E-10 | Genotyped | 0.5275 | 0.8958 |
| rs2980512 | 8 | 8140901 | HGVM1572504 | 4.00E-10 | Imputed | 0.6259 | 1.091 |
| rs17830558 | 2 | 160878364 | HGVM14973611 | 4.00E-10 | Genotyped | 0.69 | 1.071 |
| rs4388254 | 5 | 133428601 | HGVM2526520 | 4.00E-10 | Imputed | 0.6989 | 0.8898 |
| rs7836059 | 8 | 11272164 | HGVM8101439 | 4.00E-10 | Genotyped | 0.6994 | 0.9332 |
| rs11785816 | 8 | 129531186 | HGVM4430388 | 4.00E-10 | Imputed | 0.7274 | 1.069 |
| rs941816 | 6 | 36375304 | HGVM8544283 | 4.00E-10 | Genotyped | 0.7338 | 0.9308 |
| rs951005 | 9 | 34743681 | HGVM733202 | 4.00E-10 | Imputed | 0.9713 | 0.9924 |
| rs9979383 | 21 | 36715761 | HGVM8949969 | 5.00E-10 | Genotyped | 0.118 | 0.7575 |
| rs3099844 | 6 | 31448976 | HGVM2234808 | 5.00E-10 | Genotyped | 0.4023 | 0.8042 |
| rs759648 | 8 | 129158945 | HGVM320759 | 5.00E-10 | Genotyped | 0.7102 | 1.069 |
| rs887369 | X | 30577846 | HGVM577294 | 5.00E-10 | Imputed | 0.7279 | 0.9263 |
| rs934734 | 2 | 65595586 | HGVM1695357 | 5.00E-10 | Imputed | 0.7446 | 1.056 |
| rs3807306 | 7 | 128580680 | HGVM2302657 | 5.00E-10 | Genotyped | 0.8269 | 0.9628 |
| rs3024505 | 1 | 206939904 | HGVM1632435 | 5.00E-10 | Genotyped | 0.8607 | 1.04 |
| rs3007421 | 1 | 6530189 | HGVM2228801 | 5.00E-10 | Imputed | 0.8766 | 0.9626 |
| rs2073045 | 6 | 32339548 | HGVM1119250 | 5.00E-10 | Genotyped | 0.9486 | 1.011 |
| rs9277194 | 6 | 33023894 | HGVM10388980 | 5.35E-10 | Genotyped | 0.5927 | 0.9158 |
| rs3781913 | 11 | 72373496 | HGVM2285620 | 6.00E-10 | Imputed | 0.02102 | 0.6777 |
| rs12575600 | 11 | 128324869 | HGVM5220285 | 6.00E-10 | Imputed | 0.1195 | 0.6243 |
| rs2955587 | 8 | 8098079 | HGVM1561610 | 6.00E-10 | Imputed | 0.39 | 0.8575 |
| rs72853903 | 11 | 2198665 | HGVM21167315 | 6.00E-10 | Imputed | 0.4195 | 1.166 |
| rs3813308 | 5 | 118690781 | HGVM2307042 | 6.00E-10 | Imputed | 0.7298 | 0.9431 |
| rs4902562 | 14 | 68731458 | HGVM2843356 | 6.00E-10 | Genotyped | 0.85 | 1.034 |
| rs12466022 | 2 | 43359061 | HGVM5110540 | 6.00E-10 | Genotyped | 0.8613 | 0.9675 |
| rs9277196 | 6 | 33023946 | HGVM10388981 | 6.48E-10 | Genotyped | 0.6297 | 1.084 |
| rs7763822 | 6 | 33060428 | HGVM8029935 | 6.93E-10 | Genotyped | 0.7085 | 0.7999 |
| rs9355610 | 6 | 167383075 | HGVM8492421 | 7.00E-10 | Genotyped | 0.02826 | 1.542 |
| rs1538171 | 6 | 126752884 | HGVM680394 | 7.00E-10 | Imputed | 0.4036 | 1.156 |
| rs10491033 | 10 | 86186241 | HGVM3214455 | 7.00E-10 | Genotyped | 0.4828 | 1.427 |
| rs2248932 | 8 | 11391650 | HGVM1180070 | 7.00E-10 | Genotyped | 0.5647 | 1.109 |
| rs10857635 | 10 | 49974656 | HGVM3563904 | 7.00E-10 | Imputed | 0.67 | 1.085 |
| rs72776098 | 10 | 6116254 | HGVM21024895 | 7.00E-10 | Imputed | 0.6828 | 1.311 |
| rs7634389 | 3 | 186738421 | HGVM7901564 | 7.00E-10 | Imputed | 0.7521 | 1.058 |
| rs1858036 | 2 | 65598241 | HGVM1010470 | 7.00E-10 | Imputed | 0.7847 | 0.9549 |
| rs72689399 | 1 | 167038121 | HGVM20847113 | 7.00E-10 | Imputed | 0.7892 | 0.8397 |
| rs7918084 | 10 | 94429467 | HGVM8179654 | 7.00E-10 | Imputed | 0.9652 | 1.008 |
| rs4936059 | 11 | 128502496 | HGVM2866335 | 7.00E-10 | Genotyped | 0.9967 | 1.001 |
| rs11783247 | 8 | 10788875 | HGVM4427817 | 8.00E-10 | Genotyped | 0.3518 | 1.179 |
| rs7909519 | 10 | 6089841 | HGVM8171143 | 8.00E-10 | Imputed | 0.3738 | 1.295 |
| rs3130190 | 6 | 33061690 | HGVM1598947 | 8.00E-10 | Genotyped | 0.4102 | 0.85 |
| rs9372120 | 6 | 106667535 | HGVM8506868 | 8.00E-10 | Imputed | 0.4714 | 1.177 |
| rs1530687 | 3 | 119114515 | HGVM164080 | 8.00E-10 | Imputed | 0.4938 | 1.124 |
| rs624988 | 1 | 117263790 | HGVM80996 | 8.00E-10 | Genotyped | 0.8411 | 1.035 |
| rs7523907 | 1 | 167427247 | HGVM7791234 | 9.00E-10 | Genotyped | 0.05845 | 0.7136 |
| rs6998967 | 8 | 81364205 | HGVM7315882 | 9.00E-10 | Genotyped | 0.08609 | 0.6949 |
| rs2469434 | 18 | 67544046 | HGVM10104260 | 9.00E-10 | Imputed | 0.3748 | 0.8516 |
| rs9826828 | 3 | 136402060 | HGVM10391926 | 9.00E-10 | Imputed | 0.8252 | 1.142 |
| rs12802200 | 11 | 566936 | HGVM5446931 | 9.00E-10 | Genotyped | 0.9435 | 1.016 |
| rs2681424 | 3 | 121769522 | HGVM1050727 | 9.51E-10 | Imputed | 0.7306 | 1.058 |
| rs7221109 | 17 | 38770286 | HGVM7524394 | 9.89E-10 | Genotyped | 0.2134 | 0.7985 |
| rs11129295 | 3 | 27788780 | HGVM3837192 | 1.00E-09 | Genotyped | 0.03608 | 0.6869 |
| rs6476839 | 9 | 4290823 | HGVM6850494 | 1.00E-09 | Imputed | 0.04613 | 0.7146 |
| rs8133843 | 21 | 36738242 | HGVM8388509 | 1.00E-09 | Imputed | 0.1326 | 0.7616 |
| rs7120737 | 11 | 47702395 | HGVM7427324 | 1.00E-09 | Imputed | 0.1493 | 0.6998 |
| rs301819 | 1 | 8501786 | HGVM70011 | 1.00E-09 | Imputed | 0.2632 | 0.8165 |
| rs11089637 | 22 | 21979096 | HGVM3797252 | 1.00E-09 | Genotyped | 0.3783 | 1.196 |
| rs2033562 | 8 | 103547739 | HGVM1176284 | 1.00E-09 | Imputed | 0.4031 | 1.155 |
| rs2844482 | 6 | 31539767 | HGVM1141294 | 1.00E-09 | Genotyped | 0.4755 | 1.161 |
| rs9292777 | 5 | 40437948 | HGVM8436383 | 1.00E-09 | Genotyped | 0.5494 | 1.114 |
| rs3781094 | 10 | 8101427 | HGVM2285087 | 1.00E-09 | Imputed | 0.5537 | 0.9026 |
| rs10175798 | 2 | 30449594 | HGVM3048471 | 1.00E-09 | Imputed | 0.5891 | 0.9054 |
| rs2292327 | 16 | 84030652 | HGVM2066438 | 1.00E-09 | Genotyped | 0.6419 | 1.082 |
| rs12129787 | 1 | 161492587 | HGVM4774697 | 1.00E-09 | Genotyped | 0.6431 | 1.088 |
| rs12253981 | 10 | 6092346 | HGVM4898578 | 1.00E-09 | Imputed | 0.6847 | 0.934 |
| rs763361 | 18 | 67531642 | HGVM504751 | 1.00E-09 | Genotyped | 0.7128 | 0.9391 |
| rs4077515 | 9 | 139266496 | HGVM2417852 | 1.00E-09 | Genotyped | 0.7227 | 0.9409 |
| rs5754100 | 22 | 21916166 | HGVM6441110 | 1.00E-09 | Imputed | 0.7305 | 1.068 |
| rs115829748 | 5 | 71394387 | HGVM35131687 | 1.00E-09 | Imputed | 0.7383 | 0.8055 |
| rs773107 | 12 | 56369506 | HGVM869344 | 1.00E-09 | Imputed | 0.7679 | 0.944 |
| rs7579944 | 2 | 30445026 | HGVM7846936 | 1.00E-09 | Genotyped | 0.7684 | 0.9484 |
| rs72833417 | 17 | 45873049 | HGVM21868238 | 1.00E-09 | Imputed | 0.7746 | 0.9249 |
| rs10086568 | 8 | 6900336 | HGVM2973085 | 1.00E-09 | Imputed | 0.7779 | 1.054 |
| rs7819602 | 8 | 10726842 | HGVM8085025 | 1.00E-09 | Imputed | 0.8549 | 0.9684 |
| rs6840978 | 4 | 123554707 | HGVM7165819 | 1.00E-09 | Genotyped | 0.9501 | 0.9854 |
| rs6894249 | 5 | 131797547 | HGVM7216611 | 1.00E-09 | Imputed | 0.9608 | 0.9917 |
| rs12467084 | 2 | 37948570 | HGVM5111604 | 1.00E-09 | Imputed | 0.9633 | 0.9814 |
| rs9782955 | 1 | 236039877 | HGVM8787144 | 1.00E-09 | Genotyped | 0.9938 | 0.9984 |
| rs8179673 | 2 | 191969341 | HGVM8399033 | 1.31E-09 | Imputed | 0.2236 | 1.259 |
| rs9296069 | 6 | 33018957 | HGVM8439633 | 1.46E-09 | Imputed | 0.01397 | 1.611 |
| rs9271642 | 6 | 32592219 | HGVM11224906 | 1.70E-09 | Imputed | 0.1999 | 1.265 |
| rs11203203 | 21 | 43836186 | HGVM3911364 | 1.74E-09 | Genotyped | 0.5524 | 1.11 |
| rs7020673 | 9 | 4291747 | HGVM7336783 | 1.88E-09 | Imputed | 0.1276 | 1.303 |
| rs6596147 | 5 | 133047775 | HGVM6953385 | 2.00E-09 | Genotyped | 2.90E-03 | 2.028 |
| rs10499197 | 6 | 138132516 | HGVM3222616 | 2.00E-09 | Imputed | 3.56E-03 | 0.1851 |
| rs7088058 | 10 | 124149352 | HGVM7396218 | 2.00E-09 | Imputed | 7.57E-03 | 1.594 |
| rs440841 | 6 | 33019643 | HGVM9966244 | 2.00E-09 | Genotyped | 0.01015 | 0.627 |
| rs7137828 | 12 | 111932800 | HGVM10052663 | 2.00E-09 | Imputed | 0.01485 | 0.6481 |
| rs8042861 | 15 | 90977333 | HGVM8303923 | 2.00E-09 | Imputed | 0.0364 | 0.6869 |
| rs72634030 | 17 | 5272580 | HGVM21863143 | 2.00E-09 | Imputed | 0.2552 | 1.41 |
| rs2456449 | 8 | 128192981 | HGVM1185881 | 2.00E-09 | Imputed | 0.2561 | 0.823 |
| rs7151526 | 14 | 94863636 | HGVM7456838 | 2.00E-09 | Imputed | 0.2615 | 1.714 |
| rs5865 | 2 | 98373006 | HGVM8703 | 2.00E-09 | Genotyped | 0.2707 | 0.83 |
| rs12964116 | 18 | 61442619 | HGVM5609198 | 2.00E-09 | Genotyped | 0.2996 | 1.701 |
| rs1422673 | 5 | 150438988 | HGVM240654 | 2.00E-09 | Genotyped | 0.3396 | 1.212 |
| rs11741255 | 5 | 131811182 | HGVM4385780 | 2.00E-09 | Imputed | 0.3871 | 1.156 |
| rs2071543 | 6 | 32811629 | HGVM572171 | 2.00E-09 | Genotyped | 0.389 | 1.23 |
| rs2178077 | 12 | 131380473 | HGVM1337289 | 2.00E-09 | Imputed | 0.4523 | 1.222 |
| rs2045258 | 6 | 126674354 | HGVM691315 | 2.00E-09 | Genotyped | 0.4569 | 1.139 |
| rs3828069 | 1 | 67839573 | HGVM2316987 | 2.00E-09 | Imputed | 0.4618 | 1.18 |
| rs4679081 | 3 | 33013483 | HGVM9874053 | 2.00E-09 | Genotyped | 0.607 | 0.9151 |
| rs3129891 | 6 | 32415080 | HGVM9694040 | 2.00E-09 | Imputed | 0.6348 | 1.122 |
| rs12140275 | 1 | 38633879 | HGVM4785203 | 2.00E-09 | Imputed | 0.6472 | 1.098 |
| rs1748041 | 1 | 17655407 | HGVM1229999 | 2.00E-09 | Genotyped | 0.6879 | 1.076 |
| rs6715284 | 2 | 202154397 | HGVM7045601 | 2.00E-09 | Imputed | 0.7031 | 1.102 |
| rs6793295 | 3 | 169518455 | HGVM7120312 | 2.00E-09 | Imputed | 0.7816 | 1.055 |
| rs694739 | 11 | 64097233 | HGVM388123 | 2.00E-09 | Imputed | 0.8008 | 0.9548 |
| rs564799 | 3 | 159728987 | HGVM143314 | 2.00E-09 | Genotyped | 0.8474 | 1.034 |
| rs13069553 | 3 | 169508272 | HGVM5715244 | 2.00E-09 | Genotyped | 0.8887 | 1.028 |
| rs4705959 | 5 | 131865791 | HGVM6368155 | 2.00E-09 | Imputed | 0.965 | 1.009 |
| rs2234067 | 6 | 36355654 | HGVM2052664 | 2.00E-09 | Imputed | 0.9958 | 1.001 |
| rs3130578 | 6 | 33018310 | HGVM9780406 | 2.21E-09 | Imputed | 0.5363 | 0.8741 |
| rs4925166 | 17 | 18210810 | HGVM6382717 | 2.69E-09 | Imputed | 0.1876 | 1.263 |
| rs34350562 | 7 | 128718708 | HGVM20237097 | 2.87E-09 | Imputed | 9.15E-03 | 0.5152 |
| rs9516053 | 13 | 92945884 | HGVM10268591 | 3.00E-09 | Imputed | 0.04292 | 0.7044 |
| rs61828284 | 1 | 173299743 | HGVM19785979 | 3.00E-09 | Imputed | 0.07113 | 0.5289 |
| rs2286672 | 17 | 4712617 | HGVM1414661 | 3.00E-09 | Imputed | 0.08517 | 0.5985 |
| rs2390314 | 7 | 20455978 | HGVM2105519 | 3.00E-09 | Imputed | 0.08648 | 1.812 |
| rs865488 | 8 | 133938622 | HGVM714007 | 3.00E-09 | Imputed | 0.1337 | 1.349 |
| rs2228145 | 1 | 154426970 | HGVM6147818 | 3.00E-09 | Imputed | 0.1373 | 0.761 |
| rs72632736 | 1 | 4449204 | HGVM20836050 | 3.00E-09 | Imputed | 0.1785 | 0.5876 |
| rs7769192 | 6 | 137962655 | HGVM8035112 | 3.00E-09 | Imputed | 0.185 | 1.255 |
| rs62375550 | 5 | 110387125 | HGVM17442712 | 3.00E-09 | Imputed | 0.2221 | 2.709 |
| rs74830391 | 7 | 37427289 | HGVM28969129 | 3.00E-09 | Imputed | 0.2649 | 0.723 |
| rs7238078 | 18 | 56384192 | HGVM7540915 | 3.00E-09 | Genotyped | 0.4414 | 0.8546 |
| rs11675342 | 2 | 1407628 | HGVM4319731 | 3.00E-09 | Imputed | 0.5061 | 0.8926 |
| rs2872507 | 17 | 38040763 | HGVM1721274 | 3.00E-09 | Genotyped | 0.5113 | 1.122 |
| rs4665719 | 2 | 25017860 | HGVM2668769 | 3.00E-09 | Imputed | 0.6125 | 0.9099 |
| rs12946510 | 17 | 37912377 | HGVM5591549 | 3.00E-09 | Imputed | 0.6215 | 1.09 |
| rs2671692 | 10 | 50097819 | HGVM1294175 | 3.00E-09 | Genotyped | 0.6293 | 1.09 |
| rs2072438 | 9 | 123651301 | HGVM573071 | 3.00E-09 | Imputed | 0.6479 | 1.083 |
| rs6752643 | 2 | 199123727 | HGVM7081515 | 3.00E-09 | Imputed | 0.7214 | 1.117 |
| rs9373594 | 6 | 149834574 | HGVM8508194 | 3.00E-09 | Imputed | 0.7594 | 0.9314 |
| rs11126034 | 2 | 65580221 | HGVM3833916 | 3.00E-09 | Imputed | 0.7847 | 0.9549 |
| rs3824660 | 10 | 8104722 | HGVM2314725 | 3.00E-09 | Imputed | 0.851 | 1.032 |
| rs2315646 | 20 | 62379750 | HGVM2072984 | 3.00E-09 | Imputed | 0.8762 | 1.028 |
| rs227163 | 1 | 7961206 | HGVM67326 | 3.00E-09 | Genotyped | 0.9324 | 0.9852 |
| rs4149759 | X | 138635054 | HGVM2448326 | 3.51E-09 | Genotyped | 0.8141 | 1.091 |
| rs10774625 | 12 | 111910219 | HGVM3480377 | 4.00E-09 | Genotyped | 4.21E-03 | 0.6029 |
| rs6886392 | 5 | 100135865 | HGVM7209118 | 4.00E-09 | Imputed | 0.0838 | 1.395 |
| rs4602043 | 16 | 5588689 | HGVM6360505 | 4.00E-09 | Imputed | 0.1364 | 0.7401 |
| rs512244 | 4 | 13098882 | HGVM174813 | 4.00E-09 | Genotyped | 0.1925 | 0.7219 |
| rs11587876 | 1 | 85915183 | HGVM4231996 | 4.00E-09 | Imputed | 0.2259 | 1.284 |
| rs1036207 | 5 | 141499041 | HGVM3141290 | 4.00E-09 | Imputed | 0.4125 | 1.154 |
| rs2305480 | 17 | 38062196 | HGVM1415181 | 4.00E-09 | Genotyped | 0.4436 | 1.144 |
| rs10912267 | 1 | 172674776 | HGVM3618802 | 4.00E-09 | Imputed | 0.5025 | 0.8459 |
| rs478222 | 2 | 25301755 | HGVM109686 | 4.00E-09 | Imputed | 0.606 | 0.9125 |
| rs2074038 | 11 | 44087989 | HGVM568535 | 4.00E-09 | Imputed | 0.6755 | 0.8985 |
| rs2075876 | 21 | 45709153 | HGVM1456700 | 4.00E-09 | Imputed | 0.6796 | 0.8841 |
| rs1689510 | 12 | 56396768 | HGVM881677 | 4.00E-09 | Imputed | 0.6893 | 0.9231 |
| rs2256814 | 20 | 62373983 | HGVM2056637 | 4.00E-09 | Imputed | 0.8636 | 1.035 |
| rs17824933 | 11 | 60760612 | HGVM11822741 | 4.00E-09 | Imputed | 0.8638 | 0.9643 |
| rs3129716 | 6 | 32657436 | HGVM1598699 | 4.00E-09 | Genotyped | 0.898 | 1.035 |
| rs67250450 | 7 | 28174986 | HGVM23403455 | 4.00E-09 | Imputed | 0.9025 | 1.026 |
| rs147859879 | 5 | 1729788 | HGVM54453759 | 4.00E-09 | Imputed | 0.9475 | 1.042 |
| rs17445836 | 16 | 86017663 | HGVM13490466 | 4.00E-09 | Imputed | 0.9933 | 0.9982 |
| rs4364506 | 6 | 130389940 | HGVM2515094 | 4.06E-09 | Imputed | 0.7955 | 0.9496 |
| rs151227 | 16 | 28549508 | HGVM466158 | 4.83E-09 | Genotyped | 0.9881 | 1.004 |
| rs72827176 | 17 | 37810795 | HGVM21866881 | 5.00E-09 | Imputed | 0.04031 | 0.2856 |
| rs8129030 | 21 | 36712588 | HGVM8383794 | 5.00E-09 | Imputed | 0.1444 | 0.772 |
| rs13380830 | 17 | 40297658 | HGVM6029054 | 5.00E-09 | Imputed | 0.199 | 0.7574 |
| rs1456988 | 14 | 98488007 | HGVM450820 | 5.00E-09 | Genotyped | 0.3521 | 1.182 |
| rs10849448 | 12 | 6493351 | HGVM3555673 | 5.00E-09 | Imputed | 0.4088 | 0.8378 |
| rs79337446 | 3 | 33088785 | HGVM27742895 | 5.00E-09 | Imputed | 0.4349 | 1.39 |
| rs1217393 | 1 | 114433946 | HGVM783912 | 5.00E-09 | Imputed | 0.5562 | 1.118 |
| rs2303759 | 19 | 49869051 | HGVM1442631 | 5.00E-09 | Genotyped | 0.5845 | 1.118 |
| rs16947122 | 12 | 117366531 | HGVM12221555 | 5.00E-09 | Imputed | 0.5994 | 1.48 |
| rs3125734 | 10 | 63958112 | HGVM2240047 | 5.00E-09 | Genotyped | 0.6203 | 0.9115 |
| rs2019960 | 8 | 129192271 | HGVM1176211 | 5.00E-09 | Genotyped | 0.6338 | 1.101 |
| rs4272 | 7 | 92236829 | HGVM63867 | 5.00E-09 | Genotyped | 0.6401 | 0.9059 |
| rs128738 | 5 | 131540875 | HGVM199126 | 5.00E-09 | Genotyped | 0.6627 | 0.9035 |
| rs2425752 | 20 | 44702120 | HGVM2121029 | 5.00E-09 | Imputed | 0.6705 | 1.091 |
| rs36023980 | 17 | 73341284 | HGVM13849478 | 5.00E-09 | Imputed | 0.7043 | 1.085 |
| rs5954596 | X | 141360571 | HGVM10271827 | 5.00E-09 | Imputed | 0.7224 | 1.102 |
| rs9603618 | 13 | 40371377 | HGVM8699572 | 5.00E-09 | Genotyped | 0.7559 | 1.061 |
| rs10069690 | 5 | 1279790 | HGVM2959443 | 5.00E-09 | Genotyped | 0.8459 | 1.039 |
| rs2663052 | 10 | 50069395 | HGVM9719961 | 5.00E-09 | Genotyped | 0.8669 | 1.028 |
| rs354033 | 7 | 149289464 | HGVM292823 | 5.00E-09 | Genotyped | 0.9681 | 0.9915 |
| rs7829816 | 8 | 56849386 | HGVM8095212 | 5.40E-09 | Genotyped | 0.3631 | 1.233 |
| rs16867384 | 2 | 182111206 | HGVM14948771 | 6.00E-09 | Genotyped | 0.08799 | 0.6995 |
| rs12601925 | 17 | 4503195 | HGVM5246668 | 6.00E-09 | Genotyped | 0.1438 | 0.7198 |
| rs2240336 | 1 | 17674402 | HGVM1238477 | 6.00E-09 | Imputed | 0.1696 | 1.291 |
| rs494003 | 11 | 65542298 | HGVM842388 | 6.00E-09 | Genotyped | 0.4375 | 1.195 |
| rs2900180 | 9 | 123706382 | HGVM1546683 | 6.00E-09 | Imputed | 0.4709 | 0.8788 |
| rs143123127 | 17 | 38007190 | HGVM45671949 | 6.00E-09 | Imputed | 0.5148 | 1.331 |
| rs77150043 | 16 | 50304249 | HGVM26116986 | 6.00E-09 | Imputed | 0.6323 | 0.9023 |
| rs533259 | 1 | 182549019 | HGVM77353 | 6.00E-09 | Genotyped | 0.6557 | 0.8463 |
| rs1150753 | 6 | 32059867 | HGVM276679 | 6.00E-09 | Genotyped | 0.6988 | 0.8939 |
| rs2266959 | 22 | 21922904 | HGVM1482177 | 6.00E-09 | Imputed | 0.7464 | 1.065 |
| rs231735 | 2 | 204693876 | HGVM988075 | 6.00E-09 | Imputed | 0.7488 | 1.053 |
| rs7780389 | 7 | 51015193 | HGVM8046029 | 6.00E-09 | Imputed | 0.824 | 1.093 |
| rs802734 | 6 | 128278798 | HGVM1792185 | 6.00E-09 | Genotyped | 0.9633 | 0.9915 |
| rs62323881 | 4 | 123038295 | HGVM16899652 | 6.00E-09 | Imputed | 0.9735 | 0.9889 |
| rs10279821 | 7 | 128683547 | HGVM3134146 | 6.50E-09 | Genotyped | 0.31 | 0.8301 |
| rs12982646 | 19 | 499978 | HGVM5627776 | 6.53E-09 | Genotyped | 0.8734 | 1.03 |
| rs4240671 | 8 | 10767748 | HGVM2455269 | 6.60E-09 | Genotyped | 0.5164 | 0.8931 |
| rs10509540 | 10 | 90023033 | HGVM3232606 | 6.92E-09 | Genotyped | 0.04328 | 0.6644 |
| rs3731714 | 2 | 202060820 | HGVM2252273 | 7.00E-09 | Genotyped | 0.04775 | 0.6892 |
| rs1043099 | 22 | 30681257 | HGVM556940 | 7.00E-09 | Imputed | 0.2103 | 0.7493 |
| rs1182531 | 20 | 58393002 | HGVM530975 | 7.00E-09 | Genotyped | 0.2607 | 0.7721 |
| rs998731 | 8 | 81095395 | HGVM1815508 | 7.00E-09 | Imputed | 0.2741 | 1.216 |
| rs1439112 | 2 | 135062598 | HGVM128184 | 7.00E-09 | Genotyped | 0.3762 | 0.8517 |
| rs17019602 | 1 | 108188858 | HGVM19088740 | 7.00E-09 | Genotyped | 0.5984 | 0.8909 |
| rs2618444 | 8 | 11338370 | HGVM1193064 | 7.00E-09 | Imputed | 0.6226 | 0.9064 |
| rs1790588 | 18 | 67535184 | HGVM974520 | 7.00E-09 | Genotyped | 0.6355 | 0.9204 |
| rs27293 | 5 | 96357178 | HGVM194957 | 7.00E-09 | Imputed | 0.6844 | 1.072 |
| rs678347 | 8 | 102463602 | HGVM319511 | 7.00E-09 | Imputed | 0.868 | 1.032 |
| rs229533 | 22 | 37587111 | HGVM557514 | 7.00E-09 | Imputed | 0.8699 | 1.028 |
| rs8087252 | 18 | 46041755 | HGVM8348037 | 7.00E-09 | Genotyped | 0.9916 | 1.003 |
| rs6445975 | 3 | 58370177 | HGVM6822340 | 7.10E-09 | Genotyped | 0.1049 | 1.341 |
| rs41476751 | 16 | 31335906 | HGVM13569448 | 7.84E-09 | Imputed | 0.7371 | 1.079 |
| rs340630 | 4 | 87958395 | HGVM170449 | 8.00E-09 | Imputed | 0.07448 | 0.737 |
| rs2294025 | 8 | 134145512 | HGVM1181162 | 8.00E-09 | Imputed | 0.2752 | 0.8269 |
| rs924043 | 6 | 170379025 | HGVM1115211 | 8.00E-09 | Imputed | 0.3141 | 0.8036 |
| rs11170445 | 12 | 53545692 | HGVM3878502 | 8.00E-09 | Imputed | 0.3837 | 1.179 |
| rs877819 | 10 | 50042951 | HGVM1271778 | 8.00E-09 | Imputed | 0.4303 | 0.8657 |
| rs10946940 | 6 | 27560587 | HGVM3653671 | 8.00E-09 | Genotyped | 0.5534 | 1.11 |
| rs10275421 | 7 | 100943061 | HGVM3130582 | 8.00E-09 | Imputed | 0.58 | 0.8519 |
| rs2941509 | 17 | 37921194 | HGVM1623495 | 8.00E-09 | Genotyped | 0.6477 | 1.215 |
| rs11059919 | 12 | 129289190 | HGVM3767287 | 8.00E-09 | Imputed | 0.7584 | 0.9441 |
| rs6651252 | 8 | 129567181 | HGVM6986367 | 8.00E-09 | Genotyped | 0.8293 | 1.057 |
| rs4916342 | 1 | 173347837 | HGVM6382107 | 8.00E-09 | Imputed | 0.9075 | 1.021 |
| rs9746695 | 16 | 11207894 | HGVM8774933 | 8.19E-09 | Genotyped | 0.6202 | 1.093 |
| rs6732565 | 2 | 111607832 | HGVM7062172 | 9.00E-09 | Imputed | 4.94E-03 | 0.6171 |
| rs771767 | 3 | 101748638 | HGVM147889 | 9.00E-09 | Genotyped | 0.04358 | 0.6704 |
| rs3806624 | 3 | 27764623 | HGVM2302152 | 9.00E-09 | Genotyped | 0.1264 | 1.312 |
| rs4780401 | 16 | 11839326 | HGVM2754212 | 9.00E-09 | Imputed | 0.3307 | 1.2 |
| rs726288 | 10 | 81706973 | HGVM817200 | 9.00E-09 | Imputed | 0.8955 | 1.062 |
| rs2812197 | 13 | 50817826 | HGVM9946354 | 9.95E-09 | Imputed | 0.2455 | 1.235 |
| rs7665090 | 4 | 103551603 | HGVM7932291 | 1.00E-08 | Imputed | 9.27E-03 | 0.6232 |
| rs11755724 | 6 | 7118990 | HGVM4400257 | 1.00E-08 | Genotyped | 0.01851 | 1.528 |
| rs28583049 | 3 | 108443415 | HGVM15708646 | 1.00E-08 | Imputed | 0.02495 | 1.684 |
| rs223889 | 16 | 57392241 | HGVM468126 | 1.00E-08 | Imputed | 0.02713 | 1.518 |
| rs1398607 | 3 | 101755738 | HGVM159422 | 1.00E-08 | Genotyped | 0.03651 | 0.6401 |
| rs17174870 | 2 | 112665201 | HGVM14962181 | 1.00E-08 | Genotyped | 0.04882 | 0.6885 |
| rs353592 | 11 | 35119482 | HGVM366567 | 1.00E-08 | Imputed | 0.05183 | 1.406 |
| rs12519788 | 5 | 30405068 | HGVM5164385 | 1.00E-08 | Imputed | 0.07176 | 0.7241 |
| rs13344313 | 19 | 18517767 | HGVM5992347 | 1.00E-08 | Genotyped | 0.0733 | 1.429 |
| rs1250563 | 10 | 81047383 | HGVM825570 | 1.00E-08 | Imputed | 0.1049 | 1.353 |
| rs820077 | 6 | 35033854 | HGVM270551 | 1.00E-08 | Genotyped | 0.112 | 0.7037 |
| rs12453507 | 17 | 38053207 | HGVM5098004 | 1.00E-08 | Imputed | 0.1897 | 1.258 |
| rs7329174 | 13 | 41558110 | HGVM7615876 | 1.00E-08 | Imputed | 0.2009 | 0.4907 |
| rs72698115 | 1 | 154379369 | HGVM20849114 | 1.00E-08 | Imputed | 0.2341 | 1.387 |
| rs3825568 | 14 | 69260588 | HGVM2315320 | 1.00E-08 | Imputed | 0.2509 | 0.8245 |
| rs56380902 | 17 | 38066372 | HGVM13869513 | 1.00E-08 | Imputed | 0.3082 | 1.19 |
| rs1877030 | 17 | 37740161 | HGVM963109 | 1.00E-08 | Imputed | 0.3687 | 1.22 |
| rs10466829 | 12 | 9876091 | HGVM3194948 | 1.00E-08 | Imputed | 0.3717 | 1.168 |
| rs1432296 | 2 | 61068167 | HGVM1860625 | 1.00E-08 | Imputed | 0.4041 | 1.247 |
| rs59772922 | 15 | 79207466 | HGVM13402217 | 1.00E-08 | Imputed | 0.4398 | 0.8423 |
| rs11900673 | 2 | 62452661 | HGVM4545477 | 1.00E-08 | Imputed | 0.445 | 1.221 |
| rs2317230 | 1 | 157674997 | HGVM2073623 | 1.00E-08 | Imputed | 0.4614 | 0.8858 |
| rs4246905 | 9 | 117553249 | HGVM6332863 | 1.00E-08 | Imputed | 0.4849 | 0.8741 |
| rs4263037 | 18 | 60016233 | HGVM2466429 | 1.00E-08 | Genotyped | 0.4934 | 0.8785 |
| rs112846137 | 3 | 160312921 | HGVM34022784 | 1.00E-08 | Imputed | 0.5267 | 1.312 |
| rs10517039 | 4 | 42513936 | HGVM3240096 | 1.00E-08 | Imputed | 0.5516 | 0.7898 |
| rs10931468 | 2 | 191538562 | HGVM3638109 | 1.00E-08 | Genotyped | 0.6094 | 1.122 |
| rs7300146 | 12 | 129373073 | HGVM7589704 | 1.00E-08 | Genotyped | 0.6114 | 0.9122 |
| rs4788084 | 16 | 28539848 | HGVM2760328 | 1.00E-08 | Imputed | 0.6355 | 0.9235 |
| rs4917385 | 10 | 105003721 | HGVM9888285 | 1.00E-08 | Imputed | 0.6462 | 1.084 |
| rs1886700 | 16 | 68685905 | HGVM1936982 | 1.00E-08 | Genotyped | 0.7418 | 1.078 |
| rs10209110 | 2 | 100672692 | HGVM3077120 | 1.00E-08 | Imputed | 0.7755 | 1.051 |
| rs11102024 | 1 | 110431514 | HGVM3809741 | 1.00E-08 | Imputed | 0.8217 | 1.194 |
| rs2663054 | 10 | 50101977 | HGVM1294803 | 1.00E-08 | Imputed | 0.822 | 0.9617 |
| rs8023715 | 15 | 97607681 | HGVM8284898 | 1.00E-08 | Genotyped | 0.8301 | 1.069 |
| rs11719975 | 3 | 18785585 | HGVM4364475 | 1.00E-08 | Imputed | 0.8453 | 0.9631 |
| rs13101828 | 4 | 965720 | HGVM5747888 | 1.00E-08 | Imputed | 0.8596 | 0.9702 |
| rs874628 | 19 | 18304700 | HGVM1439510 | 1.00E-08 | Genotyped | 0.8777 | 1.03 |
| rs7195994 | 16 | 54060205 | HGVM7499922 | 1.00E-08 | Imputed | 0.8925 | 1.04 |
| rs78478398 | 12 | 80428530 | HGVM25399055 | 1.00E-08 | Imputed | 0.919 | 1.051 |
| rs2807264 | X | 135665778 | HGVM9756346 | 1.00E-08 | Imputed | 0.9253 | 0.9812 |
| rs7119038 | 11 | 118738281 | HGVM7425708 | 1.00E-08 | Imputed | 0.9296 | 0.979 |
| rs1444766 | 3 | 123925271 | HGVM160416 | 1.00E-08 | Imputed | 0.9545 | 1.012 |
| rs73081554 | 3 | 58302935 | HGVM22710376 | 1.00E-08 | Imputed | 0.9992 | 0.9997 |
| rs1465788 | 14 | 69263599 | HGVM450979 | 1.37E-08 | Imputed | 0.267 | 0.8033 |
| rs3024886 | 2 | 191900449 | HGVM1687109 | 1.84E-08 | Genotyped | 0.1711 | 1.325 |
| rs41272536 | 1 | 183440531 | HGVM19280436 | 1.88E-08 | Imputed | 0.3932 | 1.401 |
| rs6497238 | 15 | 28053778 | HGVM6869224 | 1.92E-08 | Genotyped | 0.903 | 0.9779 |
| rs2847297 | 18 | 12797694 | HGVM1434976 | 2.00E-08 | Genotyped | 8.14E-03 | 0.6161 |
| rs4761587 | 12 | 94551799 | HGVM2739867 | 2.00E-08 | Imputed | 0.02395 | 0.6006 |
| rs11059927 | 12 | 129294333 | HGVM3767295 | 2.00E-08 | Imputed | 0.03763 | 1.838 |
| rs3890745 | 1 | 2553624 | HGVM2339817 | 2.00E-08 | Genotyped | 0.04608 | 1.454 |
| rs8021741 | 14 | 76022400 | HGVM8282939 | 2.00E-08 | Genotyped | 0.05397 | 1.408 |
| rs394199 | 6 | 33553580 | HGVM253975 | 2.00E-08 | Genotyped | 0.06131 | 1.384 |
| rs1635852 | 7 | 28189411 | HGVM704112 | 2.00E-08 | Genotyped | 0.1018 | 0.7568 |
| rs5904818 | X | 147042774 | HGVM6584992 | 2.00E-08 | Genotyped | 0.1057 | 0.542 |
| rs960709 | 5 | 150461049 | HGVM234068 | 2.00E-08 | Genotyped | 0.1448 | 1.311 |
| rs2300603 | 14 | 76005557 | HGVM2068946 | 2.00E-08 | Genotyped | 0.1681 | 1.307 |
| rs1943199 | 18 | 73462854 | HGVM976628 | 2.00E-08 | Imputed | 0.1703 | 1.966 |
| rs56154925 | 19 | 55737798 | HGVM14353475 | 2.00E-08 | Imputed | 0.1791 | 1.344 |
| rs757123 | 12 | 120800823 | HGVM412271 | 2.00E-08 | Imputed | 0.1993 | 1.36 |
| rs2240335 | 1 | 17674537 | HGVM1238476 | 2.00E-08 | Genotyped | 0.2256 | 1.263 |
| rs975730 | 8 | 129316014 | HGVM1171730 | 2.00E-08 | Imputed | 0.2611 | 0.8193 |
| rs11746555 | 5 | 131727033 | HGVM4391083 | 2.00E-08 | Genotyped | 0.2804 | 1.199 |
| rs11788118 | 9 | 102337331 | HGVM4432697 | 2.00E-08 | Genotyped | 0.3008 | 0.8098 |
| rs9852465 | 3 | 58465183 | HGVM10324088 | 2.00E-08 | Imputed | 0.325 | 1.192 |
| rs11755393 | 6 | 34824636 | HGVM4399926 | 2.00E-08 | Genotyped | 0.3569 | 0.8531 |
| rs26595 | 5 | 115759490 | HGVM630806 | 2.00E-08 | Genotyped | 0.3701 | 0.8608 |
| rs11808092 | 1 | 93073228 | HGVM4452710 | 2.00E-08 | Genotyped | 0.4166 | 0.8521 |
| rs10516557 | 4 | 110395406 | HGVM3239615 | 2.00E-08 | Genotyped | 0.4225 | 0.7282 |
| rs2777899 | 17 | 57832391 | HGVM2176610 | 2.00E-08 | Imputed | 0.4252 | 0.8677 |
| rs11622435 | 14 | 81617996 | HGVM4266717 | 2.00E-08 | Imputed | 0.4368 | 1.411 |
| rs12529514 | 6 | 14096658 | HGVM5174125 | 2.00E-08 | Genotyped | 0.45 | 1.333 |
| rs2205986 | 1 | 210116112 | HGVM6145679 | 2.00E-08 | Imputed | 0.4717 | 0.8113 |
| rs150537045 | 8 | 114407100 | HGVM58897858 | 2.00E-08 | Imputed | 0.4821 | 0.5784 |
| rs12822507 | 12 | 12773521 | HGVM5467284 | 2.00E-08 | Imputed | 0.4896 | 1.129 |
| rs210131 | 6 | 33535466 | HGVM664254 | 2.00E-08 | Imputed | 0.5003 | 1.23 |
| rs11724582 | 4 | 123391464 | HGVM4369083 | 2.00E-08 | Imputed | 0.5007 | 1.145 |
| rs4264325 | 14 | 104979486 | HGVM2467032 | 2.00E-08 | Imputed | 0.5028 | 1.622 |
| rs1549922 | 5 | 158731548 | HGVM1878894 | 2.00E-08 | Imputed | 0.5433 | 0.9004 |
| rs968567 | 11 | 61595564 | HGVM391710 | 2.00E-08 | Imputed | 0.5472 | 1.146 |
| rs1876518 | 2 | 65608909 | HGVM1011219 | 2.00E-08 | Imputed | 0.5865 | 1.098 |
| rs3802604 | 10 | 8102272 | HGVM2299423 | 2.00E-08 | Genotyped | 0.5873 | 0.9108 |
| rs2284033 | 22 | 37534034 | HGVM1482735 | 2.00E-08 | Genotyped | 0.5908 | 0.9098 |
| rs4690229 | 4 | 970724 | HGVM2687461 | 2.00E-08 | Imputed | 0.5994 | 0.9153 |
| rs883220 | 1 | 38616871 | HGVM1225081 | 2.00E-08 | Genotyped | 0.6182 | 1.108 |
| rs66534072 | 22 | 21936152 | HGVM22474317 | 2.00E-08 | Imputed | 0.6407 | 1.094 |
| rs9976767 | 21 | 43836390 | HGVM8947777 | 2.00E-08 | Genotyped | 0.709 | 0.9396 |
| rs4285028 | 3 | 121660664 | HGVM2477013 | 2.00E-08 | Imputed | 0.7107 | 1.071 |
| rs2745106 | 16 | 1541461 | HGVM1409272 | 2.00E-08 | Genotyped | 0.7356 | 0.9138 |
| rs2618476 | 8 | 11352541 | HGVM1193093 | 2.00E-08 | Genotyped | 0.7888 | 0.9486 |
| rs1170426 | 16 | 68603798 | HGVM475566 | 2.00E-08 | Imputed | 0.8026 | 0.9497 |
| rs806321 | 13 | 50841323 | HGVM431825 | 2.00E-08 | Genotyped | 0.8323 | 0.9633 |
| rs180977001 | 3 | 58318464 | HGVM51425816 | 2.00E-08 | Imputed | 0.8514 | 0.9396 |
| rs6062314 | 20 | 62409713 | HGVM6711142 | 2.00E-08 | Genotyped | 0.8716 | 1.049 |
| rs7200786 | 16 | 11177801 | HGVM7504592 | 2.00E-08 | Imputed | 0.8854 | 0.9765 |
| rs17714860 | 17 | 68272354 | HGVM13785051 | 2.00E-08 | Imputed | 0.9663 | 0.9926 |
| rs7127214 | 11 | 36343693 | HGVM7433452 | 2.00E-08 | Imputed | 0.967 | 1.008 |
| rs2269060 | 9 | 123683569 | HGVM1210640 | 2.00E-08 | Imputed | 0.9676 | 1.007 |
| rs170934 | 3 | 28079085 | HGVM585058 | 2.00E-08 | Genotyped | 0.9869 | 0.9973 |
| rs1167796 | 7 | 75173180 | HGVM304790 | 2.00E-08 | Genotyped | 0.9892 | 1.002 |
| rs116199914 | 3 | 25638355 | HGVM34285966 | 2.00E-08 | Imputed | 0.9976 | 0.998 |
| rs9585056 | 13 | 100081766 | HGVM8683231 | 2.05E-08 | Genotyped | 0.07035 | 0.7105 |
| rs4238595 | 16 | 20343091 | HGVM2453889 | 2.14E-08 | Genotyped | 0.9825 | 1.004 |
| rs3117222 | 6 | 33060949 | HGVM1596411 | 2.57E-08 | Genotyped | 0.4102 | 0.85 |
| rs11203368 | 1 | 17666508 | HGVM3911530 | 2.61E-08 | Imputed | 0.9994 | 0.9999 |
| rs2836425 | 21 | 39838825 | HGVM1476374 | 2.84E-08 | Genotyped | 0.2254 | 1.393 |
| rs6565228 | 16 | 31329280 | HGVM6925829 | 2.88E-08 | Imputed | 0.7735 | 1.073 |
| rs12465689 | 2 | 191912540 | HGVM5110207 | 2.89E-08 | Imputed | 0.1588 | 1.339 |
| rs1891621 | 10 | 31390127 | HGVM835027 | 2.94E-08 | Imputed | 0.1833 | 0.7892 |
| rs16833214 | 2 | 191913642 | HGVM14937417 | 2.99E-08 | Imputed | 0.1711 | 1.325 |
| rs13306575 | 1 | 183532437 | HGVM5954387 | 3.00E-08 | Genotyped | 0.01796 | 4.858 |
| rs9880772 | 3 | 27777779 | HGVM8863400 | 3.00E-08 | Imputed | 0.07266 | 1.382 |
| rs6946509 | 7 | 22809490 | HGVM7265485 | 3.00E-08 | Imputed | 0.08527 | 1.385 |
| rs73194058 | 21 | 34764288 | HGVM22456288 | 3.00E-08 | Imputed | 0.08763 | 1.601 |
| rs4739134 | 8 | 79556148 | HGVM2722925 | 3.00E-08 | Genotyped | 0.1123 | 0.7246 |
| rs62324212 | 4 | 123560939 | HGVM16899980 | 3.00E-08 | Imputed | 0.1432 | 0.7719 |
| rs2765974 | 10 | 11288418 | HGVM1297453 | 3.00E-08 | Genotyped | 0.164 | 1.265 |
| rs2834512 | 21 | 35911599 | HGVM1474648 | 3.00E-08 | Genotyped | 0.1976 | 1.459 |
| rs1983890 | 10 | 6178614 | HGVM1967829 | 3.00E-08 | Genotyped | 0.2004 | 1.258 |
| rs6579837 | 5 | 150434894 | HGVM6938845 | 3.00E-08 | Imputed | 0.2142 | 1.426 |
| rs7197475 | 16 | 30642867 | HGVM7501363 | 3.00E-08 | Imputed | 0.2162 | 0.8171 |
| rs67927699 | 2 | 61187415 | HGVM22100568 | 3.00E-08 | Imputed | 0.3174 | 0.8279 |
| rs7069750 | 10 | 90762376 | HGVM7378555 | 3.00E-08 | Imputed | 0.3379 | 1.174 |
| rs11218708 | 11 | 122472063 | HGVM3926913 | 3.00E-08 | Genotyped | 0.3395 | 1.26 |
| rs12988804 | 2 | 170117811 | HGVM5633949 | 3.00E-08 | Genotyped | 0.4708 | 1.143 |
| rs6696533 | 1 | 198733567 | HGVM7027692 | 3.00E-08 | Imputed | 0.505 | 0.8905 |
| rs221781 | 7 | 100295908 | HGVM289847 | 3.00E-08 | Imputed | 0.5297 | 0.8367 |
| rs2664035 | 4 | 48220839 | HGVM1080312 | 3.00E-08 | Imputed | 0.5482 | 0.8988 |
| rs4320356 | 6 | 26423560 | HGVM2493911 | 3.00E-08 | Genotyped | 0.5854 | 1.101 |
| rs1292034 | 17 | 57989860 | HGVM959954 | 3.00E-08 | Genotyped | 0.6077 | 0.911 |
| rs12531540 | 7 | 28162674 | HGVM5176151 | 3.00E-08 | Imputed | 0.6306 | 1.086 |
| rs8061370 | 16 | 11458471 | HGVM8322287 | 3.00E-08 | Imputed | 0.6463 | 1.107 |
| rs2295463 | 14 | 35736273 | HGVM2067337 | 3.00E-08 | Imputed | 0.697 | 0.793 |
| rs11145763 | 9 | 139263596 | HGVM3853729 | 3.00E-08 | Imputed | 0.7057 | 0.9373 |
| rs1107345 | 10 | 6087295 | HGVM9493172 | 3.00E-08 | Genotyped | 0.7552 | 1.066 |
| rs11673987 | 2 | 65597671 | HGVM4318374 | 3.00E-08 | Imputed | 0.7847 | 0.9549 |
| rs7172677 | 15 | 75424593 | HGVM7477328 | 3.00E-08 | Genotyped | 0.8624 | 0.9676 |
| rs62447205 | 7 | 50465830 | HGVM20627164 | 3.00E-08 | Imputed | 0.8772 | 0.9719 |
| rs2150702 | 9 | 5893861 | HGVM1208307 | 3.00E-08 | Imputed | 0.8928 | 1.022 |
| rs2422345 | 1 | 173337747 | HGVM1616478 | 3.00E-08 | Imputed | 0.9366 | 0.9861 |
| rs911263 | 14 | 68753593 | HGVM445207 | 3.00E-08 | Imputed | 0.9395 | 0.9855 |
| rs595158 | 11 | 60909581 | HGVM379618 | 3.00E-08 | Genotyped | 0.9565 | 1.01 |
| rs7804356 | 7 | 26891665 | HGVM8069862 | 3.27E-08 | Imputed | 0.2362 | 1.267 |
| rs4849135 | 2 | 111615079 | HGVM2805621 | 4.00E-08 | Imputed | 5.28E-04 | 2.043 |
| rs10202630 | 2 | 191262925 | HGVM3071546 | 4.00E-08 | Imputed | 0.03564 | 1.453 |
| rs9785133 | 8 | 20358618 | HGVM8789007 | 4.00E-08 | Imputed | 0.03874 | 0.5939 |
| rs11066301 | 12 | 112871372 | HGVM3773720 | 4.00E-08 | Genotyped | 0.06204 | 0.7137 |
| rs12048904 | 1 | 101331536 | HGVM4693921 | 4.00E-08 | Imputed | 0.07447 | 1.369 |
| rs1780813 | 1 | 246444082 | HGVM803111 | 4.00E-08 | Genotyped | 0.1379 | 1.817 |
| rs4972593 | 2 | 174462854 | HGVM2891574 | 4.00E-08 | Imputed | 0.1961 | 1.359 |
| rs26232 | 5 | 102596720 | HGVM194323 | 4.00E-08 | Imputed | 0.2125 | 0.7921 |
| rs114038709 | 17 | 43456728 | HGVM32595800 | 4.00E-08 | Imputed | 0.2262 | 1.249 |
| rs12722489 | 10 | 6102012 | HGVM5367070 | 4.00E-08 | Genotyped | 0.2266 | 0.7465 |
| rs6479800 | 10 | 64036881 | HGVM6853277 | 4.00E-08 | Imputed | 0.2501 | 0.772 |
| rs17604670 | 4 | 113207277 | HGVM16309408 | 4.00E-08 | Genotyped | 0.3255 | 0.762 |
| rs2726518 | 4 | 106173199 | HGVM2166277 | 4.00E-08 | Imputed | 0.3328 | 0.8501 |
| rs72830848 | 17 | 55398579 | HGVM21867669 | 4.00E-08 | Imputed | 0.3359 | 0.6631 |
| rs16975792 | 17 | 68433725 | HGVM13781966 | 4.00E-08 | Imputed | 0.3407 | 1.19 |
| rs78368496 | 11 | 19984329 | HGVM25115675 | 4.00E-08 | Imputed | 0.4118 | 0.6588 |
| rs2688608 | 10 | 75658349 | HGVM1295795 | 4.00E-08 | Imputed | 0.4209 | 1.155 |
| rs10203477 | 2 | 61104985 | HGVM3072284 | 4.00E-08 | Imputed | 0.4938 | 0.8841 |
| rs2085277 | 13 | 32167717 | HGVM1355121 | 4.00E-08 | Imputed | 0.494 | 1.592 |
| rs1054609 | 17 | 38033277 | HGVM959285 | 4.00E-08 | Imputed | 0.5113 | 1.122 |
| rs12980063 | 19 | 50196992 | HGVM5625189 | 4.00E-08 | Imputed | 0.549 | 1.111 |
| rs10245867 | 7 | 28142186 | HGVM3106540 | 4.00E-08 | Imputed | 0.5749 | 0.9002 |
| rs7537605 | 1 | 108343087 | HGVM7804780 | 4.00E-08 | Genotyped | 0.5763 | 0.9086 |
| rs881375 | 9 | 123652898 | HGVM733817 | 4.00E-08 | Genotyped | 0.5787 | 0.9057 |
| rs12901682 | 15 | 78833223 | HGVM5546633 | 4.00E-08 | Genotyped | 0.6369 | 1.516 |
| rs6691977 | 1 | 200814959 | HGVM7023380 | 4.00E-08 | Imputed | 0.6437 | 1.098 |
| rs4305317 | 2 | 42080624 | HGVM2486661 | 4.00E-08 | Genotyped | 0.645 | 1.083 |
| rs17785991 | 20 | 48438761 | HGVM14490756 | 4.00E-08 | Imputed | 0.6834 | 0.9303 |
| rs13093110 | 3 | 188125120 | HGVM5739098 | 4.00E-08 | Imputed | 0.7311 | 1.062 |
| rs11571302 | 2 | 204742934 | HGVM4216125 | 4.00E-08 | Imputed | 0.7764 | 1.05 |
| rs6618677 | X | 90812718 | HGVM6967912 | 4.00E-08 | Imputed | 0.8394 | 0.9543 |
| rs11954020 | 5 | 35883251 | HGVM4598954 | 4.00E-08 | Imputed | 0.8586 | 0.968 |
| rs10999147 | 10 | 71880858 | HGVM3706166 | 4.00E-08 | Imputed | 0.9463 | 1.019 |
| rs2953898 | 8 | 56980803 | HGVM1561002 | 4.00E-08 | Imputed | 0.9648 | 0.9908 |
| rs11150615 | 16 | 31369803 | HGVM3858609 | 4.28E-08 | Imputed | 0.4981 | 1.164 |
| rs9385400 | 6 | 126764190 | HGVM8518486 | 4.55E-08 | Imputed | 0.446 | 1.142 |
| rs34286592 | 16 | 29820480 | HGVM13521523 | 4.58E-08 | Genotyped | 0.7153 | 1.086 |
| rs2793108 | 10 | 31379105 | HGVM1298879 | 4.80E-08 | Genotyped | 0.6549 | 0.9253 |
| rs55849330 | 5 | 100184647 | HGVM17107652 | 5.00E-08 | Imputed | 0.02332 | 1.559 |
| rs13315591 | 3 | 58556841 | HGVM5963421 | 5.00E-08 | Imputed | 0.06472 | 1.947 |
| rs11984075 | 7 | 37436854 | HGVM4629047 | 5.00E-08 | Imputed | 0.2086 | 0.6945 |
| rs2582532 | 14 | 105392837 | HGVM1373802 | 5.00E-08 | Imputed | 0.2578 | 1.65 |
| rs137956 | 22 | 40293463 | HGVM1480180 | 5.00E-08 | Imputed | 0.3529 | 0.8496 |
| rs240753 | 20 | 17861814 | HGVM525774 | 5.00E-08 | Imputed | 0.4068 | 0.8686 |
| rs11121380 | 1 | 9408959 | HGVM3829239 | 5.00E-08 | Imputed | 0.4428 | 0.7788 |
| rs114558062 | 3 | 187641130 | HGVM34142288 | 5.00E-08 | Imputed | 0.4513 | 0.5571 |
| rs602662 | 19 | 49206985 | HGVM519402 | 5.00E-08 | Genotyped | 0.4929 | 0.8849 |
| rs9532434 | 13 | 40355913 | HGVM8635245 | 5.00E-08 | Imputed | 0.5049 | 0.8893 |
| rs1032129 | 8 | 119951900 | HGVM1495966 | 5.00E-08 | Imputed | 0.6376 | 1.091 |
| rs402072 | 19 | 47219122 | HGVM517320 | 5.00E-08 | Imputed | 0.7404 | 0.9204 |
| rs12413578 | 10 | 9049253 | HGVM5057990 | 5.00E-08 | Imputed | 0.7998 | 0.9226 |
| rs2836882 | 21 | 40466570 | HGVM1476877 | 5.00E-08 | Genotyped | 0.8425 | 0.9632 |
| rs2275806 | 10 | 8095340 | HGVM1282419 | 5.00E-08 | Imputed | 0.8511 | 1.032 |
| rs12565755 | 1 | 61041875 | HGVM5210430 | 5.00E-08 | Genotyped | 0.8662 | 0.9606 |
| rs113010081 | 3 | 46457412 | HGVM34032649 | 5.00E-08 | Imputed | 0.8881 | 0.9533 |
| rs9860428 | 3 | 112570919 | HGVM10328473 | 5.00E-08 | Imputed | 0.8895 | 0.9756 |
| rs2222631 | 3 | 119272391 | HGVM1038077 | 5.00E-08 | Imputed | 0.9697 | 0.9933 |
