# Supplementary material for: A genome-wide screen for variants influencing certolizumab pegol response in a moderate to severe rheumatoid arthritis population
Source: PLoS One. 2022 Apr 12;17(4):e0261165. doi: 10.1371/journal.pone.0261165 (PMC9004786; doi:10.1371/journal.pone.0261165)
Supplement: S3 Table — (DOCX) [file pone.0261165.s006.docx]

| SNP | Chromosome | Position | identifier | Reported P-value | Dataset | P-value (ACR20 at Wk 6) | OR |
| --- | --- | --- | --- | --- | --- | --- | --- |
| rs6910071 | 6 | 32282854 | HGVM7231436 | 1.00E-299 | Genotyped | 0.9514 | 0.9888 |
| rs9268853 | 6 | 32429643 | HGVM8420027 | 5.00E-109 | Genotyped | 0.09684 | 0.7442 |
| rs13192471 | 6 | 32671103 | HGVM5839446 | 2.00E-58 | Genotyped | 0.3853 | 0.832 |
| rs9272219 | 6 | 32602269 | HGVM10528836 | 1.00E-45 | Genotyped | 0.6576 | 1.093 |
| rs6679677 | 1 | 114303808 | HGVM7011783 | 6.00E-42 | Imputed | 0.04668 | 0.6068 |
| rs10484554 | 6 | 31274555 | HGVM3209069 | 2.00E-39 | Genotyped | 0.3036 | 0.7537 |
| rs9296015 | 6 | 32218989 | HGVM8439581 | 2.00E-38 | Genotyped | 0.4692 | 0.849 |
| rs2227139 | 6 | 32413459 | HGVM1527334 | 6.73E-29 | Genotyped | 0.9414 | 0.986 |
| rs2395029 | 6 | 31431780 | HGVM1126716 | 2.00E-26 | Imputed | 0.05856 | 0.3114 |
| rs33980500 | 6 | 111913262 | HGVM17514152 | 1.00E-20 | Imputed | 0.3904 | 1.318 |
| rs9296021 | 6 | 32297690 | HGVM8439587 | 1.90E-19 | Genotyped | 0.4547 | 0.8796 |
| rs653178 | 12 | 112007756 | HGVM867561 | 3.00E-19 | Imputed | 7.98E-03 | 0.6191 |
| rs3093024 | 6 | 167532793 | HGVM1589373 | 8.00E-19 | Genotyped | 0.9513 | 1.01 |
| rs12188300 | 5 | 158829527 | HGVM4833321 | 7.00E-17 | Imputed | 0.7435 | 1.127 |
| rs874040 | 4 | 26108197 | HGVM179972 | 1.00E-16 | Imputed | 0.7409 | 0.9379 |
| rs1610677 | 6 | 29789171 | HGVM681100 | 4.00E-15 | Genotyped | 0.3214 | 0.8521 |
| rs11676922 | 2 | 100806940 | HGVM4321311 | 1.00E-14 | Imputed | 0.6998 | 1.066 |
| rs3862488 | 19 | 23089316 | HGVM6279258 | 1.00E-14 | Genotyped | 0.9426 | 0.976 |
| rs10210302 | 2 | 234158839 | HGVM3078144 | 5.26E-14 | Imputed | 0.4638 | 1.13 |
| rs6920220 | 6 | 138006504 | HGVM7240882 | 9.00E-13 | Genotyped | 0.6082 | 1.106 |
| rs10892279 | 11 | 118611781 | HGVM3598706 | 1.00E-12 | Imputed | 0.6368 | 1.114 |
| rs2066843 | 16 | 50745199 | HGVM954548 | 1.79E-12 | Imputed | 0.3894 | 0.838 |
| rs17234657 | 5 | 40401509 | HGVM16945469 | 1.99E-12 | Genotyped | 0.1057 | 0.6488 |
| rs5029939 | 6 | 138195723 | HGVM6426387 | 3.00E-12 | Genotyped | 0.01976 | 0.3267 |
| rs1893217 | 18 | 12809340 | HGVM976103 | 5.00E-12 | Genotyped | 0.1244 | 0.6724 |
| rs11805303 | 1 | 67675516 | HGVM4449920 | 5.85E-12 | Genotyped | 0.1033 | 1.392 |
| rs10499194 | 6 | 138002637 | HGVM3222613 | 6.00E-12 | Imputed | 0.5767 | 0.9021 |
| rs6859219 | 5 | 55438580 | HGVM7183242 | 1.00E-11 | Imputed | 0.3032 | 1.254 |
| rs706778 | 10 | 6098949 | HGVM817038 | 1.00E-11 | Imputed | 0.9548 | 1.01 |
| rs864537 | 1 | 167411384 | HGVM773882 | 2.00E-11 | Genotyped | 0.03209 | 0.6874 |
| rs3093023 | 6 | 167534290 | HGVM1589372 | 2.00E-11 | Imputed | 0.9153 | 0.9824 |
| rs17221417 | 16 | 50739582 | HGVM13489291 | 3.98E-11 | Imputed | 0.3916 | 0.8471 |
| rs10488631 | 7 | 128594183 | HGVM3212077 | 4.00E-11 | Genotyped | 0.1278 | 0.6706 |
| rs1953126 | 9 | 123640500 | HGVM1958077 | 4.00E-11 | Genotyped | 0.4313 | 0.8722 |
| rs3821236 | 2 | 191902758 | HGVM2312422 | 8.00E-11 | Genotyped | 0.1711 | 1.325 |
| rs9277378 | 6 | 33050279 | HGVM8423357 | 2.00E-10 | Genotyped | 0.4055 | 0.8492 |
| rs9277463 | 6 | 33053307 | HGVM8423440 | 2.93E-10 | Genotyped | 0.3461 | 0.8309 |
| rs2298428 | 22 | 21982892 | HGVM1482715 | 3.00E-10 | Imputed | 0.5157 | 1.133 |
| rs805297 | 6 | 31622606 | HGVM269803 | 3.00E-10 | Genotyped | 0.9393 | 0.9873 |
| rs7574865 | 2 | 191964633 | HGVM7841836 | 4.00E-10 | Genotyped | 0.1866 | 1.285 |
| rs951005 | 9 | 34743681 | HGVM733202 | 4.00E-10 | Imputed | 0.9713 | 0.9924 |
| rs934734 | 2 | 65595586 | HGVM1695357 | 5.00E-10 | Imputed | 0.7446 | 1.056 |
| rs2073045 | 6 | 32339548 | HGVM1119250 | 5.00E-10 | Genotyped | 0.9486 | 1.011 |
| rs10491033 | 10 | 86186241 | HGVM3214455 | 7.00E-10 | Genotyped | 0.4828 | 1.427 |
| rs2395173 | 6 | 32404859 | HGVM1533371 | 7.00E-10 | Genotyped | 0.86 | 0.9672 |
| rs3130190 | 6 | 33061690 | HGVM1598947 | 8.00E-10 | Genotyped | 0.4102 | 0.85 |
| rs4810485 | 20 | 44747947 | HGVM2777373 | 3.00E-09 | Genotyped | 0.4431 | 1.183 |
| rs2075876 | 21 | 45709153 | HGVM1456700 | 4.00E-09 | Imputed | 0.6796 | 0.8841 |
| rs10517039 | 4 | 42513936 | HGVM3240096 | 1.00E-08 | Imputed | 0.5516 | 0.7898 |
| rs11203203 | 21 | 43836186 | HGVM3911364 | 1.00E-08 | Genotyped | 0.5524 | 1.11 |
| rs3087243 | 2 | 204738919 | HGVM1690397 | 1.00E-08 | Genotyped | 0.5859 | 1.097 |
| rs7579944 | 2 | 30445026 | HGVM7846936 | 1.00E-08 | Genotyped | 0.7684 | 0.9484 |
| rs6497238 | 15 | 28053778 | HGVM6869224 | 1.92E-08 | Genotyped | 0.903 | 0.9779 |
| rs2240335 | 1 | 17674537 | HGVM1238476 | 2.00E-08 | Genotyped | 0.2256 | 1.263 |
| rs975730 | 8 | 129316014 | HGVM1171730 | 2.00E-08 | Imputed | 0.2611 | 0.8193 |
| rs10516557 | 4 | 110395406 | HGVM3239615 | 2.00E-08 | Genotyped | 0.4225 | 0.7282 |
| rs1876518 | 2 | 65608909 | HGVM1011219 | 2.00E-08 | Imputed | 0.5865 | 1.098 |
| rs2618476 | 8 | 11352541 | HGVM1193093 | 2.00E-08 | Genotyped | 0.7888 | 0.9486 |
| rs3117222 | 6 | 33060949 | HGVM1596411 | 2.57E-08 | Genotyped | 0.4102 | 0.85 |
| rs2812378 | 9 | 34710260 | HGVM1222007 | 3.00E-08 | Genotyped | 0.965 | 0.9923 |
| rs9858542 | 3 | 49701983 | HGVM8844489 | 3.58E-08 | Genotyped | 0.9484 | 1.013 |
| rs26232 | 5 | 102596720 | HGVM194323 | 4.00E-08 | Imputed | 0.2125 | 0.7921 |
| rs13315591 | 3 | 58556841 | HGVM5963421 | 5.00E-08 | Imputed | 0.06472 | 1.947 |
| rs11984075 | 7 | 37436854 | HGVM4629047 | 5.00E-08 | Imputed | 0.2086 | 0.6945 |
