# Supplementary material for: A genome-wide screen for variants influencing certolizumab pegol response in a moderate to severe rheumatoid arthritis population
Source: PLoS One. 2022 Apr 12;17(4):e0261165. doi: 10.1371/journal.pone.0261165 (PMC9004786; doi:10.1371/journal.pone.0261165)
Supplement: S4 Table — (DOCX) [file pone.0261165.s007.docx]

| SNP | Chromosome | Position | Gene | Dataset | P-value  (ACR20 at Wk 6) | OR |
| --- | --- | --- | --- | --- | --- | --- |
| rs1485766 | 4 | 177610884 | *VEGFC* | Genotyped | 2.24E-04 | 0.5185 |
| rs2236304 | 14 | 23312863 | *MMP14* | Imputed | 5.45E-04 | 1.981 |
| rs2236303 | 14 | 23312718 | *MMP14* | Imputed | 1.04E-03 | 1.878 |
| rs475342 | 6 | 149638396 | *TAB2* | Imputed | 2.26E-03 | 1.842 |
| rs8006914 | 14 | 23310485 | *MMP14* | Genotyped | 2.59E-03 | 1.757 |
| rs6932778 | 6 | 149649429 | *TAB2* | Imputed | 3.19E-03 | 1.916 |
| rs2236302 | 14 | 23312554 | *MMP14* | Imputed | 3.69E-03 | 0.3941 |
| rs5029949 | 6 | 138197506 | *TNFAIP3* | Imputed | 3.83E-03 | 0.2361 |
| rs4851005 | 2 | 103011552 | *IL18R1* | Genotyped | 4.28E-03 | 0.5747 |
| rs6059876 | 20 | 33086982 | *ITCH* | Imputed | 4.44E-03 | 0.5953 |
| rs6579167 | 20 | 33032915 | *ITCH* | Imputed | 4.80E-03 | 0.5118 |
| rs5029924 | 6 | 138187498 | *TNFAIP3* | Imputed | 4.89E-03 | 0.2626 |
| rs10225620 | 7 | 28492664 | *CREB5* | Imputed | 4.94E-03 | 0.5605 |
| rs1049728 | 11 | 65421117 | *RELA* | Genotyped | 5.40E-03 | 0.3167 |
| rs112860535 | 6 | 149569389 | *TAB2* | Imputed | 5.55E-03 | 3.758 |
| rs111978421 | 6 | 149569504 | *TAB2* | Imputed | 5.55E-03 | 3.758 |
| rs61252638 | 6 | 149569877 | *TAB2* | Imputed | 5.55E-03 | 3.758 |
| rs7674004 | 4 | 103540313 | *NFKB1* | Imputed | 5.61E-03 | 0.6042 |
| rs7674640 | 4 | 103540780 | *NFKB1* | Imputed | 5.61E-03 | 0.6042 |
| rs12155324 | 7 | 137665638 | *CREB3L2* | Imputed | 6.19E-03 | 0.5809 |
| rs522205 | 1 | 12249232 | *TNFRSF1B* | Imputed | 6.52E-03 | 0.5359 |
| rs1420095 | 2 | 103012902 | *IL18R1* | Imputed | 6.60E-03 | 0.3911 |
| rs6921428 | 6 | 149602683 | *TAB2* | Imputed | 6.70E-03 | 1.661 |
| rs143220164 | 1 | 211547220 | *TRAF5* | Imputed | 6.91E-03 | 0.3957 |
| rs6911411 | 6 | 149688090 | *TAB2* | Genotyped | 6.92E-03 | 1.786 |
| rs11465613 | 2 | 102991056 | *IL18R1* | Imputed | 6.97E-03 | 0.3936 |
| rs11465620 | 2 | 102991229 | *IL18R1* | Imputed | 6.97E-03 | 0.3936 |
| rs56030066 | 2 | 103003034 | *IL18R1* | Imputed | 6.97E-03 | 0.3936 |
| rs11167234 | 20 | 32983619 | *ITCH* | Imputed | 7.22E-03 | 1.634 |
| rs5029937 | 6 | 138195151 | *TNFAIP3* | Imputed | 7.50E-03 | 0.2801 |
| rs73090540 | 7 | 28448594 | *CREB5* | Imputed | 7.54E-03 | 0.5307 |
| rs76194790 | 7 | 28493761 | *CREB5* | Imputed | 7.77E-03 | 0.5817 |
| rs2269212 | 14 | 23310150 | *MMP14* | Imputed | 7.82E-03 | 1.66 |
| rs10927029 | 1 | 243662773 | *SDCCAG8* | Genotyped | 7.83E-03 | 1.828 |
| rs13232030 | 7 | 137663477 | *CREB3L2* | Genotyped | 7.89E-03 | 0.5932 |
| rs473451 | 6 | 149638198 | *TAB2* | Imputed | 7.91E-03 | 1.656 |
| rs831124 | 5 | 67574750 | *PIK3R1* | Imputed | 8.10E-03 | 1.621 |
| rs17156694 | 7 | 28484955 | *CREB5* | Genotyped | 8.18E-03 | 2.62 |
| rs525283 | 1 | 12241469 | *TNFRSF1B* | Imputed | 8.25E-03 | 7.454 |
| rs62079138 | 17 | 76359676 | *SOCS3* | Imputed | 8.31E-03 | 0.5283 |
| rs6058052 | 20 | 33047084 | *ITCH* | Imputed | 8.31E-03 | 0.6255 |
| rs73601901 | 6 | 149569229 | *TAB2* | Imputed | 8.41E-03 | 3.534 |
| rs73603503 | 6 | 149569316 | *TAB2* | Imputed | 8.41E-03 | 3.534 |
| rs6059880 | 20 | 33088669 | *ITCH* | Imputed | 8.44E-03 | 0.6273 |
| rs666078 | 1 | 12251318 | *TNFRSF1B* | Genotyped | 8.72E-03 | 0.5493 |
| rs2230926 | 6 | 138196066 | *TNFAIP3* | Genotyped | 8.87E-03 | 0.2905 |
| rs73601887 | 6 | 149560026 | *TAB2* | Imputed | 8.91E-03 | 3.497 |
| rs73601898 | 6 | 149568743 | *TAB2* | Imputed | 8.91E-03 | 3.497 |
| rs17084608 | 6 | 149569755 | *TAB2* | Imputed | 8.91E-03 | 3.497 |
| rs80156528 | 6 | 149572733 | *TAB2* | Imputed | 8.91E-03 | 3.497 |
| rs6088485 | 20 | 32985992 | *ITCH* | Imputed | 8.94E-03 | 1.609 |
| rs76358155 | 14 | 103333255 | *TRAF3* | Imputed | 9.31E-03 | 0.4863 |
| rs636964 | 1 | 12253351 | *TNFRSF1B* | Genotyped | 9.34E-03 | 0.5365 |
| rs73075810 | 7 | 28484651 | *CREB5* | Imputed | 9.96E-03 | 2.558 |
| rs274004 | 7 | 137642759 | *CREB3L2* | Imputed | 1.00E-02 | 1.601 |
| rs972634 | 5 | 67551471 | *PIK3R1* | Imputed | 0.01004 | 1.834 |
| rs519064 | 1 | 12242096 | *TNFRSF1B* | Imputed | 0.01018 | 7.092 |
| rs114642381 | 1 | 12242205 | *TNFRSF1B* | Imputed | 0.01018 | 7.092 |
| rs142443184 | 1 | 12245401 | *TNFRSF1B* | Imputed | 0.01018 | 7.092 |
| rs2064705 | 20 | 33031276 | *ITCH* | Imputed | 0.0102 | 1.602 |
| rs550132 | 1 | 12241050 | *TNFRSF1B* | Imputed | 0.01034 | 5.188 |
| rs7744549 | 6 | 149593817 | *TAB2* | Imputed | 0.01047 | 3.176 |
| rs6059858 | 20 | 33062425 | *ITCH* | Imputed | 0.01052 | 1.599 |
| rs5745994 | 1 | 12247128 | *TNFRSF1B* | Imputed | 0.01055 | 7.025 |
| rs2301258 | 1 | 12247940 | *TNFRSF1B* | Imputed | 0.01055 | 7.025 |
| rs5746007 | 1 | 12249305 | *TNFRSF1B* | Imputed | 0.01055 | 7.025 |
| rs5746011 | 1 | 12250004 | *TNFRSF1B* | Imputed | 0.01055 | 7.025 |
| rs5746012 | 1 | 12250188 | *TNFRSF1B* | Imputed | 0.01055 | 7.025 |
| rs5746017 | 1 | 12251341 | *TNFRSF1B* | Imputed | 0.01055 | 7.025 |
| rs4142007 | 20 | 32990050 | *ITCH* | Imputed | 0.01123 | 0.6389 |
| rs6142159 | 20 | 32993035 | *ITCH* | Imputed | 0.01123 | 0.6389 |
| rs6059824 | 20 | 33014543 | *ITCH* | Imputed | 0.01123 | 0.6389 |
| kgp5513607 | 20 | 33018008 | *ITCH* | kgp5513607 | 0.01123 | 0.6389 |
| rs6059844 | 20 | 33036482 | *ITCH* | Imputed | 0.01123 | 0.6389 |
| rs6088502 | 20 | 33037644 | *ITCH* | Imputed | 0.01123 | 0.6389 |
| rs6059856 | 20 | 33057954 | *ITCH* | Imputed | 0.01123 | 0.6389 |
| rs6087588 | 20 | 33066369 | *ITCH* | Imputed | 0.01123 | 0.6389 |
| rs6059867 | 20 | 33078103 | *ITCH* | Imputed | 0.01123 | 0.6389 |
| rs6059875 | 20 | 33086090 | *ITCH* | Imputed | 0.01123 | 0.6389 |
| rs6058064 | 20 | 33086622 | *ITCH* | Imputed | 0.01123 | 0.6389 |
| rs6088512 | 20 | 33095891 | *ITCH* | Genotyped | 0.01123 | 0.6389 |
| rs7271970 | 20 | 32987666 | *ITCH* | Imputed | 0.01141 | 0.6397 |
| rs62212172 | 20 | 32987705 | *ITCH* | Imputed | 0.01141 | 0.6397 |
| rs12480410 | 20 | 32988678 | *ITCH* | Imputed | 0.01141 | 0.6397 |
| rs6120663 | 20 | 33081906 | *ITCH* | Imputed | 0.01147 | 0.6239 |
| rs6059863 | 20 | 33069600 | *ITCH* | Imputed | 0.01147 | 0.6393 |
| rs2275416 | 1 | 12254201 | *TNFRSF1B* | Imputed | 0.01159 | 0.5628 |
| rs9378764 | 6 | 3086856 | *RIPK1* | Genotyped | 0.01165 | 2.151 |
| rs6059843 | 20 | 33036128 | *ITCH* | Imputed | 0.01166 | 0.6397 |
| rs5745975 | 1 | 12239151 | *TNFRSF1B* | Imputed | 0.01184 | 5.001 |
| rs632756 | 1 | 12239520 | *TNFRSF1B* | Imputed | 0.01184 | 5.001 |
| rs16897561 | 5 | 67560716 | *PIK3R1* | Genotyped | 0.01187 | 1.802 |
| rs56352616 | 5 | 67560996 | *PIK3R1* | Imputed | 0.01187 | 1.802 |
| rs1819986 | 5 | 67567076 | *PIK3R1* | Imputed | 0.01187 | 1.802 |
| rs3730082 | 5 | 67570138 | *PIK3R1* | Genotyped | 0.01187 | 1.802 |
| rs7716675 | 5 | 67571771 | *PIK3R1* | Genotyped | 0.01187 | 1.802 |
| rs7735204 | 5 | 67572167 | *PIK3R1* | Imputed | 0.01187 | 1.802 |
| rs6860081 | 5 | 67572371 | *PIK3R1* | Imputed | 0.01187 | 1.802 |
| rs545656 | 6 | 149637979 | *TAB2* | Imputed | 0.01191 | 1.608 |
| rs74153939 | 1 | 243862813 | *AKT3* | Imputed | 0.01203 | 0.2189 |
| rs7533826 | 1 | 243867327 | *AKT3* | Imputed | 0.01203 | 0.2189 |
| rs57774269 | 1 | 243872304 | *AKT3* | Imputed | 0.01203 | 0.2189 |
| rs74151205 | 1 | 243885328 | *AKT3* | Imputed | 0.01203 | 0.2189 |
| rs524161 | 6 | 149636235 | *TAB2* | Imputed | 0.01217 | 1.604 |
| rs12191645 | 6 | 3086489 | *RIPK1* | Genotyped | 0.01225 | 2.139 |
| rs9392453 | 6 | 3086920 | *RIPK1* | Genotyped | 0.01225 | 2.139 |
| rs114090926 | 1 | 12242197 | *TNFRSF1B* | Imputed | 0.01251 | 5.108 |
| rs17884595 | 1 | 12229010 | *TNFRSF1B* | Imputed | 0.01264 | 4.909 |
| rs598492 | 1 | 12234622 | *TNFRSF1B* | Imputed | 0.01264 | 4.909 |
| rs597991 | 1 | 12234767 | *TNFRSF1B* | Imputed | 0.01264 | 4.909 |
| rs597519 | 1 | 12234885 | *TNFRSF1B* | Genotyped | 0.01264 | 4.909 |
| rs141278064 | 4 | 177617892 | *VEGFC* | Imputed | 0.01312 | 0.4076 |
| rs6142164 | 20 | 33006597 | *ITCH* | Imputed | 0.01335 | 0.6458 |
| rs4897110 | 6 | 149603097 | *TAB2* | Imputed | 0.01337 | 1.561 |
| rs1018549 | 14 | 91524400 | *RPS6KA5* | Imputed | 0.01342 | 1.565 |
| rs6088508 | 20 | 33060081 | *ITCH* | Imputed | 0.01345 | 0.6462 |
| rs6915888 | 6 | 149574340 | *TAB2* | Imputed | 0.01353 | 3.285 |
| rs7156015 | 14 | 23319550 | *MMP14* | Imputed | 0.01385 | 1.549 |
| rs1285991 | 14 | 91521261 | *RPS6KA5* | Imputed | 0.01408 | 1.56 |
| rs5746026 | 1 | 12253062 | *TNFRSF1B* | Genotyped | 0.01431 | 6.503 |
| rs17881602 | 1 | 12258103 | *TNFRSF1B* | Imputed | 0.01431 | 6.503 |
| rs17880518 | 1 | 12260399 | *TNFRSF1B* | Imputed | 0.01431 | 6.503 |
| rs17881207 | 1 | 12261613 | *TNFRSF1B* | Imputed | 0.01431 | 6.503 |
| rs17885670 | 1 | 12261790 | *TNFRSF1B* | Imputed | 0.01431 | 6.503 |
| rs11604189 | 11 | 102219863 | *BIRC2* | Genotyped | 0.01436 | 3.331 |
| rs12792649 | 11 | 102220279 | *BIRC2* | Genotyped | 0.01436 | 3.331 |
| rs10895292 | 11 | 102245718 | *BIRC2* | Imputed | 0.01436 | 3.331 |
| rs10895293 | 11 | 102245858 | *BIRC2* | Imputed | 0.01436 | 3.331 |
| rs11225235 | 11 | 102247361 | *BIRC2* | Imputed | 0.01436 | 3.331 |
| rs11601671 | 11 | 102250065 | *BIRC2* | Imputed | 0.01436 | 3.331 |
| rs11225236 | 11 | 102250098 | *BIRC2* | Imputed | 0.01436 | 3.331 |
| rs11225237 | 11 | 102250527 | *BIRC2* | Imputed | 0.01436 | 3.331 |
| rs78166284 | 11 | 102252087 | *BIRC2* | Imputed | 0.01436 | 3.331 |
| rs7113906 | 11 | 102253670 | *BIRC2* | Imputed | 0.01436 | 3.331 |
| rs4911420 | 20 | 32998654 | *ITCH* | Imputed | 0.01445 | 0.6498 |
| rs4911423 | 20 | 32999386 | *ITCH* | Imputed | 0.01445 | 0.6498 |
| rs4277599 | 20 | 33008905 | *ITCH* | Imputed | 0.01445 | 0.6498 |
| rs6058040 | 20 | 33013751 | *ITCH* | Imputed | 0.01445 | 0.6498 |
| rs6059845 | 20 | 33036500 | *ITCH* | Imputed | 0.01445 | 0.6498 |
| rs6059851 | 20 | 33047945 | *ITCH* | Imputed | 0.01445 | 0.6498 |
| rs6059861 | 20 | 33068563 | *ITCH* | Imputed | 0.01445 | 0.6498 |
| rs6059868 | 20 | 33079460 | *ITCH* | Imputed | 0.01445 | 0.6498 |
| rs6059882 | 20 | 33090167 | *ITCH* | Imputed | 0.01445 | 0.6498 |
| rs55710167 | 5 | 67560943 | *PIK3R1* | Imputed | 0.01452 | 1.773 |
| rs9503400 | 6 | 3108907 | *RIPK1* | Genotyped | 0.01459 | 3.834 |
| rs6088483 | 20 | 32984714 | *ITCH* | Imputed | 0.01464 | 0.6505 |
| rs6087579 | 20 | 32985155 | *ITCH* | Imputed | 0.01464 | 0.6505 |
| rs6088484 | 20 | 32985961 | *ITCH* | Imputed | 0.01464 | 0.6505 |
| rs74606085 | 1 | 211544350 | *TRAF5* | Imputed | 0.01473 | 0.4178 |
| rs6059855 | 20 | 33057328 | *ITCH* | Imputed | 0.01476 | 0.6491 |
| rs6059848 | 20 | 33042043 | *ITCH* | Imputed | 0.01481 | 0.6494 |
| rs17586159 | 15 | 66777345 | *MAP2K1* | Genotyped | 0.01523 | 0.1474 |
| rs13044413 | 20 | 33020957 | *ITCH* | Imputed | 0.01536 | 0.637 |
| rs77013687 | 6 | 149574770 | *TAB2* | Imputed | 0.0154 | 4.81 |
| rs17253369 | 5 | 67595509 | *PIK3R1* | Genotyped | 0.01573 | 0.2001 |
| rs6059849 | 20 | 33042143 | *ITCH* | Imputed | 0.01575 | 0.6493 |
| rs6552192 | 4 | 177681130 | *VEGFC* | Imputed | 0.01579 | 0.4192 |
| rs11727268 | 4 | 177681552 | *VEGFC* | Imputed | 0.01579 | 0.4192 |
| rs479676 | 6 | 149633956 | *TAB2* | Imputed | 0.0161 | 1.576 |
| rs6909841 | 6 | 149552430 | *TAB2* | Imputed | 0.01641 | 2.966 |
| rs6910470 | 6 | 149552517 | *TAB2* | Genotyped | 0.01641 | 2.966 |
| rs73602095 | 6 | 149552904 | *TAB2* | Imputed | 0.01641 | 2.966 |
| rs113780485 | 6 | 149553520 | *TAB2* | Imputed | 0.01641 | 2.966 |
| rs6901046 | 6 | 149553940 | *TAB2* | Imputed | 0.01641 | 2.966 |
| rs6921429 | 6 | 149554075 | *TAB2* | Imputed | 0.01641 | 2.966 |
| rs6921436 | 6 | 149554087 | *TAB2* | Imputed | 0.01641 | 2.966 |
| rs6921278 | 6 | 149554090 | *TAB2* | Imputed | 0.01641 | 2.966 |
| rs6921624 | 6 | 149554190 | *TAB2* | Imputed | 0.01641 | 2.966 |
| rs59110359 | 6 | 149554337 | *TAB2* | Imputed | 0.01641 | 2.966 |
| rs614230 | 16 | 57419286 | *CX3CL1* | Genotyped | 0.01645 | 1.536 |
| rs5745971 | 1 | 12235456 | *TNFRSF1B* | Imputed | 0.01651 | 4.653 |
| rs17881148 | 1 | 12235724 | *TNFRSF1B* | Imputed | 0.01651 | 4.653 |
| rs230502 | 4 | 103492383 | *NFKB1* | Imputed | 0.01653 | 0.6441 |
| rs17885967 | 1 | 12237978 | *TNFRSF1B* | Imputed | 0.01656 | 4.65 |
| rs93059 | 4 | 103468518 | *NFKB1* | Imputed | 0.01688 | 0.6486 |
| rs13041646 | 20 | 33082881 | *ITCH* | Imputed | 0.0169 | 0.645 |
| rs117296262 | 16 | 57404050 | *CX3CL1* | Imputed | 0.01695 | 0.07371 |
| rs743925 | 6 | 36094507 | *MAPK13* | Imputed | 0.01697 | 0.3048 |
| rs6141471 | 20 | 33006730 | *ITCH* | Imputed | 0.01698 | 0.6565 |
| rs13107976 | 4 | 177695072 | *VEGFC* | Imputed | 0.0174 | 0.4337 |
| rs560917 | 6 | 149631390 | *TAB2* | Imputed | 0.01755 | 1.565 |
| rs727258 | 14 | 91524968 | *RPS6KA5* | Imputed | 0.01761 | 1.535 |
| rs149802246 | 1 | 12243903 | *TNFRSF1B* | Imputed | 0.01766 | 6.173 |
| rs12030893 | 1 | 9774850 | *PIK3CD* | Imputed | 0.01787 | 5.242 |
| rs17881409 | 1 | 12242759 | *TNFRSF1B* | Imputed | 0.01844 | 0.2766 |
| rs75840657 | 14 | 91427419 | *RPS6KA5* | Imputed | 0.01847 | 1.856 |
| rs4911418 | 20 | 32994257 | *ITCH* | Imputed | 0.01855 | 0.6599 |
| rs6579165 | 20 | 32994715 | *ITCH* | Imputed | 0.01855 | 0.6599 |
| rs4911419 | 20 | 32995873 | *ITCH* | Imputed | 0.01855 | 0.6599 |
| rs3761147 | 20 | 32996689 | *ITCH* | Imputed | 0.01855 | 0.6599 |
| rs4911421 | 20 | 32998948 | *ITCH* | Imputed | 0.01855 | 0.6599 |
| rs4911422 | 20 | 32999345 | *ITCH* | Imputed | 0.01855 | 0.6599 |
| rs2424992 | 20 | 33012060 | *ITCH* | Imputed | 0.01855 | 0.6599 |
| rs6119497 | 20 | 33012529 | *ITCH* | Imputed | 0.01855 | 0.6599 |
| rs6059827 | 20 | 33016358 | *ITCH* | Imputed | 0.01855 | 0.6599 |
| rs6059829 | 20 | 33017074 | *ITCH* | Imputed | 0.01855 | 0.6599 |
| rs6059834 | 20 | 33019299 | *ITCH* | Imputed | 0.01855 | 0.6599 |
| rs6059835 | 20 | 33020141 | *ITCH* | Imputed | 0.01855 | 0.6599 |
| rs6120644 | 20 | 33023810 | *ITCH* | Imputed | 0.01855 | 0.6599 |
| rs1485766 | 4 | 177610884 | *VEGFC* | Genotyped | 2.24E-04 | 0.5185 |
