# Supplementary material for: A genome-wide screen for variants influencing certolizumab pegol response in a moderate to severe rheumatoid arthritis population
Source: PLoS One. 2022 Apr 12;17(4):e0261165. doi: 10.1371/journal.pone.0261165 (PMC9004786; doi:10.1371/journal.pone.0261165)
Supplement: S5 Table — (DOCX) [file pone.0261165.s008.docx]

| Comparison | # Cases | # Controls | # Variants tested | Bonferroni Threshold |
| --- | --- | --- | --- | --- |
| Responders vs. Non-responders | 19 | 55 | 43,182 | 1.16E-06 |
| Responders vs. CHGV controls + Non-responders | 19 | 1601 | 213,042 | 2.35E-07 |
| Non-responders vs. CHGV controls + Responders | 55 | 1565 | 211,734 | 2.36E-07 |
